# A comprehensive review of curcumin-based scaffolds in cartilage tissue engineering

**DOI:** 10.1186/s13287-025-04672-0

**Published:** 2025-09-29

**Authors:** Pardis Yousefi Talouki, Reyhaneh Tamimi, Somayeh Gholami Rudi

**Affiliations:** 1 Department of Biomedical Engineering, QaS.C, Islamic Azad University, Qaemshahr, Iran; 2 Department of Biomedical Engineering, Amirkabir University of Technology, Tehran, Iran; 3 Department of Electrical Engineering, QaS.C, Islamic Azad University, Qaemshahr, Iran

**Keywords:** Curcumin, Cartilage tissue engineering, Cartilage regeneration, Anti- inflammatory, Curcumin-based biomaterial

## Abstract

Articular cartilage’s avascular and aneural nature severely limits its intrinsic regenerative capacity, making injuries and degenerative diseases like osteoarthritis a significant clinical challenge. This review comprehensively examines the paradigm shift towards regenerative medicine strategies, focusing on the integration of the natural polyphenol curcumin into advanced cartilage tissue engineering. While curcumin possesses potent multi-modal therapeutic properties—including anti-inflammatory, antioxidant, anti-catabolic, and chondroprotective effects—its clinical translation is hindered by poor bioavailability and rapid metabolism. We explore innovative biomaterial-based solutions to these limitations, detailing the development of sophisticated DDSs such as nanoparticles, hydrogels (e.g., chitosan, gelatin methacrylate), and synthetic scaffolds (e.g., PCL, PLGA) that enable targeted, sustained release. The review critically analyzes the transition from conventional surgical techniques to emerging therapies like MSC-based treatments, gene therapy, and 3D-bioprinted constructs. Furthermore, we synthesize compelling clinical evidence demonstrating that bioavailable curcumin formulations (e.g., Meriva^®^, Theracurmin^®^) significantly improve pain, stiffness, and functional scores in OA and RA patients. By bridging cutting-edge biomaterial science with the ancient therapeutic wisdom of curcumin, this review highlights a promising frontier in restoring joint integrity and offers a critical roadmap for future research in combinatorial regenerative approaches.

**Graphical abstract:**

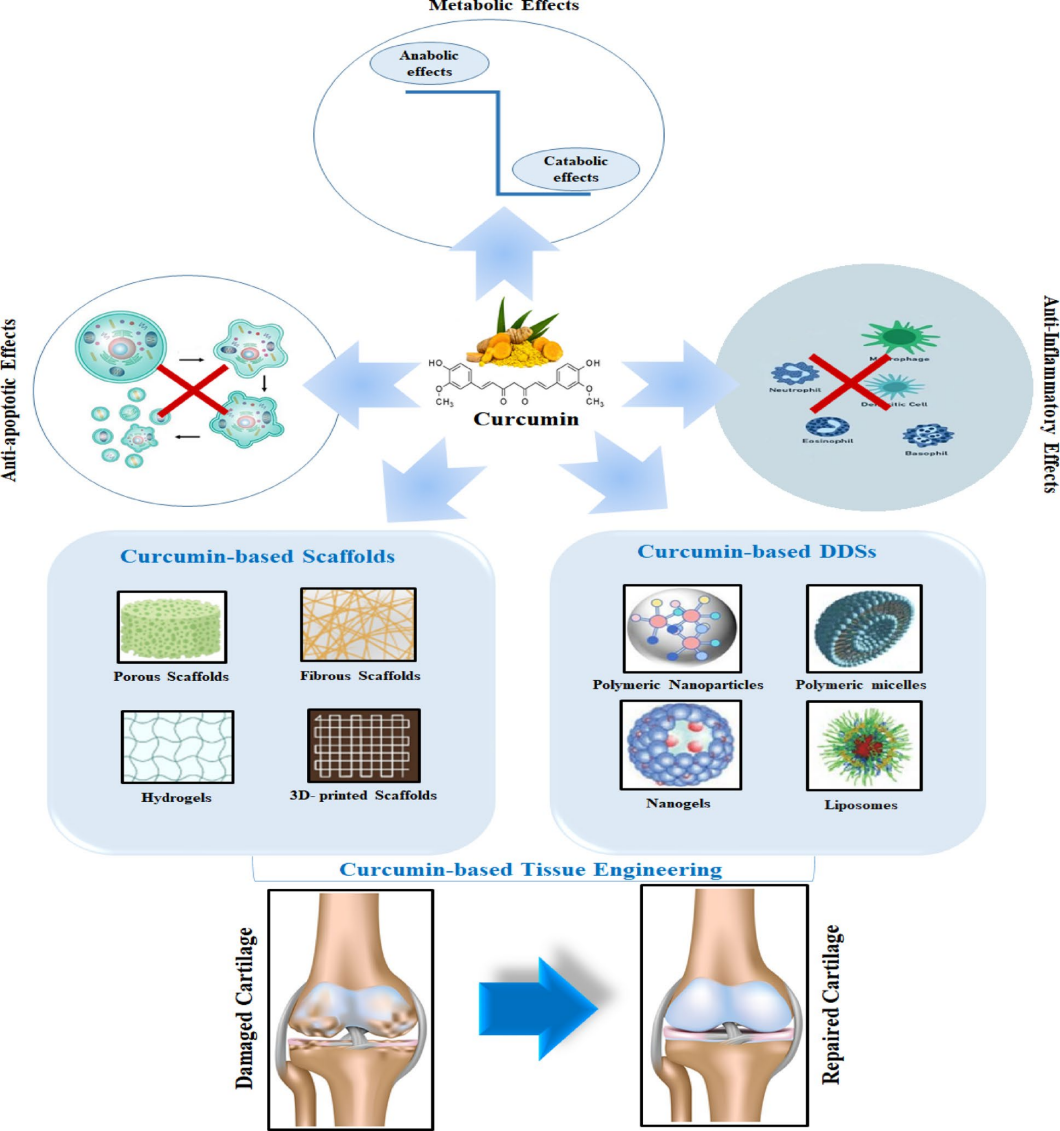

## Introduction

Cartilage is a specialized connective tissue essential for joint function, providing mechanical support, load distribution, and frictionless movement. Structurally, it comprises chondrocytes embedded within an extracellular matrix (ECM) dominated by collagen type II, proteoglycans, and glycosaminoglycans (GAGs) like hyaluronic acid (HA) [1]. Unlike other tissues, cartilage lacks vasculature and innervation, rendering it inherently limited in self-repair capabilities. This vulnerability predisposes it to degenerative and inflammatory diseases such as osteoarthritis (OA) and rheumatoid arthritis (RA) [2]. OA, the most prevalent degenerative joint disorder, involves progressive ECM degradation, chondrocyte apoptosis, and subchondral bone remodeling. Key molecular drivers include upregulated matrix metalloproteinases (MMPs), particularly MMP-13, and a disintegrin and metalloproteinase with thrombospondin motifs (ADAMTS-5), which disrupt collagen (COL) and proteoglycan networks [3]. Conversely, RA is an autoimmune condition marked by synovial hyperplasia, immune cell infiltration, and cytokine-driven cartilage destruction. Pro-inflammatory mediators like Tumor Necrosis Factor alpha (TNF-α), Interleukin-1 beta (IL-1β), and Interleukin-6 (IL-6) activate pathways such as Nuclear factor kappa-light-chain-enhancer of activated B cells (NF-κB) and Mitogen-activated protein kinase (MAPK), perpetuating inflammation and ECM catabolism [4].

Curcumin (Cur), a polyphenol derived from *Curcuma longa*, has garnered attention for its therapeutic potential in cartilage regeneration. Its bioactivity spans multiple mechanisms: (1) anti-inflammatory effects via suppression of NF-κB and cyclooxygenase-2 (COX-2) pathways, reducing IL-1β and TNF-α levels [5]; (2) antioxidant properties through Reactive oxygen species (ROS) scavenging and upregulation of endogenous antioxidants like superoxide dismutase (SOD) [6]; (3) chondroprotection by inhibiting MMPs and promoting COL type II and aggrecan synthesis [7]; and (4) immunomodulation via macrophage polarization toward anti-inflammatory M2 phenotypes. Despite these benefits, curcumin’s clinical translation is hindered by poor bioavailability, rapid metabolism, and low aqueous solubility [8].

To overcome these limitations, cartilage tissue engineering (CTE) strategies have innovatively integrated curcumin into biomaterial scaffolds and Drug Delivery Systems (DDSs). Natural polymers like alginate(Alg) [9], Chitosan (CH) [9], COL [10], HA, and gelatin (Gel) [11]are engineered into hydrogels for pH-responsive or sustained drug release, leveraging their biocompatibility and ECM-mimetic properties [12, 13]. Synthetic platforms, such as polycaprolactone (PCL) and Poly Lactic-Glycolic acid (PLGA), offer tunable degradation rates and mechanical strength, enabling controlled delivery via electrospun nanofibers or 3D-printed matrices. Hybrid systems, including fibrin / PCL composites [14, 15] or decellularized ECM/PCL scaffolds, combine structural stability with bioactivity, often enhanced by curcumin-loaded nanoparticles or microspheres [16] .Advanced DDSs like liposomes and metal-organic frameworks (MOFs) further refine cartilage-specific targeting, exemplified by dCOL2-CM-Cur-PNPs, which home to damaged collagen II sites [17].

Clinical evidence underscores curcumin’s translational promise. Trials with Meriva^®^, a curcumin-phosphatidylcholine complex, demonstrated significant reductions in Western Ontario and McMaster Universities Osteoarthritis Index (WOMAC) scores and inflammatory markers (e.g., IL-6, CRP) in OA patients over 8 months [18]. Similarly, Theracurmin^®^, a nanoparticulate formulation, improved joint function and cartilage stiffness in post-mosaicplasty patients, validated by arthroscopic and MRI analyses [19]. RA studies highlight curcumin’s immunomodulatory efficacy, with reductions in Disease Activity Score 28 (DAS28) and synovitis severity [20]. However, variability in trial outcomes—partly due to dosing inconsistencies—calls for standardized protocols and long-term follow-ups [20, 21]. This review synthesizes the curcumin’s multifaceted therapeutic roles, and cutting-edge CTE advancements. By bridging biomaterial innovation with clinical insights, we aim to accelerate the development of curcumin-based therapies for restoring joint integrity and mitigating degenerative disease progression.

## Curcumin: bridging ancient wisdom and modern medicine

### Curcumin multifaceted therapeutic applications and safety profile

Curcumin, a prominent natural polyphenolic compound with a characteristic yellow-orange hue, is primarily derived from the rhizomes of *Curcuma longa Linn*, a plant associated with the Zingiberaceae and Araceae families. This lipophilic molecule is known for its ability to swiftly permeate cellular membranes, influencing their structure and functionality [22–25]. First isolated from turmeric over 200 years ago, curcumin has a rich history of use in traditional medicine across regions like China, India, and Iran, particularly within Ayurvedic practices. Its therapeutic versatility has made it globally recognized for addressing a wide range of conditions, including diabetes, liver disorders, rheumatoid ailments, atherosclerosis, infectious diseases, and various cancers [26]. Curcumin boasts a strong safety profile, endorsed by the U.S. Food and Drug Administration (FDA), with an acceptable daily intake (ADI) of 0–3 mg per kg of body weight. Historically, its applications in Ayurvedic medicine extended to treating infections, eye inflammations, respiratory and digestive issues, wound care, and even alleviating hallucinations, underscoring its multifaceted medicinal value [26].

### Curcumin structural insights

Curcumin, chemically known as (1E,6E)-1,7-bis(4-hydroxy-3-methoxyphenyl)-1,6-heptadiene-3,5-dione, with the molecular formula C₂₁H₂₀O₆, is a bioactive compound characterized by its unique chemical structure comprising two aromatic rings (phenyl groups) substituted with hydroxyl (–OH) and methoxy (–OCH₃) groups, linked by a seven-carbon chain featuring α,β-unsaturated β-diketone functionality [27]. This diketone group predominantly exists in a stable enol form in solution, contributing to curcumin’s distinctive properties, including its bright yellow color due to the presence of conjugated double bonds and planar structure. Its lipophilic nature, attributed to the aromatic rings and extended conjugation system, allows it to easily traverse cell membranes [9, 28, 29]. Renowned for its therapeutic potential, curcumin exhibits strong antioxidant and anti-inflammatory effects, making it effective in managing inflammatory conditions, metabolic syndrome, pain, degenerative eye diseases, and even certain cancers and neurological disorders [30]. Additionally, it acts as an epigenetic regulator and modulator of protease pathways, further expanding its therapeutic scope. However, curcumin’s clinical application is significantly hindered by its poor solubility and low bioavailability, resulting from limited absorption, rapid metabolism, and swift systemic elimination. To overcome these limitations, various strategies have been developed to enhance its bioavailability by improving its solubility, cellular permeability, and resistance to metabolic degradation, thereby unlocking its full therapeutic potential [28, 30].

### Curcumin extraction methods

The isolation of curcumin, the principal bioactive compound from turmeric (*Curcuma longa* L.), is a critical step that significantly influences its yield, purity, and subsequent application. Extraction methods are broadly classified into conventional and novel (advanced) techniques, each with distinct advantages and limitations.

#### Conventional methods

Conventional techniques, historically the foundation of curcumin isolation, include solvent extraction (maceration), Soxhlet extraction, and hydro-distillation. Solvent extraction involves mass transfer, where a solvent (e.g., ethanol, methanol, acetone, or isopropanol) penetrates the dried, powdered plant matrix, dissolves the curcuminoids, and diffuses out [31, 32]. Ethanol is often the preferred solvent due to its effectiveness and higher safety profile; optimal yields have been achieved using ethanol at 30 °C for 1 h with an 8:1 solvent-to-solid ratio [33, 34].

Soxhlet extraction, invented in 1879, remains a benchmark method [31, 35]. It involves continuous cycling of hot solvent through the solid material, leading to high extraction yields, often near 100% for curcumin [36]. However, it is a lengthy process (e.g., 14 h), requires large volumes of solvent and high energy, and prolonged heating can degrade heat-sensitive compounds [33, 34, 36]. Hydro-distillation is primarily used to produce deodorized turmeric extracts by removing volatile essential oils while retaining the curcuminoid content, demonstrating efficacy comparable to solvent deodorization [33, 37].

#### Novel extraction methods

To overcome the drawbacks of conventional methods—such as long extraction times, high solvent consumption, and compound degradation—novel, eco-friendlier “green” technologies have been developed.

Ultrasound-Assisted Extraction (UAE) utilizes cavitation phenomena generated by sound waves (20–100 MHz) to rupture cell walls, enhancing mass transfer. This method drastically reduces extraction time and solvent use. Optimized conditions using ethanol at 40 °C for 2 h can achieve yields of 73.18%, far more efficient than the many hours required for Soxhlet [36, 38]. Microwave-Assisted Extraction (MAE) uses microwave energy to heat moisture within cells rapidly, creating internal pressure that ruptures the cell walls and releases bioactives. MAE has been shown to outperform both UAE and Soxhlet, yielding higher curcuminoid content (326.79 mg/g) with less energy and shorter times (e.g., < 30 min) [31, 39, 40].

Enzyme-Assisted Extraction (EAE) employs hydrolytic enzymes (e.g., pectinase, cellulase) to break down polysaccharide cell walls, liberating bound metabolites. While environmentally friendly, its main drawback is a long incubation time [41, 42]. Supercritical Fluid Extraction (SFE), often using CO₂, operates at temperatures and pressures above the solvent’s critical point. It is ideal for heat-sensitive compounds due to its low operating temperature, though reported yields for curcuminoids can be relatively low (0.68–0.73%) [43, 44]. Pressurized Liquid Extraction (PLE) uses elevated temperature and pressure to maintain solvents in a liquid state far above their normal boiling points, dramatically enhancing extraction efficiency and speed. PLE can achieve high yields in very short times (e.g., 5–20 min), making it three to six times faster than Soxhlet extraction while using less solvent [31, 45, 46].

The choice of method depends on the desired balance between yield, purity, processing time, cost, and environmental impact. The trend is moving towards these advanced techniques, which offer superior efficiency, reduced ecological footprint, and better preservation of curcumin’s chemical integrity.

### Curcumin synthesis methods

While extraction from turmeric is commercially prevalent, the chemical synthesis of curcumin is crucial for producing high-purity compounds and generating novel structural analogues for research. The development of synthetic routes has progressed through several key milestones [47].

The first successful synthesis was reported by Lampe in 1918, following the initial structural elucidation. This pioneering five-step route began with ethyl acetoacetate and carbomethoxy feruloyl chloride. It proceeded through a series of condensations, saponifications, and decarboxylation steps to build the diferuloylmethane skeleton, ultimately yielding curcumin [47, 48].

A significant simplification was introduced by Pavolini in 1950, who demonstrated that curcumin could be formed in a one-pot reaction by directly condensing two equivalents of vanillin with one equivalent of acetylacetone in the presence of boron trioxide. However, this method suffered from a very low yield of approximately 10% [47, 49].

The major breakthrough came from Pabon in 1964, who vastly improved upon Pavolini’s methodology. By employing a reagent system of boron trioxide, tri-sec-butyl borate, and n-butylamine in ethyl acetate at room temperature, Pabon achieved a dramatically increased yield of nearly 80%. This efficient protocol not only provided superior access to curcumin itself but was also successfully applied to synthesize eight structural analogues, establishing it as a foundational method for generating curcuminoid derivatives [47, 49].

### Curcumin identification methods

Curcumin, the primary curcuminoid derived from turmeric (Curcuma longa), is identified and characterized through various analytical methods that leverage its unique physical and chemical properties. Among these, spectroscopic techniques like UV-Visible (UV-Vis) spectroscopy and Nuclear Magnetic Resonance (NMR) spectroscopy, along with chromatographic methods such as High-Performance Liquid Chromatography (HPLC), are widely employed to analyze curcumin’s molecular structure, purity, and concentration [50–52].

#### High-Performance Thin-Layer chromatography (HPTLC)

HPTLC serves as a reliable technique for quantifying curcumin. This method uses densitometric scanning to determine curcumin concentrations, constructing calibration curves based on peak areas relative to standard concentrations (100–600 ng per band). Validation of the HPTLC method follows International Conference on Harmonization (ICH) guidelines, assessing parameters like linearity, precision, accuracy, limits of detection (LOD), limits of quantification (LOQ), and recovery. Intra-day and inter-day precision analyses confirm the reproducibility of results, as evidenced by low mean relative standard deviations (RSD).HPLC is often the preferred method for curcumin analysis due to its high sensitivity, accuracy, and efficiency. Its simple sample preparation and robustness make it particularly suitable for field applications and laboratories operated by minimally trained technicians. HPLC allows for sharp baseline separation of curcuminoids with low detection limits, making it indispensable for quality control and research involving complex mixtures [53, 54].

#### UV-Vis spectroscopy

Spectrophotometric techniques, including UV-Vis spectroscopy, are also employed for curcumin analysis. UV-Vis spectroscopy offers a rapid and straightforward approach for analyzing colored compounds like curcumin. Its non-destructive nature enables repeated testing on the same sample, with simpler waste disposal compared to other methods. However, it is limited to samples that absorb light in the UV-Vis range and may face interference from impurities or background noise, making it less suitable for low-concentration samples. Despite its limitations, UV-Vis spectroscopy remains a valuable tool for routine analyses due to its ease of use. However, HPLC is often preferred for curcumin characterization because of its superior accuracy and versatility in handling complex mixtures, making it ideal for both research and quality control applications [55–57].

####  Nuclear magnetic resonance spectroscopy

NMR spectroscopy is an advanced analytical method utilized to investigate the molecular structure and behavior of compounds like curcumin. By employing nuclear magnetic resonance, this technique takes advantage of the magnetic properties of atomic nuclei to provide in-depth insights into molecular characteristics, including the identification of functional groups, structural elucidation, and the study of molecular interactions. Furthermore, NMR is instrumental in examining the conformational dynamics and stability of substances under varying environmental conditions [14, 58, 59]. When used alongside complementary techniques such as UV-Vis spectrophotometry and HPLC, NMR spectroscopy enhances the accuracy and depth of compound characterization. Together, these methods offer a robust and comprehensive approach to understanding curcumin and other bioactive molecules with precision [56, 60–62].

## Challenges and innovations in overcoming defects in cartilage regeneration

Cartilage, a unique connective tissue with limited regenerative ability due to its avascularity and low cellular density, presents significant challenges for repair and regeneration. Damage to articular cartilage (AC) disrupts tissue equilibrium, often leading to the release of inflammatory cytokines like IL-1 and subsequent joint degeneration. The International Cartilage Repair Society (ICRS) grading system is a critical tool for assessing cartilage damage, categorized into four grades: Grade I involves superficial damage such as softening or minor irregularities; Grade II affects less than 50% of the cartilage depth; Grade III extends through 50–100% of the cartilage thickness without exposing the bone; and Grade IV represents full-thickness cartilage loss with bone exposure. This classification aids in diagnosing defects, determining treatment strategies, and predicting outcomes. Moderate injuries (Grades II and III) are often treated with surgical techniques like microfracture (MFX) or mosaicplasty, while severe damage (Grade IV) may necessitate more invasive interventions such as artificial joint replacement or osteotomies. Despite advancements in traditional treatments, challenges in cartilage regeneration persist, underscoring the urgent need for innovative therapeutic approaches to address these complex issues effectively [9, 61].

### Traditional treatments: Non-operative methods for managing symptoms

Current non-operative approaches for OA focus primarily on symptom management and slowing disease progression through various pharmacological and biological interventions. These traditional treatments target different aspects of OA pathophysiology, from inflammation reduction to cartilage protection, and are typically employed for early-stage disease. While these modalities can provide meaningful symptomatic relief, their ability to regenerate native cartilage structure remains limited, often necessitating eventual surgical intervention in advanced cases. The following sections examine the evidence for these conventional therapies, including their mechanisms of action, clinical efficacy, and limitations in comprehensive OA management.

#### Non-steroidal anti-inflammatory drugs (NSAIDs)

Non-steroidal anti-inflammatory drugs, including Halofuginone, Etoricoxib, Ibuprofen, and Diclofenac, have demonstrated therapeutic potential for AC regeneration and OA management [9, 63, 64]. These agents exert their pharmacological effects primarily through COX enzyme inhibition, thereby reducing prostaglandin synthesis - key mediators of inflammation and pain perception [65–67]. The NSAID class encompasses both traditional non-selective inhibitors (e.g., ibuprofen, diclofenac) and selective COX-2 inhibitors (e.g., celecoxib), with the latter offering improved gastrointestinal tolerability while maintaining comparable efficacy [68, 69]. Major clinical guidelines, including those from the Osteoarthritis Research Society International (OARSI) and American College of Rheumatology (ACR), recommend NSAIDs as first-line pharmacotherapy for OA-related pain [70, 71]. However, their chronic administration requires vigilant clinical oversight due to dose-dependent risks of gastrointestinal complications, cardiovascular events, and renal impairment [72, 73]. This safety profile has prompted ongoing research into optimized dosing strategies and alternative therapeutic approaches for long-term OA management [65].

#### Hyaluronic acid

Hyaluronic acid intra-articular injections have become a well-established treatment for OA-related joint pain, particularly when NSAIDs or analgesics like acetaminophen prove ineffective. The FDA has approved seven hyaluronate formulations for knee injections: Synvisc, Synvisc-One, Hyalgan, Supartz, OrthoVisc, Euflexxa (formerly Nuflexxa), and Gel-One, with emerging research exploring their potential application in other joints including the hip, shoulder, facet joints, and small joints of the hands and feet [74]. Native hyaluronate remains in the joint space for only 12–24 h post-injection [75], functioning primarily as a viscosupplement that supplements endogenous hyaluronan rather than simply acting as a joint lubricant. This limited residence time has driven the development of enhanced formulations, including high-molecular-weight (HMW) and crosslinked HA preparations that demonstrate extended intra-articular persistence (> 48 h in animal studies) and are gaining clinical acceptance [76, 77] .

The therapeutic benefits of HA injections are multifaceted, encompassing anti-inflammatory effects, mechanical cushioning, pain relief, and stimulation of proteoglycan and GAGs synthesis [78]. Clinical evidence suggests repeated HA injections can effectively maintain or improve knee pain without significant safety concerns [79], with high-molecular-weight HA (HMWHA) demonstrating particular promise by outperforming both conventional NSAIDs and COX-2 inhibitors in OA management [80]. However, while meta-analyses confirm HA’s statistically significant benefits for pain and function compared to placebo, the clinical meaningfulness of these effects remains modest, though the treatment does offer a superior safety profile relative to oral NSAIDs [77, 81].

#### Corticosteroid injections

Corticosteroids have been extensively utilized in both clinical practice and research settings to mitigate joint inflammation [9]. These small (< 700Da), highly hydrophobic molecules can reach the joint space through trans-capillary diffusion following systemic administration, though synovial fluid bioavailability remains significantly lower than systemic concentrations. This pharmacokinetic limitation has driven the development of intra-articular delivery methods, which not only enhance effective dosing but also enable administration of modified corticosteroid formulations with improved joint retention properties that would be unsuitable for systemic use. As a cornerstone of OA management, intra-articular corticosteroids are primarily indicated for joints with refractory pain and/or effusion. While providing symptom relief for up to 4 weeks post-injection [82], concerns about potential cartilage damage and disease progression have led to clinical guidelines recommending no more than 3–4 annual injections per affected joint [77, 83]. Several FDA-approved formulations are available for intra-articular use, including Hydrocortisone tebutate (Hydrocortone-TBA), Betamethasone acetate/sodium phosphate (Celestone Soluspan), Methylprednisone acetate (Depo-Medrol), Triamcinolone acetonide (Kenalog-40), Triamcinolone diacetate (Aristocort Forte), Triamcinolone hexacetonide (Aristospan), and Dexamethasone sodium phosphate [77].

These injections specifically target inflammatory processes and pain associated with conditions like tendinitis and OA. A Cochrane review found they offer moderate pain relief and modest functional improvement, though with a side-effect profile similar to placebo. The evidence quality was rated very low due to study inconsistencies and reliance on small, low-quality trials [84]. While widely used for short-term symptom control, emerging data suggests intra-articular glucocorticoids may be less effective than physical therapy for long-term (1-year) symptom management [85]. This evolving understanding highlights the need for careful consideration of corticosteroid use in OA treatment algorithms, particularly regarding frequency of administration and alternative therapeutic options [65].

#### Natural products

Natural products such as terpenoids, polysaccharides, polyphenols, flavonoids, alkaloids, and saponins are increasingly being investigated for OA treatment due to their multi-target mechanisms, low toxicity, and cost-effectiveness [9]. However, their ability to fully replicate the complex zonal microstructure and composition of AC remains limited. Among these natural compounds, curcumin has shown promise for its anti-inflammatory and analgesic properties. A 12-week study of 70 knee OA patients demonstrated that 1,000 mg daily of Curcuma longa provided greater pain relief than placebo, though the clinical significance was marginal and no differences were observed in effusion-synovitis volume on MRI [86, 87]. Bioavailability challenges have led to the development of enhanced formulations combining curcumin with piperine or BioPerine. Boswellia serrata, traditionally used for its anti-inflammatory effects, showed potential benefits in a meta-analysis of seven trials, though the evidence was limited by low study quality and incomplete adverse event reporting [88]. The efficacy of glucosamine and chondroitin supplements has been particularly inconsistent, with pharmaceutical-grade formulations showing modest statistical benefits over placebo but questionable clinical relevance [89, 90]. Notably, the placebo effect was substantial in trials of these supplements, with approximately 60% of participants reporting meaningful pain reduction regardless of treatment. Omega-3 fatty acids from fish oil demonstrated dose-dependent effects, with lower doses (0.45 g/day) showing better pain and functional outcomes than higher doses (4.5 g/day) over two years, though gastrointestinal side effects were common [91]. Krill oil, while demonstrating modest benefits in mild OA, showed no significant effects in moderate-to-severe cases [92]. Phytoflavonoids have exhibited potential for symptom improvement but carry risks of serious adverse effects, particularly with compounds like flavocoxid that have been associated with liver injury and pneumonitis [93]. While these natural products offer theoretically attractive therapeutic options for OA, most demonstrate only modest efficacy in clinical studies, with inconsistent results and variable safety profiles that warrant further investigation through rigorous, large-scale clinical trials [65] .

####  Platelet-Rich plasma (PRP) therapy

PRP therapy involves concentrating autologous platelets containing growth factors that modulate inflammation, stimulate angiogenesis, and promote tissue repair. While studies consistently demonstrate short- to medium-term analgesic effects of platelet-rich plasma in knee OA, substantial heterogeneity in preparation protocols and administration methods complicates definitive conclusions about its clinical efficacy [94]. Although some clinical studies report improvements in pain and function for cartilage injuries and early OA [95], a comprehensive meta-analysis of 40 trials (*n* = 3,035) found no significant advantage of PRP over hyaluronic acid, intra-articular steroids, or saline placebo for pain relief or functional improvement [96]. Notably, a randomized trial of 288 patients within this meta-analysis showed equivalent outcomes between PRP and saline for both symptomatic relief and structural changes [65, 97].

The therapeutic potential of PRP appears highly dependent on preparation methodology. Current evidence suggests leukocyte-poor PRP prepared via double-spin centrifugation (initial spin at 100×g followed by 1600×g for 20 min each) yields optimal results, achieving platelet recovery rates of 86–99% and approximately 6× baseline concentration while maintaining platelet integrity [98]. Bansal et al. further demonstrated that formulations containing approximately 10 billion platelets may produce sustained benefits in moderate knee OA [99]. However, significant variability in cellular composition, activation protocols, and injection regimens continues to challenge the standardization and reproducibility of PRP therapy in clinical practice [100].

####  Medications

Metformin originally developed for type 2 diabetes management has emerged as a potential disease-modifying agent for OA due to its pleiotropic effects extending beyond glycemic control [101]. Growing evidence suggests its anti-inflammatory properties may help mitigate joint degradation by modulating inflammatory pathways and cellular stress responses, thereby preserving cartilage integrity and soft tissues vulnerable to OA-related damage [102, 103]. These mechanisms could translate to clinically meaningful improvements in pain relief and physical function, positioning metformin as a promising adjunctive therapy for OA [104]. For OA specifically, metformin’s capacity to enhance joint integrity while improving functional outcomes [105] is complemented by its favorable safety profile and low cost. Further research is needed to optimize dosing regimens and validate its efficacy in OA management, but current evidence supports its repurposing as a disease-modifying therapy [65].

While primarily prescribed for dyslipidemia and cardiovascular disease prevention, statins have emerged as a promising therapeutic option for OA due to their pleiotropic effects. Growing evidence suggests these medications may modify OA progression through multiple mechanisms, positioning them as potential disease-modifying OA drugs (DMOADs) [106].

The anti-inflammatory properties of statins appear particularly relevant to OA pathogenesis [107]. By inhibiting pro-inflammatory cytokines (e.g., IL-1β, TNF-α) while upregulating anti-inflammatory mediators, statins may disrupt the chronic low-grade inflammation that drives joint degeneration [108, 109]. This immunomodulatory action creates a more favorable joint microenvironment, potentially slowing cartilage degradation [110].

Notably, statins demonstrate direct chondroprotective effects by stimulating ECM production [111]. Experimental studies show enhanced synthesis of cartilage-specific components including proteoglycans and type II collagen - key structural elements whose depletion characterizes OA progression [112]. These anabolic effects may complement statins’ anti-catabolic actions, collectively preserving joint architecture and function [113].

Emerging evidence suggests statins may benefit the osteochondral unit more broadly [114]. Their positive effects on subchondral bone remodeling could help maintain joint biomechanics, while potential anti-osteoporotic properties may address the bone-cartilage crosstalk implicated in OA pathogenesis. This multi-tissue action makes statins particularly intriguing as they may simultaneously target several pathological features of OA [65, 115].

Emerging research suggests that angiotensin-converting enzyme (ACE) inhibitors and angiotensin II receptor blockers (ARBs), while primarily used to treat hypertension and heart failure, may also benefit OA patients by modulating the renin-angiotensin-aldosterone system (RAAS) [116]. These medications appear to combat OA progression through multiple mechanisms: they reduce joint inflammation and oxidative stress by suppressing angiotensin II-mediated production of pro-inflammatory cytokines [117], help preserve cartilage integrity by inhibiting matrix-degrading enzymes [118], and may maintain synovial fluid homeostasis to improve joint lubrication and mobility. This dual-action potential makes RAAS inhibitors particularly valuable for OA patients who frequently have comorbid conditions like hypertension and metabolic syndrome [119, 120]. By simultaneously addressing cardiovascular risks and joint degeneration, these well-established medications could offer a novel therapeutic approach for managing both conditions [121]. While current evidence remains primarily preclinical, the strong mechanistic rationale and existing safety data support further clinical investigation into repurposing these drugs for OA treatment, potentially providing a comprehensive solution for patients with these interconnected health challenges [65].

Despite their widespread use, current nonoperative OA treatments demonstrate significant limitations in clinical practice. While palliative options like NSAIDs, corticosteroids, and HA provide temporary symptom relief, they generally fail to restore the complex zonal architecture of AC or halt disease progression. Natural products and emerging therapies like PRP show theoretical promise but lack consistent clinical evidence, while repurposed medications (metformin, statins, RAAS inhibitors) offer intriguing disease-modifying potential that requires further validation. This therapeutic gap underscores the need for both improved conservative treatments that can truly regenerate cartilage and better patient stratification to match individuals with optimal therapies. Future research should focus on developing interventions that not only alleviate symptoms but also address the underlying structural degeneration characteristic of OA progression [65].

### Conventional treatments: surgical management of cartilage defects

The surgical management of cartilage defects has evolved significantly, offering both reparative and restorative approaches. Conventional techniques range from marrow stimulation procedures to advanced cell-based therapies, each targeting specific defect characteristics and patient needs. While these methods vary in complexity, they share the common goal of restoring functional articular surfaces. This section critically examines current surgical options, their biological rationale, and clinical outcomes.

#### Microfracture

Full-thickness AC lesions exhibit minimal intrinsic healing potential, necessitating surgical intervention to stimulate repair [122]. Among bone marrow stimulation techniques, microfracture has been extensively studied and refined since its development by Steadman et al. [123, 124]. The procedure involves creating perforations in the subchondral bone, allowing the release of bone marrow components, including Mesenchymal Stem Cells (MSCs), into the defect. These MSCs differentiate into fibrochondrocytes, forming a fibrocartilaginous repair tissue [125, 126].

Technical refinements in MFX have improved outcomes, including removal of calcified cartilage, creation of stable vertical lesion margins, and closely spaced perforations [127]. The resulting marrow clot adheres to the roughened subchondral surface, facilitating tissue formation [128]. However, the repair tissue primarily consists of type I collagen-rich fibrocartilage, which differs biomechanically from native hyaline cartilage (composed predominantly of type II collagen) [129]. Fibrocartilage has inferior load-bearing capacity, reduced GAGs content, and diminished shear resistance, contributing to long-term functional limitations [126, 130].

While MFX demonstrates good short-term clinical outcomes, longer follow-up reveals declining durability, with patient satisfaction decreasing over time [131]. Complications such as subchondral bone overgrowth (25–49% incidence) and incomplete defect filling further limit its efficacy [126, 132]. Additionally, factors such as larger lesion size, advanced age, obesity, and malalignment negatively influence outcomes [133] .

Despite these limitations, MFX remains a first-line option for small, focal defects in carefully selected patients [134]. Combining MFX with corrective osteotomy may improve outcomes in malaligned knees [133]. Future advancements, including biologic augmentation and optimized rehabilitation protocols, aim to enhance repair tissue quality and longevity [100].

#### Autologous Matrix-Induced chondrogenesis (AMIC)

Autologous Matrix-Induced Chondrogenesis is a single-stage cartilage repair technique that combines MFX with the application of a biocompatible scaffold to enhance healing [135]. While MFX alone relies on the formation of a blood clot containing MSCs, the lack of clot stability in larger defects can lead to MSC washout and suboptimal repair. AMIC addresses this limitation by using a collagen I/III membrane or other scaffolds to stabilize the clot, providing a structured environment for MSC differentiation into chondrocytes and promoting hyaline-like cartilage formation [100, 126].

First described by Behrens et al. in 2005, AMIC was developed as an enhancement of microfracture, particularly for larger defects where the clot alone lacks mechanical stability [136]. The scaffold acts as a temporary matrix, supporting cell migration and tissue formation while preventing clot dislodgement [137]. Unlike cell-based therapies (e.g., matrix-induced autologous chondrocyte implantation, MACI), AMIC does not require cell harvesting or in vitro expansion, making it a simpler, more cost-effective single-stage procedure [138].

Clinical studies report improved mid- to long-term outcomes compared to MFX alone. While both techniques show comparable results at 2 years, MFX often exhibits declining function beyond this point, whereas AMIC maintains stable clinical outcomes for up to 5 years [126]. Additionally, AMIC demonstrates better defect filling and tissue quality, particularly in larger lesions [100, 131, 138] .

Despite its advantages, AMIC still results in fibrocartilage or hybrid repair tissue rather than true hyaline cartilage. However, its simplicity, cost-effectiveness, and reproducibility make it an attractive option for focal chondral defects, especially where more complex procedures are not feasible. Future refinements in scaffold design and biologic augmentation may further enhance its regenerative potential [138, 139].

#### Scaffold-Augmented microfracture

Scaffold-augmented MFX represents an advanced evolution of traditional MFX techniques, where biocompatible scaffolds are implanted over the microfractured area to enhance structural support and create an optimal microenvironment for chondrogenesis. This approach addresses key limitations of standard MFX by preventing clot disruption, maintaining defect architecture, and promoting organized tissue regeneration. Three principal scaffold categories have emerged as particularly promising for cartilage repair: hydrogels, biodegradable synthetic polymers, and natural polymers [140].

Hydrogels have gained significant attention due to their remarkable similarity to native cartilage ECM. These three-dimensional, water-rich networks provide excellent conditions for cell attachment, proliferation, and differentiation. Recent advancements have focused on improving their mechanical properties and biofunctionality, with some formulations now incorporating bioactive molecules to further stimulate regeneration. Their tunable physical characteristics allow precise matching with the mechanical demands of specific joint compartments, making them versatile tools in cartilage repair [141].

Biodegradable synthetic polymers, particularly polylactic acid (PLA) and polyglycolic acid (PGA), offer temporary structural support while gradually resorbing as native tissue forms. The development of copolymer systems like PLGA has enabled precise control over degradation rates to align with tissue regeneration timelines. These porous scaffolds typically feature interconnected pore networks (350–550 μm) with optimal porosity (35–45%) for cell infiltration and nutrient exchange. Modern fabrication techniques now allow customization of mechanical properties and architecture to match defect-specific requirements [142].

Natural polymer-based scaffolds utilizing COL, HA, or other ECM components provide inherent bioactivity that promotes cell-matrix interactions [143]. These biomimetic materials support chondrocyte phenotype maintenance and matrix production. Recent processing innovations have enhanced their mechanical resilience while preserving biological functionality, addressing previous limitations in load-bearing applications [100].

The field is now witnessing revolutionary advances with 4D bioprinting technologies that create dynamic, stimuli-responsive scaffolds capable of shape transformation in physiological environments [144]. Particularly noteworthy are magnetic-responsive constructs using silk fibroin-gelatin bioinks that can adapt to irregular defect geometries upon external magnetic field application [145]. These intelligent scaffolds represent a paradigm shift, offering unprecedented integration potential and long-term functional outcomes in cartilage regeneration [100].

#### Biological augmentation with bone marrow aspirate concentrate

Bone marrow aspirate concentrate (BMAC) has emerged as a promising biological augmentation strategy for cartilage repair, particularly when combined with MFX procedures. BMAC is an autologous product rich in MSCs, growth factors, and cytokines that enhance tissue regeneration. Unlike isolated microfracture, which relies on the migration of endogenous MSCs, BMAC delivers a concentrated population of progenitor cells directly to the defect site, potentially improving the quality and durability of repair tissue [100, 146] .

The therapeutic potential of BMAC stems from its unique composition and multifaceted mechanisms of action. Compared to conventional intra-articular treatments like HA and PRP, BMAC offers superior regenerative capacity through its MSC content and immunomodulatory properties [99]. While PRP primarily provides platelet-derived growth factors (PDGF, TGF-β) with short-term effects, and HA functions mainly as a joint lubricant, BMAC promotes cartilage repair through MSC differentiation while simultaneously modulating inflammatory pathways [147]. This dual action addresses both structural degeneration and the inflammatory microenvironment characteristic of OA [100].

Clinical applications of BMAC involve bone marrow aspiration (typically from the iliac crest) followed by centrifugation to concentrate cellular components [99]. When used with microfracture, BMAC is applied directly to the prepared defect, creating a cell-rich environment for tissue regeneration. Preliminary studies demonstrate improved short- to mid-term clinical outcomes compared to MFX alone, with potential to delay more invasive interventions like total knee arthroplasty [25]. The anti-inflammatory effects of MSCs may contribute to these benefits by creating a more favorable environment for cartilage repair [100].

Despite these advantages, challenges remain regarding standardization of preparation protocols, optimal cell dosing, and long-term efficacy [148]. Comparative studies with other biological treatments are needed to better define BMAC’s role in cartilage repair. However, its unique combination of cellular and humoral factors positions BMAC as a compelling option for enhancing MFX outcomes, particularly in cases where more complex cell-based therapies may not be feasible [149].

Recent preclinical studies have demonstrated the enhanced regenerative potential of BMAC when combined with specialized scaffolds. One investigation evaluated cell-derived ECM scaffolds incorporating BMAC, comparing bone marrow MSC-derived (BM-d) and chondrocyte-derived (Ch-d) ECM scaffolds. Both scaffold types effectively promoted chondrogenesis in vitro, with progressive scaffold biodegradation observed over time. In vivo results using nude mice models showed both ECM groups developed cartilage-like tissue with superior integration and mechanical properties compared to MFX alone. While both ECM groups produced hyaline-like cartilage, the BM-d ECM scaffolds showed more significant calcium deposition over time, suggesting enhanced osteochondral potential. After 12 weeks, both ECM groups demonstrated complete defect filling with tissue resembling native cartilage, while the MFX group formed predominantly fibrocartilage [150].

#### Osteochondral autograft transfer system (OATS)

The Osteochondral Autograft Transfer system represents an effective biological approach for repairing focal cartilage defects by transplanting cylindrical plugs of healthy hyaline cartilage and subchondral bone from non-weight-bearing areas to damaged weight-bearing regions [151]. This technique offers several advantages, including rapid osseous integration, immediate structural support with viable chondrocytes, and the ability to treat various lesion sizes (typically < 2 cm², though effective for 2–4 cm² defects in young, active patients) [152]. Histological analyses confirm successful integration of transplanted grafts, maintaining articular surface congruity and preserving hyaline cartilage structure [153] .

Biomechanical studies in animal models demonstrate the procedure’s efficacy. Nakaji et al. [154] reported initial normal cartilage stiffness (107,695.1 ± 11,610.1 N/m²) in rabbit grafts, with temporary decreases during early remodeling phases before returning to baseline by 12 weeks (104,683.7 ± 3,311.5 N/m²). Kuroki et al. [155] found no significant alterations in cartilage stiffness or surface characteristics immediately post-implantation in porcine models. Long-term studies by Lane et al. [156] and Nam et al. [157] showed maintained chondrocyte viability (95%) and superior histological scores (21.6 ± 1.3 at 6 weeks) compared to untreated defects, though with transient biomechanical changes during integration.

Despite its advantages, the OATS technique has limitations. Donor site morbidity remains a concern, with reported rates of 2.3–12.6% [158], particularly for defects > 3 cm² [159]. The finite availability of autologous graft material also restricts its use for larger lesions. However, OATS demonstrates excellent long-term outcomes, with clinical benefits persisting beyond 15 years in appropriately selected cases [160]. When compared to allograft alternatives, OATS provides more reliable integration without risks of immune rejection, making it particularly valuable for smaller defects in young, active patients where preservation of native hyaline cartilage is prioritized [126].

#### Autologous chondrocyte implantation (ACI)

First introduced in 1994, ACI is a two-stage procedure for repairing AC defects [122]. The first stage involves harvesting chondrocytes from a non-weight-bearing area of the joint, which are then expanded in vitro. In the second stage, the cultured cells are implanted into the defect, often covered with a periosteal patch or COL membrane. ACI is particularly effective for large lesions (up to 10 cm²), as it promotes the formation of hyaline-like cartilage with biomechanical properties approaching those of native tissue [161, 162].

Biomechanical studies demonstrate that ACI-repaired tissue can achieve stiffness comparable to healthy cartilage. Brittberg et al. [163] reported graft stiffness values of 2.4 ± 0.3 N (vs. 3.2 ± 0.3 N for normal cartilage), with hyaline-like repairs reaching 3.0 ± 1.1 N—significantly higher than fibrous tissue (1.5 ± 0.35 N). Vasara et al. [161] observed that repaired tissue stiffness averaged 62% of adjacent cartilage, with some cases exceeding 80%, suggesting near-native mechanical function. Henderson et al. [164] found that hyaline-like repairs exhibited stiffness matching or exceeding native cartilage in long-term follow-ups, though excessive stiffness in some cases raised concerns about abnormal load transmission and potential joint dysfunction.

Histologically, ACI yields superior repair tissue compared to microfracture, with hyaline or hyaline-like cartilage predominating in successful cases (65% vs. 28% in fibrocartilage repairs) [165]. However, complications such as periosteal hypertrophy, graft delamination, and reoperation rates remain significant drawbacks. These limitations spurred the development of matrix-induced ACI (MACI), which uses scaffold-based delivery to improve cell retention and reduce surgical morbidity [126].

Despite its regenerative potential, ACI’s two-stage approach, cost, and technical complexity restrict its widespread use. Further refinements in cell culture techniques and scaffold design may enhance its efficacy and accessibility [100].

#### Matrix-Induced autologous chondrocyte implantation (MACI)

MACI represents an advanced evolution of traditional ACI, combining autologous chondrocytes with a biodegradable COL scaffold to enhance cartilage regeneration. This next-generation technique eliminates the need for periosteal harvest by utilizing a biocompatible matrix that supports cell adhesion and proliferation, while allowing implantation via arthroscopy or mini-arthrotomy [166]. Preclinical studies demonstrate MACI’s ability to promote healing in full-thickness defects, with minimal inflammatory response to the scaffold itself [167]. The technique is particularly indicated for lesions > 2 cm², though it may also benefit smaller defects [168].

Biomechanical evaluations reveal MACI’s capacity to restore near-native tissue properties. Lee et al. [169] reported repaired tissue achieving 15% of native cartilage’s aggregate modulus in type II collagen scaffolds, while ovine models demonstrated stiffness reaching 16–50% of healthy tissue [170]. Griffin et al. [171] found MACI-treated equine cartilage recovered 70% of native compressive modulus, with comparable frictional properties (coefficients 0.42–0.52). However, shear modulus remained significantly lower (0.2–0.5 MPa vs. 1.0-1.5 MPa in healthy tissue), potentially affecting long-term durability under shear forces.

Clinically, MACI demonstrates superior outcomes to MFX at 5-year follow-up, with significant improvements in KOOS pain and function scores [126, 172]. The scaffold’s 3D architecture facilitates chondrocyte distribution and phenotype maintenance, yielding more hyaline-like tissue compared to fibrous repair [100]. While avoiding ACI’s common complications like periosteal hypertrophy, MACI retains the limitations of two-stage procedures and requires careful postoperative rehabilitation. Current evidence positions MACI as an effective option for larger defects where MFX proves inadequate, though long-term data beyond 10 years remains limited. Ongoing refinements in scaffold composition and cell-seeding techniques continue to optimize its regenerative potential [126].

**Fig. 1:**
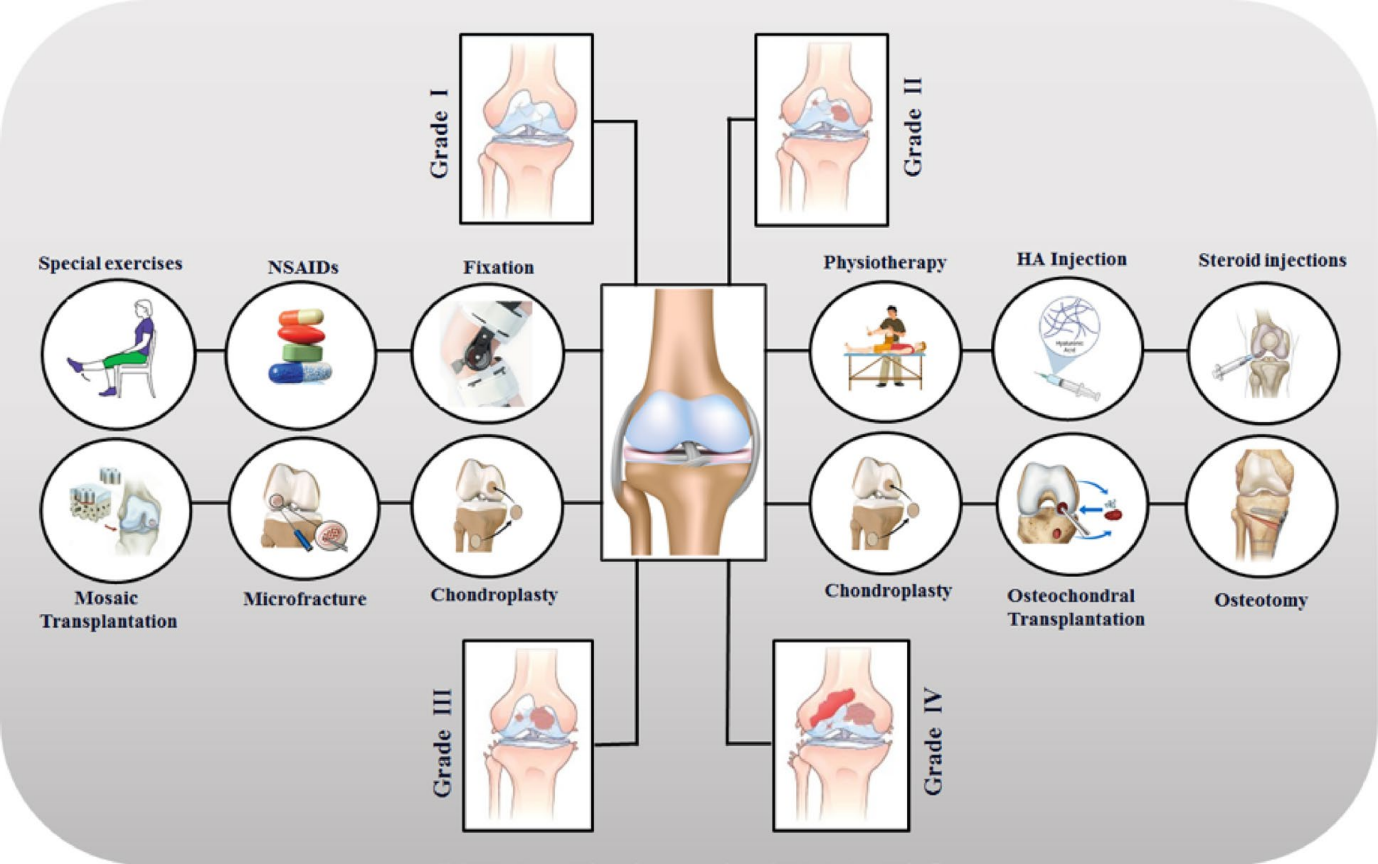
Various treatment strategies to repair different grades of cartilage defects

Current surgical techniques for cartilage repair demonstrate varying degrees of success, with optimal outcomes depending on careful patient selection and defect-specific approach selection. While no single method perfectly replicates native hyaline cartilage, technological advances continue to bridge the gap between biological and functional restoration. The integration of scaffolds, biologics, and minimally invasive approaches represents the next frontier in cartilage repair. Future developments must address long-term durability while improving accessibility and cost-effectiveness of these interventions Fig. 1.

###  Emerging therapies: innovations in cartilage repair

The persistent challenge of cartilage repair is being revolutionized by a new wave of regenerative therapies. Moving beyond palliative care and invasive surgeries, these innovations aim to achieve true biological restoration. This section explores three of the most promising frontiers in this field: cell-based strategies, genetic engineering, and advanced tissue engineering. Together, they represent a paradigm shift towards restoring the intricate structure and function of articular cartilage.

#### MSC -based therapy

MSC-based therapy represents a transformative approach in cartilage regeneration, harnessing the unique ability of MSCs to differentiate into chondrocytes while secreting bioactive molecules that modulate the joint microenvironment [173]. These multipotent cells are derived from various sources including bone marrow (BM-MSCs), adipose tissue (AD-MSCs), and umbilical cord (UC-MSCs), each offering distinct advantages in yield, proliferative capacity, and chondrogenic potential. While bone marrow-derived cells were historically predominant, their invasive harvesting and restricted expansion capability have shifted focus toward adipose-derived MSCs obtained through minimally invasive lipoaspiration and umbilical cord-derived cells demonstrating superior proliferative capacity while maintaining differentiation potential across passages [174]. Therapeutic efficacy is enhanced through hypoxic conditioning (5% O₂), which upregulates chondrogenic markers (SOX9, COL2A1, aggrecan) via HIF-1α stabilization, though prolonged hypoxia requires careful optimization to avoid necrosis. Growth factor supplementation, particularly TGF-β3 and BMP-7, significantly enhances chondrogenesis and matrix synthesis [175], with synergistic effects observed when combined with ghrelin through ERK1/2 and DNMT3A phosphorylation pathways [176]. Small molecules like kartogenin (KGN) provide a stable alternative for inducing chondrogenic differentiation in both BMSCs and synovial MSCs [9, 177].

Emerging cell-free strategies utilizing MSC-derived exosomes demonstrate comparable therapeutic effects through miRNA and protein cargo delivery [178], while co-culture systems with chondrocytes enhance matrix production via paracrine signaling [179]. Clinical meta-analyses report significant pain reduction (VAS: -2.1 ± 0.8; WOMAC: -15.3 ± 4.2 points) and functional improvement (Lysholm: +12.7 ± 3.5 points) at 12-month follow-up [180, 181], though MRI-based cartilage repair outcomes remain inconsistent across studies (WORMS score Δ = 0.84 ± 0.21, *p* = 0.07) [182]. While demonstrating excellent safety profiles (adverse event rate < 3% across 19 trials) [181], challenges persist in donor variability (20–30% non-responders) and standardization of cell dosing (range 10⁶-10⁸ cells/injection) [12, 65]. Current innovations focus on combinatorial approaches using 3D bioprinted scaffolds and oxygen-controlled bioreactors to improve cell retention (> 50% increase vs. suspension injection) [183], with ongoing phase III trials evaluating allogeneic UC-MSC formulations (NCT04241323) and engineered exosomes for targeted delivery [100].

#### Gene therapy

Gene therapy represents a transformative strategy for AC repair, aiming to overcome the limitations of conventional treatments by enabling the sustained, endogenous production of therapeutic factors directly within the joint environment. This approach involves the transfer of nucleic acids encoding therapeutic or regenerative proteins into target cells, facilitating continuous production and site-specific release for long-term treatment of AC injuries [184]. In preclinical models, this method has successfully treated injuries to cartilage, bone, and skeletal muscle by addressing the complex interplay between temporary mechanical incompetence and altered metabolic and inflammatory homeostasis [185, 186].

The therapeutic strategy primarily involves two mechanisms: promoting anabolism and inhibiting catabolism. The overexpression of anabolic factors, such as insulin-like growth factor-1 (IGF-1), members of the fibroblast growth factor (FGF) family, and the transforming growth factor β (TGF-β) superfamily, has proven effective in stimulating chondrogenesis and the synthesis of cartilage-specific matrix components, leading to the formation of hyaline-like repair tissue [187]. Concurrently, the delivery of anticatabolic or anti-inflammatory agents, including interleukin 1 receptor antagonist (IL-1Ra) and interleukins 4 and 10 (IL-4/IL-10), can potently inhibit excessive cartilage degradation [188].

Beyond protein-encoding genes, non-coding RNAs (ncRNAs) have emerged as crucial regulators and promising therapeutic targets. A multitude of ncRNAs, including circular RNAs (e.g., circRSU1, circHIPK3) and microRNAs (e.g., miR-125a-5p, miR-30a-3p), have been shown to intricately regulate cell proliferation, inflammation, apoptosis, and ECM maintenance by modulating key signaling pathways like MEK/ERK and NF-κB through competitive endogenous RNA networks [189–192].

The successful clinical translation of gene therapy is contingent on the development of safe and efficient delivery systems. Polymeric biomaterials are particularly attractive as gene-activated matrices due to their tunable properties, which allow for the sustained and spatiotemporally controlled release of genetic cargo [184, 193]. Viral vectors remain highly effective; for instance, hydrogel-guided delivery of a recombinant adeno-associated virus (AAV) vector coding for IGF-1 enabled long-term cartilage repair and protection against OA in a large animal model [187]. Similarly, lentiviral vectors have been used to encode factors like CLOCK to counteract MSCs decay and promote regeneration [194]. Furthermore, exosomes are being explored as highly absorbable natural vectors for gene delivery [9, 195].

This technology also significantly advances CTE by enhancing the chondrogenic potential of MSCs. Genetic modification, through the overexpression of master regulators like SOX9 or precise gene editing using tools like CRISPR/Cas9 to knockout genes such as RUNX2, can direct differentiation towards a stable chondrogenic lineage and promote the production of a superior quality ECM, thereby improving repair outcomes [100, 196]. Thus, gene therapy offers a powerful tool for the effective, safe, and durable delivery of chondroprotective and chondroregenerative sequences.

#### Tissue engineering

Tissue engineering technology presents a highly viable strategy for AC defect repair, offering a paradigm shift from traditional methods. Since its development in the 1980s, CTE involves the combination of seed cells (e.g., chondrocytes, mesenchymal stem cells), biomaterial scaffolds, and bioactive factors to cultivate a biomimetic tissue in vitro for subsequent implantation and repair in vivo [197]. The relatively simple structure of cartilage, devoid of blood vessels and nerves, made it an early and ideal model for tissue engineering research [88].

The field was pioneered by Vacanti et al. [198], who in 1991 generated cartilage in nude mice using bovine chondrocytes seeded onto a synthetic biodegradable polymer scaffold. This was followed by the landmark creation of a human-shaped ear cartilage by Cao et al. [199] in 1997. These foundational studies paved the way for extensive research in small and large animal models, the latter being critical for clinical translation due to their complete immunogenicity and anatomical similarity to humans [200]. This progress has culminated in several commercial clinical products, such as MACI^®^, CaReS^®^, and BioSeed^®^, which are based on ACI onto matrices and have demonstrated positive repair outcomes [201]. Innovations like the biphasic MaioRegen scaffold, which targets osteochondral defects by mimicking both cartilage and bone layers, can avert two-stage surgeries and simplify the clinical process [9, 202].

A significant challenge in the field is that AC defects, particularly in OA, often extend beyond the cartilage into the calcified cartilage and subchondral bone. Therefore, current research focuses on developing zonal, microstructured scaffolds that can mimic this complex osteochondral unit to achieve complete and integrated repair. The scaffolds themselves are crucial, serving as an artificial ECM to provide structural support and stimulate cellular processes [203]. Ideal scaffolds must possess appropriate mechanical properties to mitigate cell death and promote tissue development [204], and are fabricated from both natural polymers (e.g., alginate, chitosan, collagen, hyaluronic acid) and synthetics (e.g., PLLA/PGA) [126].

Hydrogels are a particularly promising class of biomaterials due to their high-water content, porosity for nutrient transport, and ability to encapsulate cells, encouraging a spherical chondrogenic phenotype and reducing fibrous tissue formation [205]. Their properties, such as polymer chemistry, crosslinking density, and mechanical strength—which can range from under 1 MPa for natural variants to over 100 MPa for synthetic ones—are highly tunable [206]. A critical factor for success is maintaining physioxia (5% O₂) during cell culture, as this physiological oxygen concentration significantly promotes chondrogenesis, enhances GAGs and type II collagen production, and improves the compressive strength of the engineered construct [207, 208]. Despite these advances, challenges remain, including improving the often-limited mechanical strength of hydrogels for load-bearing joints and enhancing integration with the surrounding native tissue [126, 209].

In summary, the convergence of MSCs biology, gene editing, and biomaterial science is forging a new future for cartilage repair. While challenges in standardization, mechanical strength, and integration persist, the progress is undeniable. These innovative approaches are transitioning from laboratory concepts to tangible clinical solutions with demonstrated efficacy. The continued refinement of these therapies promises to finally provide durable and functional restoration for damaged joints.

## Cartilage tissue engineering

Tissue engineering represents a promising avenue for cartilage repair, combining cells, biomaterials, and bioactive molecules to create functional tissue constructs [20]. Recent advancements in cell-based tissue engineering have enabled the integration of diverse cell types, matrices, and bioactive molecules, paving the way for innovative approaches to both AC and osteochondral regeneration. Nonetheless, a deeper understanding of cartilage’s limited healing capacity and the mechanisms underlying its regenerative potential is essential to develop more effective and sustainable therapeutic solutions [9, 210].

### Cell sources in cartilage tissue engineering

Tissue engineering employs various types of seed cells, including autologous chondrocytes, MSCs, and iPSCs, each with distinct advantages and challenges [211].

Autologous chondrocyte transplantation (ACT) is a technique used to repair cartilage defects by implanting reparative chondrocytes [212]. The first generation of ACT used periosteum sealing, but issues such as rupture and hypertrophy led to the adoption of COL membranes in the second generation [213]. While ACT has demonstrated favorable long-term outcomes, challenges remain, including risks of chondrocyte leakage, uneven cell distribution, and the dedifferentiation of chondrocytes during in vitro culture. Additionally, the procedure is costly, technically complex, and requires joint incisions [211, 214]. The third generation of ACT introduced the use of carriers preloaded with chondrocytes to improve delivery and enhance cell survival post-surgery. Despite these advancements, limitations like limited chondrocyte availability and the need for two surgeries persist, highlighting the need for further innovation in this field [215].

MSCs are multipotent and self-renewing cells with significant chondrogenic differentiation potential, making them a valuable resource for cartilage regeneration. Derived from autologous tissues such as bone marrow, adipose tissue, umbilical cord, and synovial tissue, MSCs contribute to local repair and metabolic regulation [216, 217]. However, their therapeutic application faces challenges, including limited migration to injury sites and poor engraftment [138]. To address these limitations, exosomes (Exos), small vesicles secreted by cells, have emerged as a promising alternative. MSC-derived Exos carry bioactive molecules that mediate intercellular communication while avoiding risks like immunogenicity and tumorigenicity associated with cell transplantation [218]. Studies have highlighted the regenerative and immunomodulatory properties of MSC-Exos, demonstrating their anti-inflammatory, anti-apoptotic, and wound healing effects [211, 219].

iPSCs are reprogrammed somatic cells with pluripotency and self-renewal capabilities similar to embryonic stem cells, making them valuable for regenerative medicine and disease modeling [220]. Despite their potential, iPSC-based applications face challenges, including genetic instability due to viral vector use and risks of teratoma formation [221, 222]. iPSCs hold ethical advantages over embryonic stem cells and can be derived from healthy or diseased donors [223]. In chondrogenic differentiation, protocols utilizing factors like TGFβ1, TGFβ3, BMP2, and BMP4 have shown promise, with co-culture systems and small molecule inhibitors reducing off-target differentiation. However, safety concerns persist, and stringent controls are required before clinical approval. While progress has been made in laboratory-scale applications, iPSC-based therapies remain limited to research settings, highlighting the need for further refinement in differentiation protocols and safety measures [211].

### Signaling in cartilage tissue engineering

Chondrocyte differentiation and cartilage repair are regulated by intricate signaling pathways involving key factors such as Wnt, nitric oxide (NO), RA, and protein kinase C (PKC). PKC promotes differentiation through the ERK-MAPK pathway and regulates the chondrocyte phenotype via the actin cytoskeleton [224]. Integrins play a crucial role in cell-matrix interactions, enabling mechano-transduction and force transmission, essential for cartilage tissue engineering [225]. Cytokines and growth factors, including TGF-βs, BMPs, IGF-1, and FGF, are vital for chondrogenesis and ECM metabolism [211, 226], with TGF-β inducing SOX9 synthesis and promoting collagen type II and aggrecan production [227]. BMPs further enhance cartilage synthesis while reducing catabolic cytokine activity [228]. Hypoxia, mediated by HIF signaling, enhances chondrogenesis by upregulating SOX9, COL II, and aggrecan while suppressing hypertrophy markers like COL I and X. Hypoxic conditions also improve GAG retention and HA synthesis in the ECM [229, 230]. Reactive oxygen species and oxygen tension influence chondrocyte metabolism, with hypoxia reducing oxidative stress and altering pH regulation. These complex interactions between signaling pathways, growth factors, and environmental conditions provide promising avenues for advancing CTE and repair strategies [231, 232].

### Scaffolds in cartilage tissue engineering

Scaffolds are influenced by material selection, structural design, and fabrication methods. Both natural and synthetic polymers are widely utilized for these applications. Natural polymer-based hydrogels, often termed “ECM mimics,” incorporate ECM molecules such as COL, GAG, HA, agarose, Alg, fibrin, Gel, CH, and silk fibroin. These materials have been extensively studied due to their biomimetic properties. Synthetic polymers like polyglycolic acid, polylactic acid, polylactide-co-glycolide, polycaprolactone, and polyethylene glycol are also commonly employed [233]. Natural hydrogels degrade through enzymatic activity by cell-secreted enzymes like collagenase, gelatinase, and hyaluronidase, making them particularly suitable for applications requiring biodegradability [234]. Natural materials, while offering benefits such as biocompatibility and adaptability for modifications, often encounter limitations like inadequate mechanical strength and unpredictable degradation rates [235]. To overcome these challenges, integrating synthetic and composite materials is crucial, as they provide improved durability and more predictable performance [232].

#### Scaffold materials for cartilage tissue engineering

Biomaterials play a pivotal role in the success of tissue-engineered constructs for cartilage repair. An ideal scaffold must be biocompatible, promote cellular attachment and proliferation, and possess a degradation rate that aligns with the healing timeline of cartilage defects [236]. Studies indicate that initial cartilage repair occurs within 4–12 weeks post-implantation [237], with complete regeneration observed around 24 weeks [238]. Therefore, scaffolds should maintain structural integrity for 3–6 months to support tissue formation effectively while degrading gradually as new cartilage matures. Mechanical properties are equally critical, as the scaffold should mimic the biomechanical characteristics of native cartilage [239].

Biomaterials for scaffold fabrication fall into three categories: natural, synthetic, and composite materials. Each type offers unique advantages and can be tailored to achieve the desired properties for cartilage tissue engineering. The careful selection and design of these biomaterials are essential to ensure optimal performance and successful outcomes in cartilage regeneration applications [239].

#####  Natural biomaterials

Natural biomaterials derived from plant and animal sources present significant benefits for tissue engineering applications. These materials are recognized for their inherent biocompatibility, biodegradability, and ability to support cell attachment and tissue remodeling processes [239, 240]. A common application of natural biomaterials is in the form of hydrogels, which are characterized by their high-water content and adaptability for injectability. This feature enhances their integration with cartilage repair cells and supports the preservation of the rounded AC phenotype, minimizing differentiation. Additionally, hydrogels are highly valued in studies involving cellular mechanical signaling, as they effectively transmit mechanical signals to encapsulated cells [211, 241]. Despite these advantages, natural biomaterials are not without challenges. They often exhibit low biostability, limited mechanical strength, and the potential to elicit inflammatory responses, which can complicate their use in clinical and research settings. Addressing these limitations remains a critical focus in the development of advanced biomaterials for tissue engineering [240].

*Hyaluronic acid.* HA is a critical component in cartilage repair due to its natural presence in synovial fluid, where it fosters an environment conducive to cartilage regeneration. It aids in the early differentiation of MSCs into cartilage by encouraging the accumulation of proteoglycans and COL2 [113, 211, 242]. Structurally, HA is an acidic glycosaminoglycan composed of D-glucuronic acid and N-acetylglucosamine, with a high molecular weight typically in the millions. Found abundantly in brain nerve tissue, connective tissue, and epithelial cells, HA plays vital roles in joint lubrication, wound healing, and water retention [14]. Recent advancements, such as the development of an electrospun cell-free fibrous HA scaffold by Martin et al., have shown promise. These scaffolds are designed to deliver growth factors like SDF-1α and TGF-β3, which enhance cartilage repair by recruiting mesenchymal progenitor cells and encouraging matrix deposition [243]. Li et al. developed a long-acting injectable hydrogel combining Epigallocatechin-3-0-gallate (EGCG), a green tea-derived antioxidant with strong ROS scavenging properties, and HA, which effectively captures excess ROS. When this hydrogel, loaded with adipose-derived stem cells (ADSCs), was administered via intra-articular injection, it demonstrated significant therapeutic effects. Specifically, it shifted synovial macrophages toward the anti-inflammatory M2 phenotype, reduced the production of proinflammatory cytokines such as IL-1β, MMP-13, and TNF-α, and facilitated the formation of cartilage matrix, suggesting its potential in treating inflammatory joint conditions [244]. Moreover, the incorporation of methacrylate groups into HA allows for light-curing, making it a viable component for bio-ink applications. A commercially produced HA-based scaffold known as Hyaff-11 has shown significant success in cartilage repair. This scaffold exhibits time-controlled degradation, excellent biocompatibility, and the ability to reconstruct cartilage effectively. In studies using ACT with Hyaff-11, histological analyses revealed well-organized hyaline cartilage and ECM formation after 24 weeks. In contrast, untreated defects showed no signs of ECM formation over the same period.These advancements underscore HA’s versatility and potential as a cornerstone in cartilage repair therapies [14].

*Collagen* COL a key structural protein in connective tissue, cartilage, and skin, is highly valued in tissue engineering due to its biodegradability and low antigenicity. Recent studies have highlighted its potential in cartilage repair and scaffold development [211]. For instance, Lim et al. demonstrated that injecting clinical-grade soluble COL1 combined with human nasal septum-derived chondrocytes significantly enhanced cartilage repair in rats compared to COL injection alone [245]. Further advancements were made by Zhang et al., who investigated the impact of pore size in COL porous scaffolds on chondrocyte behavior. Using pre-made ice particulates as a porogen material, they fabricated scaffolds with controlled pore sizes and high porosity, ensuring efficient cell seeding and uniform distribution. In vivo testing in nude mice revealed promising results: histological analysis confirmed cell morphology, while Safranin O staining identified GAGs, and immunostaining detected type II collagen and aggrecan, indicative of cartilaginous tissue formation. Moreover, a correlation was established between scaffold pore size and mechanical properties. Scaffolds with pore sizes ranging from 150 to 250 μm exhibited the highest compressive modulus, suggesting they possess optimal mechanical characteristics for CTE applications. These findings underscore collagen’s significant potential in regenerative medicine and its role in advancing cartilage repair methodologies [246].

*Chitosan* CH is a naturally occurring linear polysaccharide found in the exoskeletons of crustaceans like crabs and shrimps, as well as in the cell walls of fungi. Structurally, it is composed of glucosamine units and contains glycosaminoglycans, which are known to support chondrogenesis and promote cell attachment and growth due to its hydrophilic surface. CH is highly regarded as a scaffold material in CTE because of its excellent biodegradability, antimicrobial properties, and physiological activity [14, 247]. Additionally, due to its structural resemblance to GAGs in the cartilage ECM, CH enhances the resilience of hyaline cartilage against mechanical stresses like shear and compression forces. By modifying its molecular mass and degree of deacetylation, chitosan’s mechanical properties, osteo-inductivity, and immunological compatibility can be further improved, making it a versatile and effective material for biomedical applications [91].

Research by Sadeghian et al. explored how CH concentration and crosslinking mechanisms influence the physical and mechanical attributes of 3D-printed pure CH scaffolds. Their findings highlighted that scaffolds with larger pore sizes exhibited higher swelling rates, while higher chitosan concentrations resulted in reduced swelling rates. This indicates that lower concentrations of chitosan, which promote greater swelling ratios, are more suitable for CTE applications. Moreover, air-dried scaffolds demonstrated superior elastic modulus compared to vacuum-dried ones, with a 10% CH concentration offering optimal printability and interconnected pores essential for tissue integration. The study also confirmed the biocompatibility of these scaffolds, as evidenced by successful chondrocyte attachment [248]. In related advancements, Zhou et al. developed catechol-modified chitosan (CH-C) hydrogels catalyzed by horseradish peroxidase and hydrogen peroxide. These hydrogels were found to support the proliferation and chondrogenic differentiation of BMSCs in vitro. When loaded with BMSCs, the CH-C hydrogels showed superior efficacy in repairing cartilage defects in vivo compared to hydrogels without cell loading [249]. Similarly, Lin et al. created a composite hydrogel scaffold using COL, carboxymethyl chitosan, and Arg-Gly-Asp peptide. This scaffold demonstrated excellent cell adhesion and biocompatibility, effectively enhancing BMSC adhesion and promoting cartilage regeneration [9]. Another promising approach involves combining CH and COL in a 1:1 ratio, as shown by Haaparanta et al., who utilized the freeze-drying method to fabricate cartilage scaffolds. This hybrid scaffold exhibited enhanced mechanical properties, such as improved stiffness, which is crucial for cartilage tissue engineering. Additionally, its high porosity facilitated cell migration and supported excellent chondrocyte viability. These findings collectively emphasize the potential of composite scaffolds to provide robust mechanical support and foster cellular integration for effective cartilage repair applications [250]. Huang et al. developed hydrogels incorporating BMSCs using CH and decalcified bone, demonstrating a notable enhancement in chondrogenic differentiation [251]. Additionally, another research effort designed an innovative CH/nano-hydroxyapatite (nHAP)/ Alg hybrid scaffold through extrusion bioprinting. This method employed an impregnation technique to integrate Alg and nHAP particles into the scaffold. The inclusion of Nhap particles contributed to improved elastic modulus, thermal stability, and cell attachment, fostering a stable physical and chemical microenvironment suitable for chondrocytes. Furthermore, scaffolds impregnated with sodium Alg exhibited enhanced swelling capacity, hydrophilicity, and cell viability, highlighting their potential for advanced tissue engineering applications [252].

*Gelatin* Gel-based scaffolds have shown significant potential in tissue engineering due to their biocompatibility, degradability, and ease of accessibility [253]. For instance, Huang et al. developed a gelatin scaffold doped with HAP using extruded bioprinting techniques. This scaffold supported human umbilical cord blood-derived mesenchymal stem cell (hUCB-MSC) adhesion, migration, and proliferation. The incorporation of nano-HAP into the gelatin matrix allowed modulation of its fluidity and gelation time, optimizing its rheological properties. While HAP doping improved MSC differentiation and cartilage repair efficacy, it slightly reduced cell survival and proliferation rates in vitro [254].

*Silk fibroin* Silk fibroin (SF) is a natural protein material sourced from the secretions of various animals such as silkworms, spiders, and bees. With its remarkable mechanical properties, excellent biocompatibility, biodegradability, and abundant availability, SF has found extensive use in clinical applications, including surgical sutures, artificial blood vessels, and wound dressings [255]. A noteworthy advancement in this field is the creation of an SFG-E7 scaffold by Weili Shi et al., utilizing Direct Ink Writing (DIW) technology. In this innovation, Gel and SF were combined in a 1:1 mass ratio (6.9% w/v), and an E7 peptide was incorporated to optimize both mechanical properties and the degradation rate. The scaffold was designed with a uniform pore size of 350 μm to enhance the proliferation, differentiation, and ECM production of BMSCs. This dual optimization approach has shown significant potential in knee cartilage regeneration by not only attracting and sustaining sufficient BMSCs but also providing mechanical support and a conducive 3D microenvironment for cartilage development [256].

In a separate study, Shi et al. explored the use of 3D-printed scaffolds made from silk-fibroin and gelatin (SFG) for cartilage tissue repair. These scaffolds exhibited favorable degradation rates and mechanical properties tailored for cartilage regeneration. In vitro studies revealed enhanced proliferation and chondrogenic differentiation of BMSCs, along with robust ECM production. The scaffolds supported the formation of hyaline cartilage, as evidenced by elevated levels of COL type II, hydroxyproline, and glycosaminoglycans. Histological and biochemical analyses, including HE staining, toluidine blue staining, and immunohistochemistry for COL type II, confirmed the positive outcomes in tissue morphology and ECM composition. Mechanical testing demonstrated superior reduced modulus and tissue hardness in the SFG scaffolds compared to controls, while histological grading indicated improved repair quality. Overall, the SFG scaffolds excelled in promoting neo-cartilage formation, reducing inflammation, and enhancing mechanical properties for effective cartilage repair [256].

*Alginate* Alg, a polysaccharide derived from brown algae, is a versatile material widely utilized in the biomedical field due to its affordability, availability, biocompatibility, and low immunogenicity. One of its notable properties is its ability to rapidly form gels when combined with divalent cations like calcium ions (Ca2+) makes it a popular bio-ink for extrusion-based bio-printing [257]. Researchers have explored various ways to enhance the mechanical and biological properties of Alg-based hydrogels. For instance, Critchley and colleagues developed fiber-reinforced sodium Alg hydrogel scaffolds using materials like PCL and PLGA to create mechanically supportive network structures. Sodium alginate hydrogels also serve as excellent carriers for bioactive materials such as BMSCs and chondrocytes [258]. In another study, He et al. created a composite hydrogel (Alg/Gel/HA) by combining Alg with Gel and HA. This bio-ink was used to encapsulate cartilage progenitor cells (CPCs) and fibronectin (FN), facilitating cell separation, migration, and proliferation through differential adhesion on FN. The resulting 3D-printed bioactive biofilm was used to enhance the AMIC technique, which is employed to treat cartilage defects. By replacing traditional COL membranes with this biofilm, the approach provided better mechanical support and structural integrity, creating a stable microenvironment for BMSC release from subchondral bone. This innovation improved the thickness and quality of regenerated cartilage and showed promise for clinical applications [197]. Additional studies have demonstrated the potential of Alg scaffolds in cartilage regeneration. Mata et al. investigated the use of human dental pulp stem cells (hDPSCs) seeded in Alg scaffolds for AC repair in New Zealand rabbits. After three months, significant cartilage regeneration was observed [259]. Similarly, Bahrami et al. studied freeze-dried Alg-based constructs and found that higher Alg concentrations enhanced mechanical properties such as compressive modulus. These scaffolds were also compatible with human periodontal ligament stem cells (hPLSCs), further highlighting their versatility in tissue engineering applications [260].

*Chondroitin sulfate* Chondroitin sulfate (CS) plays a significant role in CTE due to its structural and functional properties [176]. As a sulfated glycosaminoglycan composed of N-acetylgalactosamine and glucuronic acid, CS is a critical component of cartilage that binds to proteins to form proteoglycans. This composition provides cartilage with strong compressive resistance and mechanical protection. Beyond its mechanical contributions, CS also promotes cell differentiation [98], enhances anti-inflammatory activity, and supports cartilage recovery, making it a valuable therapeutic agent for OA [14]. However, the rapid degradation of pure CS scaffolds remains a challenge. To address this, researchers have explored innovative approaches. For instance, Li et al. demonstrated that rat ADSCs encapsulated in CS-SH/HB-PEG hydrogel exhibited excellent cell viability, enhanced chondrogenesis, and reduced inflammatory responses, collectively promoting cartilage repair [176]. Similarly, the incorporation of CS-SILY molecules into COL1/2 hydrogels with MSCs significantly boosted cartilage repair potential [261]. In bio-fabrication, Lafuente-Merchan et al. found that adding CS to nanocellulose alginate saline gel improved the rheological properties of bio-inks. This addition increased viscosity while maintaining shear-thinning behavior, enabling better resolution and shape fidelity during scaffold printing. Importantly, the presence of CS enhanced cell survival and chondrogenic differentiation compared to control groups without CS. These findings underscore the versatility of CS in advancing CTE and regenerative medicine [14].

*Decellularized ECM* Decellularized ECM (dECM) has emerged as a promising biomaterial in CTE due to its ability to mimic the native tissue environment. Composed of proteins, glycoproteins, GAGs, and growth factors, ECM provides an ideal scaffold for cell growth and function. By decellularizing native tissues, cells and antigens are removed, leaving behind a 3D ultrastructure that retains the biochemical and mechanical properties of the original tissue. This minimizes immune responses while supporting cell viability, proliferation, and differentiation [262, 263]. Decellularization involves physical, enzymatic, and chemical processes tailored to the tissue type, ensuring the preservation of mechanical integrity while eliminating cellular DNA [232, 264]. dECM can be incorporated into hydrogels, electrospun fibers, or 3D-printed scaffolds, offering flexibility in applications. Studies highlight its use in tissues like cartilage, heart, and blood vessels, demonstrating its potential to replicate native environments. For cartilage engineering, dECM provides a scaffold similar to the natural cartilage matrix, enhancing the likelihood of successful tissue regeneration. Its ability to maintain original components and ultrastructures makes it a valuable tool for repairing injured tissues while reducing the risk of immune rejection [265, 266]. The dECM scaffolds are created using tissues from human, porcine, ovine, and bovine origins, each offering distinct benefits and limitations [267]. The application of crosslinking treatments significantly impacts the mechanical properties of the scaffolds and promotes chondrogenic differentiation by altering cell-matrix interactions [268]. A promising strategy involves combining dECM-derived hydrogels with natural or synthetic materials to form hybrid hydrogels. These hybrids provide enhanced mechanical strength and biological functionality, showcasing considerable potential for use in regenerative medicine through the integration of nano- and microstructures [269].

Zeng et al. have introduced an innovative approach for cartilage repair through the development of injectable pig cartilage-derived dECM hydrogels. This cutting-edge material demonstrates excellent biocompatibility and immunomodulatory properties, as evidenced by both in vitro and in vivo studies. The dECM hydrogels are particularly effective in supporting the chondrogenic differentiation of human urine-derived stem cells (USCs). In in vitro experiments, USCs encapsulated within the dECM hydrogels were observed to survive, proliferate, and generate substantial amounts of cartilage-specific ECM, including COL II and aggrecan. Furthermore, the USCs-laden dECM hydrogels exhibited remarkable potential in promoting ECM secretion, modulating immune responses, and facilitating cartilage regeneration in a rat model of cartilage defects. This promising research highlights a significant advancement in therapeutic strategies for repairing cartilage damage [270].

The study conducted by Guo et al. introduces innovative decellularization strategies for creating reconstituted dECM (rdECM) and biomimetic dECM (bdECM) particles. These particles were incorporated into hyaluronic acid-tyramine (THA) hydrogels and evaluated for their mechanical and stability properties. The novel two-fraction and one-fraction decellularization methods demonstrated superior preservation of proteoglycans and COL, alongside effective DNA removal, compared to traditional approaches.The inclusion of rdECM or bdECM particles in THA hydrogels significantly improved their shear moduli while maintaining shear-thinning behavior. Notably, bdECM particle-embedded hydrogels exhibited excellent long-term stability, retaining GAGs and COL with a swelling ratio of 70%. In contrast, rdECM particle-embedded hydrogels showed limited stability as standalone materials. Additionally, the bdECM-enriched hydrogels demonstrated enhanced compression moduli compared to pure THA hydrogels.Both rdECM and bdECM particles are suitable for integration into THA bioinks for 3D bioprinting of biomimetic cartilage. Hydrogels containing bdECM particles offer a promising solution for cartilage repair due to their natural composition, structural integrity, long-term stability, and improved mechanical performance [9].

Another recent study highlighted the development of an advanced injectable hydrogel combining cartilage dECM and hyaluronic acid methacrylate (HAMA). Using a photo-initiator system of ruthenium (Ru)/sodium persulfate (SPS) and visible light (450 nm), the hydrogel forms within one minute, creating strong bio-adhesive properties due to tyrosine cross-linking. This unique feature eliminates the need for additional chemical modifications. The dual-network structure of the hydrogel offers superior adhesion to cartilage and enhanced mechanical strength compared to Cartilage dECM or HAMA alone. In vitro experiments revealed that the hybrid hydrogel supports chondrogenic differentiation of porcine bone marrow mesenchymal stem cells after 21 days of culture. Furthermore, in vivo tests in a mouse model demonstrated improved biocompatibility over HAMA alone, with successful subcutaneous implantation over two and four weeks. These findings suggest that this hydrogel is a promising biomaterial for cartilage regeneration, potentially enabling minimally invasive treatments through arthroscopic injection in clinical applications [196].

The study by Bordbar et al. highlights the significance of controlled delivery of TGF-β1 and mechanical stimulation in cartilage tissue engineering. Using an ECM-derived hydrogel integrated with TGF-β1-loaded Alg-based microspheres (MSs), researchers promoted chondrogenic differentiation of mesenchymal stromal cells. Physiological conditions were simulated using ex vivo explants and a multiaxial loading bioreactor.The findings revealed that hydrogels containing TGF-β1/MSs were highly cyto-compatible and supported MSC chondrogenesis, showing gene expression levels comparable to other methods of TGF-β1 delivery. However, hydrogels with TGF-β1 directly incorporated showed inferior performance under unloaded conditions. Notably, under mechanical loading, the ECM-derived hydrogel with TGF-β1/MSs produced a superior cartilage matrix in an ex vivo osteochondral defect model compared to controls.This study underscores the importance of localized, controlled TGF-β1 release and mechanical loading for effective neocartilage formation by MSCs. However, further research is required to optimize the process and mitigate the risk of MSC differentiation towards hypertrophy [271].

Researchers have developed a goat ear cartilage-derived biomaterial for repairing osteochondral defects in rabbits. Using chemical decellularization methods (HH buffer and Triton X-100), they successfully removed cells while preserving the cartilage’s structure. When cultured with murine mesenchymal stem cells (MMSCs), the material supported cell infiltration, proliferation, and chondrogenic differentiation, as evidenced by increased ECM protein production (COL and sGAG). Biocompatibility tests showed no immune rejection or tissue necrosis in treated samples, unlike controls. In vivo studies demonstrated that, after 3 months, the implanted constructs significantly promoted regeneration of neocartilage and subchondral bone, as confirmed by histological and micro-CT analysis. Constructs loaded with IGF-1 exhibited superior healing outcomes. This study highlights the potential of decellularized xenogenic cartilage as a biodegradable, bioactive scaffold for AC repair, supporting stem cell growth and enabling effective tissue regeneration [272].

Feng et al. developed an innovative technique to fabricate cartilage-derived extracellular matrix (cECM) and PCL composite nanofibrous membranes. Since cartilage is a dense tissue, they addressed the challenge of electrospinning by processing it into a looser structure through slicing, milling, and digestion. The resulting cECM/PCL nanofibers, with a 50:50 mass ratio, exhibited improved smoothness, uniformity, and mechanical properties compared to pure PCL fibers. Additionally, the hybrid membranes demonstrated enhanced wettability.Importantly, the inclusion of cECM significantly boosted chondrocyte proliferation in vitro and supported cartilage regeneration in vivo. These findings highlight the potential of cECM/PCL nanofibrous membranes as a robust and biocompatible scaffold for cartilage repair. Furthermore, this approach offers a practical and cost-efficient method for synthesizing ECM-based hybrid scaffolds, opening possibilities for broader tissue engineering applications [16].

##### Synthetic biomaterials

Synthetic polymers are pivotal in CTE due to their adaptable compositional, structural, and mechanical properties, which can be tailored for specific applications. Compared to natural polymers, synthetic options offer advantages like reproducibility and longer shelf-life. However, they lack inherent cell adhesion sites, which may hinder their integration into tissue engineering frameworks [239, 273]. These polymers exhibit superior mechanical properties, making them more comparable to natural cartilage in terms of scaffold performance. Common synthetic polymers include PLA, PCL, PEG, PGA, and others like PU, nylon, and silicone rubber. Among these, PLA and PCL have garnered significant attention in CTE due to their biocompatibility and degradability, essential for successful tissue regeneration [14].

*PLA, PGA, and PLGA*. PLA is a biodegradable polymer created through the dehydration condensation of hydroxyl and carboxyl groups from α-hydroxy propionic acid molecules. Known for its high tensile strength and elongation, PLA is widely used in the medical field. Its remarkable property lies in its biodegradability within living organisms, ultimately breaking down into carbon dioxide and water, though the entire process may extend over a year [14]. Derek et al. utilized the FDM method to fabricate PLA and ABS filament-based orthogonal scaffolds with a pore size of approximately 700 microns. These scaffolds were found to support the growth of chondrocytes and nucleus pulposus (NP) cells, facilitating the production of type II collagen and proteoglycans [274]. PGA is an FDA-approved thermoplastic polymer known for its rapid biodegradation cycle, which is significantly shorter compared to PLA. To improve the properties of degradable polymer materials, monomers such as lactic acid and glycolic acid are frequently copolymerized to produce PLGA. The degradation products of PLGA are regarded as safe, exhibiting minimal toxicity and no adverse immunological or rejection reactions in surrounding tissues. These qualities make PLGA a highly valuable material in the field of tissue engineering [14].Chen and colleagues developed a PLGA scaffold exhibiting high biocompatibility, which was seeded with BM-MSCs overexpressing BMP-12 to assess its efficacy in reconstructing rotator cuff tissue and fibrocartilage. Their findings indicated that the application of BMP-12 overexpressing BM-MSCs on 3D-printed PLGA scaffolds significantly enhanced COL organization, promoted fibrocartilage deposition, and notable degradability [275]. In another study, Wei and collaborators utilized PLGA-coated insulin nanoparticles to achieve sustained and delayed drug release. These nanoparticles were adsorbed onto the surface of PCL scaffolds to create a composite structure capable of gradually releasing insulin. This approach not only ensured controlled drug delivery over time but also continuously stimulated Osteo-Chondrogenesis, highlighting the potential of such composite scaffolds in tissue engineering applications [187]. Xuan Zhou and colleagues conducted a similar study where they enhanced the biocompatibility of a primary ink by combining gelatin methacrylate (GelMA) with polyethylene glycol diacrylate (PEGDA), creating what they termed GelMA-PEGDA. To further optimize the ink, they incorporated PLGA microspheres coated with nHAP and TGF-β1. Using stereolithography (SLA) technology, they successfully printed a biphasic gradient scaffold designed to enhance cartilage repair and promote osteogenesis, showcasing significant advancements in tissue engineering applications [276]. Qi et al. conducted research to develop a synthetic scaffold composed of PLGA using a porogen-leaching technique. Their study focused on evaluating the potential of incorporating mesenchymal stem cell (MSC) sheets into the composite scaffold to enhance cartilage regeneration and improve integration between newly formed cartilage and the surrounding native tissue. The scaffold featured a porous structure, and scanning electron microscopy (SEM) analysis confirmed the infiltration of elongated cells into the scaffold pores as well as the attachment of MSCs to the scaffold walls. In vivo experiments using New Zealand white rabbits demonstrated that six weeks post-implantation, the defects showed partial tissue repair, while at 12 weeks, the defects were covered with tissue resembling articular cartilage. The incorporation of MSC sheets into the PLGA scaffolds significantly improved the material’s capacity to regenerate AC and achieve better integration with native tissue compared to PLGA scaffolds containing only MSCs [277].

*PCL* PCL is a polymer material synthesized using caprolactone with diol as an initiator. It exhibits excellent biological compatibility, mechanical properties, and printability, making it suitable for various biomedical applications [278]. Notably, PCL also demonstrates shape memory capabilities, allowing it to return to its original form under specific external conditions [279]. PCL has been approved by the FDA for use in sutures and drug delivery devices. However, certain limitations affect its performance. Its hydrophobic nature can hinder cell adhesion, and its biodegradation cycle is significantly longer than that of polylactic acid, taking approximately 2–3 years [280]. These challenges have prompted researchers to explore various modification techniques to enhance its properties, particularly for applications like cartilage repair. Zare et al. addressed the mechanical limitations of fiber-reinforced hydrogels by incorporating alginate sulfate (Alg-Sul) hydrogel into a PCL framework using FDM printing. This composite scaffold not only mimicked the mechanical properties of natural nasal cartilage but also maintained biological compatibility with human cartilage, highlighting its potential for osteochondral repair and regeneration [281]. Additionally, Pierluca Pitacco et al. developed a 3D-printed scaffold combining hMSCs/fibrin bio-ink with a PCL-reinforced skeleton. The PCL skeleton was treated with 3 M sodium hydroxide (NaOH) for 12 h, enhancing its hydrophobicity and improving adhesion to cell-filled hydrogels [282]. The development of advanced scaffolds for cartilage and subchondral bone repair has seen significant progress through innovative material combinations and fabrication techniques. For instance, PCL scaffolds modified with polydopamine (PDA) enhance hydrophilicity and support PLGA nanoparticle adsorption, achieving high insulin encapsulation efficiency and controlled release while maintaining a porous microstructure and robust mechanical properties [283]. Another research conducted by Critchley and colleagues explored the development of a biphasic composite scaffold composed of PCL and Alg, created through screw extrusion and printing techniques. This process resulted in an orthogonal network structure of PCL fibers. MSCs were encapsulated within an alginate saline gel, which effectively promoted cartilage repair by enhancing cell adhesion and differentiation. The PCL fiber network provided mechanical reinforcement to the alginate saline gel, creating a robust structure capable of supporting significant cartilage formation [258].Yang et al. reported a bifunctional PCL/GelMA/DCECM scaffold (DPGE) co-printed with the aptamer-GE bioink based on a dual-nozzle bio-printer [284]. PCL has large pores of roughly 800–1000 μm, which serve as the backbone to provide mechanical strength and an anatomical structure for the bifunctional scaffold. In the compressive test, the compressive modulus of the DPGE scaffold is 24.62 ± 8.89 MPa, which can withstand physiological compressive stress [285], indicating that the introduction of the PCL framework can provide sufficient biomechanical support and reasonably solve the problem of insufficient mechanical properties of natural bio-ink. Yang et al. introduced a bifunctional PCL/GelMA/DCECM scaffold (DPGE) co-printed with aptamer-GE bioink using a dual-nozzle bio-printer, where the PCL component provides large pores (approximately 800–1000 μm) to ensure mechanical strength and anatomical structure. The DPGE scaffold demonstrated a compressive modulus of 24.62 ± 8.89 MPa, capable of withstanding physiological compressive stress, thereby addressing the mechanical limitations of natural bio-inks [284]. Notably, Shuai et al. utilized SLS as early as 2011 to develop PCL-ceramic particle blends for osteochondral repair [286], while Du et al. designed biomimetic multi-layer scaffolds using PCL and HAP/PCL microspheres with gradient compositions and low material requirements. These cylindrical scaffolds mimic cartilage-to-subchondral bone structures, offering continuous HAP gradients and resolving layer separation issues through SLS-enabled microsphere fusion. With a compression modulus of 8.7 MPa and compressive strength of 4.6 MPa, the scaffolds feature macro-porous structures (400–500 μm pore sizes) and interconnected micropores (60.3% porosity) to enhance cell migration and attachment in vitro [287]. Additionally, GU et al. employed an improved S/O/W emulsion solvent evaporation method to create PCL microspheres of varying sizes (50 μm and 150 μm) for constructing osteochondral scaffolds with non-channel, continuous channel, and non-continuous channel designs, using smaller particles for cartilage regions and larger ones for subchondral bone regions via SLS technology [178].

*PEG* PEG is a polyether polymer produced through the dehydration and condensation of ethylene glycol, characterized by hydroxyl active groups at its ends. These reactive groups enable PEG to interact with a wide range of chemical substances, providing it with outstanding hydrophilicity and modifiability. Renowned for its exceptional biocompatibility and stable mechanical properties, PEG holds great promise in the field of CTE. In particular, PEG-based hydrogels have gained recognition as highly effective scaffold materials for tissue engineering applications, owing to their superior water absorption capabilities, biocompatibility, and capacity to facilitate efficient nutrient transport [211]. Fu et al. engineered block copolymers composed of PEG and PCL using a solvent casting-particulate leaching technique, resulting in scaffolds with an exceptional capacity to enhance stem cell recruitment. Observations through staining revealed that adult stem cells (ASCs) seeded onto these scaffolds exhibited strong attachment and proliferation, demonstrating excellent cell viability. Four weeks after implantation in SD rats, notable repair morphology was observed, though a clear boundary remained between the scaffold and the newly formed tissue. By the eighth week, the cartilage defect was fully repaired, with the regenerated tissue seamlessly covering the entire defect, eliminating any distinction between the scaffold and the native tissue. The PCL/PEG scaffold effectively facilitated the regeneration of tissue, forming a uniform and integrated layer with the surrounding native tissue [288]. Similarly, Bruna et al. explored the use of an interpenetrating polymer network (IPN) hydrogel, constructed from light-induced cross-linked PEG and cationic physically cross-linked Alg, as the primary structure for cartilage scaffolds. Their aim was to create a self-healing support bath to facilitate subsequent functionalized printing structures. This PEG-based IPN hydrogel not only exhibited a high capacity for water absorption but also maintained significant mechanical properties, including rigidity, toughness, and viscoelasticity, making it a promising candidate for cartilage tissue repair applications [289].To enhance cell-hydrogel interactions, researchers such as Yang et al. designed a cell adhesion peptide, cysteine-arginine-glycine-aspartic acid (CRGD), which was covalently crosslinked to PEG hydrogels via a Michael addition reaction. Their findings demonstrated that CRGD significantly improved the interaction between PBMSCs and PEG hydrogels. In particular, PEG hydrogels modified with 1 mM CRGD were shown to optimally promote chondrogenic differentiation, induce macrophage polarization toward the M2 phenotype, and facilitate tissue regeneration and repair [211, 290]. Additionally, another study highlighted the effectiveness of double-network gels composed of PEG, chitosan, and KGN in promoting both the chondrogenic differentiation and viability of PBMSCs, ultimately contributing to the regeneration of AC defects [211]. Advancements in 3D printing technologies, such as SLA, have revolutionized tissue engineering by enabling the fabrication of gradient scaffolds with precise control over material composition and architecture. For instance, SLA has been utilized to create scaffolds using PEG-Da hydrogels embedded with nanocrystalline nHAP and TGF-β1-coated PLGA microspheres. These innovative designs are particularly effective in promoting cartilage repair and facilitating osteogenesis, offering significant potential for regenerative medicine and orthopedic applications [291].

*Other synthetic polymers* In addition to the biopolymers mentioned above, other synthetic polymers and their combinations with various natural or synthetic materials are also used for CTE. For example, PU is recognized as a prominent synthetic biomedical polymer due to its excellent biocompatibility, adaptable chemical structure, and strong mechanical properties, making it a preferred material for cartilage regeneration [292]. As a widely used 3D printing material, PU offers remarkable flexibility, with studies showing that 3D-printed PU stents retain high elasticity even after repeated cyclic loading, closely mimicking the mechanical behavior of soft tissues [293]. However, conventional PU processing often involves toxic organic solvents, which may leave harmful residues in the final product and complicate the fabrication process [294]. To overcome this limitation, biodegradable PU can be produced using water-based methods, as demonstrated by Hung et al. [293] and Wen et al. [295], who developed waterborne polyurethane scaffolds through direct ink writing techniques specifically for CTE applications. Hung et al. utilized a PU and HAP aqueous solution (76:24 mass ratio) to fabricate scaffolds with a compression modulus of 0.33 ± 0.02 MPa and excellent dimensional recovery under cyclic compression strain, enabling MSCs to self-assemble into aggregates and generate cartilage tissue [293]. Wen et al. advanced this approach by creating scaffolds with a higher compression modulus of 0.94 ± 0.01 MPa and impressive biodegradability, retaining approximately 60% of their initial mass after 28 days of in vitro degradation. These innovations underscore the potential of waterborne PU in developing mechanically robust and biodegradable scaffolds for biomedical applications [295].

A recent study introduced innovative scaffolds fabricated from a blend of poly ether sulfone (PES) and PU using a combination of wet-phase inversion and salt-leaching techniques. These scaffolds incorporated nonwoven materials made of gelatin and sodium chloride (NaCl) to create macropores, resulting in a three-dimensional interconnected structure conducive to cellular activity and mobility. The top layers were designed with pores larger than 20 μm, promoting cellular infiltration. Wettability tests, including contact angle and swelling ratio measurements, confirmed the hydrophilic nature of the scaffolds, while mechanical evaluations demonstrated their robustness, with stress resistance surpassing 10 MPa, making them suitable for knee joint applications. Degradation studies in simulated body fluid (SBF) revealed weight loss and structural changes over four weeks, with SEM and MeMoExplorer Software showing an increase in pore size post-degradation. These results suggest that the scaffolds meet the requirements for CTE membranes and merit further testing in animal models [296].

Kim et al. developed hybrid scaffolds combining polyvinyl alcohol (PVA) sponge constructs with PEGDA and either hyaluronic acid (PVA-PHA) or CS (PVA-PCS) to assess their potential for AC regeneration, with a PEGDA-only scaffold (PVA-PEG) serving as the control. These hybrid scaffolds successfully replicated the structural and functional properties of native cartilage and exhibited highly porous structures. In vivo results demonstrated that the PVA-PEG scaffold lacked mechanical strength, while the compressive modulus of the cell-laden PVA-PCS scaffold increased by 80.53% compared to its acellular counterpart. The PVA-PHA scaffold showed the greatest mechanical improvement, with increases of 210% and 78.83% compared to the acellular PVA-PHA scaffolds. Histological evaluations revealed enhanced lacunae formation, round chondrocytes, and ectopically deposited ECMs in the PVA-PCS and PVA-PHA scaffolds, as confirmed by HE and Safranin O staining. Immunofluorescence further validated the presence of type II collagen, linking the improved mechanical properties of these scaffolds to ectopic cartilage formation. These findings highlight the potential of PVA-based scaffolds, particularly PVA-PHA and PVA-PCS, for cartilage repair applications [239, 297].Poly (1,3-trimethylcarbonate) (PTMC) is a flexible, biodegradable, and amorphous polymer synthesized via ring-opening polymerization of 1,3-trimethylcarbonate. Despite its ability to maintain mechanical strength during enzymatic surface erosion and its lack of acidic byproducts during degradation—an advantage for tissue reconstruction—PTMC has low initial mechanical strength [176, 298] and is highly hydrophobic, hindering cell adhesion [299]. To address these limitations, Liu et al. enhanced PTMC’s mechanical properties by creating cross-linked networks using gamma radiation with crosslinking agents like trimethylolpropane triacrylate (TMPTA), pentaerythritol triacrylate (PETA) and pentaerythritol tetraacrylate (PET4A), achieving a tensile strength of 25.95 ± 2.20 MPa. However, the degradation rate remained slow, retaining over 90% of mass after 12 weeks of enzymatic degradation [176]. Additionally, Thomas et al. developed gAR-graft-PTMC (gel-g-PTMCn), significantly improving tensile Young’s modulus compared to PTMC or Gel. They utilized SLA to fabricate 3D porous structures with high shape accuracy and a layer thickness of ~ 100 μm by diluting the resin with 40% DMSO to achieve suitable viscosity. These advancements highlight PTMC’s potential in tissue engineering while addressing its inherent limitations [300].

##### Composite biomaterials

Synthetic polymers offer exceptional control over physicochemical properties, robust mechanical performance, and ease of processing. However, they can provoke undesired inflammation and tissue formation. In cartilage tissue engineering, designing scaffolds with optimal biochemical and biomechanical properties remains a significant challenge [232]. To address the limitations of both natural and synthetic polymers—such as the weak mechanical strength of natural biomaterials and the insufficient cell adhesion sites in synthetic ones—researchers have explored combinations of these materials in various concentrations. These hybrid bio-composite materials aim to achieve a balance between biocompatibility, degradation rates, and mechanical integrity. Recent advancements highlight the potential of such composites to overcome individual shortcomings, paving the way for more effective tissue-engineered constructs [301]. Various composite scaffolds have been explored for cartilage tissue engineering, demonstrating promising results in both in vitro and in vivo studies. Liu et al. developed a 3D cartilage scaffold combining poly (L-lactic acid) (PLLA) and Alg through electrospinning, with COL serving as an adhesive layer between piezoelectric PLLA nanofiber films. This construct generated controlled piezoelectric charges under load, absorbed proteins effectively, and promoted cartilage healing when implanted into rabbit knee joints, with defects being filled with neo-cartilage tissue and higher subchondral bone volume observed after 1–2 months [302]. Kundu et al. utilized extrusion-based bioprinting to create a scaffold of Alg and PCL, showing 85% chondrocyte viability and enhanced ECM production with TGF-β, leading to cartilage formation in vivo within 4 weeks [303]. Torricelli et al. fabricated PLLA-gelatin composite scaffolds via electrospinning, which exhibited intermediate tensile properties, supported chondrocyte attachment and proliferation, and promoted mineralization while maintaining a fibrous structure [304]. Thiem et al. designed gelatin-PLGA scaffolds using freeze-drying, which demonstrated high chemical stability in PBS, complete degradation within 8 weeks in vivo, and good cell proliferation with porcine chondrocytes, leaving behind fibrous tissue without adverse effects. These studies highlight the potential of composite biomaterials in advancing cartilage regeneration [305].

Biologically inspired elastomers play a crucial role in cartilage tissue engineering, leveraging materials like polydiol citrate (POC) [306], polyglycerol sebacate (PGS) [307, 308], and polyglycerol dodecane dicarboxylate (PGD) [309]. POC, a biocompatible elastic polyester, exhibits tunable mechanical properties and degradation rates but faces challenges like weak tensile strength and pH-reducing degradation products [310]. These issues have been mitigated by blending POC with chitosan for improved strength and pH stability or incorporating Ag-containing mesoporous bioactive glass (Ag-MBG) to enhance crosslinking density and antimicrobial properties [311]. PGS, synthesized from glycerol and sebacic acid, is non-immunogenic and mimics cartilage viscoelasticity [32, 308]. PEGylated PGS (PEGS) combined with MBG has shown promise in simultaneous cartilage and subchondral bone repair, achieving remarkable regeneration in rabbit models [32]. PGD, a biodegradable polyester with shape memory properties, offers tunable elasticity but requires harsh curing conditions [312]. Photocurable PGD (APGD) addresses this limitation, facilitating in vivo applications and maintaining good cell viability. APGD has also shown potential for 3D-printed scaffold structures, broadening its applicability in tissue engineering. Collectively, these advancements highlight the versatility of elastomers in addressing complex challenges in cartilage regeneration [309].

Table 1 provides an overview of various biomaterial combinations, cell types, fabrication methods, and their therapeutic outcomes in the field of cartilage tissue engineering. It emphasizes the utilization of both natural and synthetic polymers, such as HA, CH, COL, PCL, and PLGA, in conjunction with stem cells like ADSCs, MSCs, BMSCs, or chondrocytes. The findings reveal significant achievements, including reduced inflammation, enhanced mechanical properties, improved chondrogenic differentiation, and effective cartilage regeneration in preclinical models. Advanced fabrication techniques, including injectable hydrogels, 3D bioprinting, electrospinning, and decellularized ECM scaffolds, are highlighted for their ability to mimic native tissue structures, enable controlled drug delivery, and achieve seamless functional integration with surrounding tissues.

**Table 1:** The overview of various biomaterial combinations, cell types, fabrication methods, and their therapeutic outcomes in cartilage regeneration

Scaffold materials	Cell type used	Fabrication method	Results	Refs.
HA / (EGCG)	Adipose-Derived Stem Cells	Injectable Hydrogel	Shifted synovial macrophages toward the anti-inflammatory M2 phenotype, reduced proinflammatory cytokines (IL-1β, MMP-13, TNF-α), and facilitated cartilage matrix formation. Effective in treating inflammatory joint conditions.	[98]
HA /MAGroups	Mesenchymal Stem Cells	Light-Curing Hydrogel	Enabled bio-ink applications, improved mechanical properties, and supported cartilage repair.	[15]
COL / Human Nasal Septum-Derived Chondrocytes	Human Nasal Septum-Derived Chondrocytes	Injectable Scaffold	Significantly enhanced cartilage repair in rats compared to collagen injection alone.	[99]
COL/ Ice Particulates	Chondrocytes	Porous Scaffolds with Controlled Pore Size	Scaffolds with pore sizes of 150–250 μm exhibited the highest compressive modulus, indicating optimal mechanical properties for cartilage tissue engineering. Promoted cartilaginous tissue formation in vivo.	[100]
CH / BMSCs	Bone Marrow-Derived Stem Cells	Catechol-Modified Hydrogels	Supported proliferation and chondrogenic differentiation of BMSCs in vitro. Superior efficacy in repairing cartilage defects in vivo compared to non-cell-loaded hydrogels.	[104]
COL / Carboxymethyl Chitosan / Arg-Gly-Asp Peptide	Bone Marrow-Derived Stem Cells	Composite Hydrogel Scaffold	Demonstrated excellent cell adhesion and biocompatibility, enhancing BMSC adhesion and promoting cartilage regeneration.	[105]
CH / COL	Chondrocytes	Freeze-Drying	Enhanced mechanical properties (e.g., stiffness) and high porosity, facilitating cell migration and chondrocyte viability. Effective for cartilage repair applications.	[106]
CH/ Decalcified Bone	Bone Marrow-Derived Stem Cells	Hydrogel	Notable enhancement in chondrogenic differentiation, supporting cartilage repair.	[107]
CH / nHAP / Alg	Chondrocytes	Extrusion Bioprinting	Improved elastic modulus, thermal stability, and cell attachment. Enhanced swelling capacity, hydrophilicity, and cell viability, suitable for advanced tissue engineering applications.	[108]
Gel / nHAP	Human Umbilical Cord Blood-Derived MSCs	Extruded Bioprinting	Improved MSC differentiation and cartilage repair efficacy, though slightly reduced cell survival and proliferation rates in vitro.	[110]
SF / Gel /E7 Peptide	Bone Marrow-Derived Stem Cells	Direct Ink Writing	Enhanced proliferation, differentiation, and ECM production of BMSCs. Provided mechanical support and a conducive 3D microenvironment for cartilage development.	[112]
Alg/ Gel / HA	Cartilage Progenitor Cells	3D-Printed Biofilm	Improved mechanical support and structural integrity, enhanced cartilage regeneration, and provided a stable microenvironment for BMSC release.	[115]
Alg	Human Dental Pulp Stem Cells	Alginate Scaffolds	Significant cartilage regeneration observed in New Zealand rabbits after three months.	[116]
Alg	Human Periodontal Ligament Stem Cells	Freeze-Dried Constructs	Higher alginate concentrations enhanced mechanical properties (e.g., compressive modulus) and demonstrated compatibility with hPLSCs.	[117]
CS	Rat Adipose-Derived Stem Cells	CS-SH/HB-PEG Hydrogel	Enhanced chondrogenesis, reduced inflammatory responses, and promoted cartilage repair.	[118]
CS / MSCs	Mesenchymal Stem Cells	COL1/2 Hydrogels with CS-SILY Molecules	Significantly boosted cartilage repair potential.	[119]
Pig Cartilage-Derived dECM Hydrogels	Human Urine-Derived Stem Cells	Injectable Hydrogel	Supported chondrogenic differentiation, promoted ECM secretion, modulated immune responses, and facilitated cartilage regeneration in a rat model. Excellent biocompatibility and immunomodulatory properties.	[122]
rdECM / THA	Mesenchymal Stem Cells	3D Bioprinting	Improved shear moduli and mechanical stability. Suitable for biomimetic cartilage repair with enhanced mechanical performance.	[129]
Cartilage dECM / HAMA	Porcine Bone Marrow Mesenchymal Stem Cells	Injectable Hydrogel	Superior adhesion to cartilage, enhanced mechanical strength, and supported chondrogenic differentiation. Improved biocompatibility in vivo. Potential for minimally invasive treatments.	[130]
ECM-Derived Hydrogel / TGF-β1-Loaded Alginate Microspheres	Mesenchymal Stromal Cells	Hydrogel with Microspheres	Promoted chondrogenic differentiation under mechanical loading. Produced superior cartilage matrix in ex vivo osteochondral defect models.	[131]
Goat Ear Cartilage-Derived dECM	Murine Mesenchymal Stem Cells	Chemical Decellularization	Supported cell infiltration, proliferation, and chondrogenic differentiation. Promoted regeneration of neocartilage and subchondral bone in rabbits.	[132]
cECM / PCL	Chondrocytes	Electrospinning	Improved smoothness, uniformity, and mechanical properties. Enhanced chondrocyte proliferation in vitro and cartilage regeneration in vivo. Cost-efficient method for ECM-based hybrid scaffolds.	[17]
PLA / ABS Filament	Chondrocytes, Nucleus Pulposus Cells	Fused Deposition Modeling	Supported cell growth, facilitated production of type II collagen and proteoglycans.	[134]
PLGA /BMP-12 Overexpressing BM-MSCs	Bone Marrow-Derived Mesenchymal Stem Cells	3D Printing	Enhanced collagen organization, promoted fibrocartilage deposition, and notable degradability.	[135]
PLGA / Insulin Nanoparticles / PCL	Mesenchymal Stem Cells	Adsorption onto PCL Scaffolds	Controlled drug delivery, stimulated osteo-chondrogenesis, and improved cartilage repair.	[136]
GelMA / PEGDA / PLGA Microspheres (nHA / TGF-β1)	Mesenchymal Stem Cells	Stereolithography	Enhanced cartilage repair and osteogenesis, improved biocompatibility.	[137]
PLGA + MSC Sheets	Mesenchymal Stem Cells	Porogen-Leaching Technique	Improved cartilage regeneration and integration with native tissue. Partial tissue repair at 6 weeks and complete coverage at 12 weeks in rabbit models.	[138]
PCL / Alg-Sul	Human Mesenchymal Stem Cells	Fused Deposition Modeling (FDM)	Mimicked mechanical properties of natural nasal cartilage, maintained biological compatibility, and showed potential for osteochondral repair.	[142]
PCL / Fibrin Bio-ink	Human Mesenchymal Stem Cells	3D Printing	Enhanced hydrophobicity, improved adhesion to cell-filled hydrogels, and promoted cartilage repair.	[143]
PCL / Alg	Mesenchymal Stem Cells	Screw Extrusion and Printing	Orthogonal network structure of PCL fibers provided mechanical reinforcement, promoting cartilage repair through enhanced cell adhesion and differentiation.	[114]
PCL / GelMA / DCECM (DPGE)	Mesenchymal Stem Cells	Dual-Nozzle Bioprinting	Large pores (800–1000 μm) provided mechanical strength and anatomical structure. Compressive modulus of 24.62 ± 8.89 MPa, addressing mechanical limitations of natural bio-inks.	[144]
PCL / Ceramic Particles	Osteochondral Cells	Selective Laser Sintering	Mimicked cartilage-to-subchondral bone structures, offering continuous HA gradients and resolving layer separation issues. Compression modulus of 8.7 MPa and compressive strength of 4.6 Mpa.	[146]
PCL + HAP/PCL Microspheres	Osteochondral Cells	Selective Laser Sintering	Biomimetic multi-layer scaffolds with gradient compositions. Macro-porous structures (400–500 μm) and interconnected micropores (60.3% porosity) enhanced cell migration and attachment.	[147]
PEG / PCL	Adult Stem Cells	Solvent Casting-Particulate Leaching	Enhanced stem cell recruitment, strong cell attachment, and proliferation. Full cartilage repair observed after 8 weeks in SD rats.	[149]
PEG / Alg	Mesenchymal Stem Cells	Light-Induced Cross-Linking	High water absorption, rigidity, toughness, and viscoelasticity. Promising candidate for cartilage tissue repair.	[150]
PEG + CRGD Peptide	Peripheral Blood Mesenchymal Stem Cells	Michael Addition Reaction	Improved cell-hydrogel interactions, promoted chondrogenic differentiation, induced macrophage polarization toward M2 phenotype, and facilitated tissue regeneration.	[151]
PEG / CH / KGN	Peripheral Blood Mesenchymal Stem Cells	Double-Network Gels	Promoted chondrogenic differentiation and cell viability, contributing to articular cartilage defect regeneration.	[63]
PEG-Da / nHAP / TGF-β1-Coated PLGA Microspheres	Mesenchymal Stem Cells	Stereolithography	Effective in promoting cartilage repair and osteogenesis. Significant potential for regenerative medicine and orthopedic applications.	[152]
PU / HA	Mesenchymal Stem Cells	Water-Based Direct Ink Writing	Compression modulus of 0.33 ± 0.02 MPa, excellent dimensional recovery, and enabled MSC self-assembly into aggregates for cartilage tissue generation.	[154]
PES / PU / Gel / NaCl	Mesenchymal Stem Cells	Wet-Phase Inversion + Salt Leaching	Robust mechanical properties (stress resistance > 10 MPa), hydrophilic nature, and suitable for knee joint applications.	[157]
PVA / PEGDA / HA	Chondrocytes	Hybrid Scaffold Construction	Greatest mechanical improvement (210% increase in compressive modulus), enhanced lacunae formation, and ectopic cartilage formation	[158]
PTMC / Crosslinking Agents (TMPTA, PETA, PET4A)	Mesenchymal Stem Cells	Gamma Radiation Cross-Linking	Improved tensile strength (25.95 ± 2.20 MPa), but slow degradation rate (retained > 90% mass after 12 weeks).	[118]
PTMC / Gelatin (gel-g-PTMCn)	Mesenchymal Stem Cells	Stereolithography	Improved tensile Young’s modulus, high shape accuracy, and suitable viscosity for 3D porous structures.	[161]
PLLA / Alg / CoL	Chondrocytes	Electrospinning	Generated controlled piezoelectric charges, absorbed proteins effectively, and promoted cartilage healing in rabbit knee joints. Neo-cartilage tissue formation and increased subchondral bone volume observed.	[148]
Alg / PCL	Chondrocytes	Extrusion-Based Bioprinting	85% chondrocyte viability, enhanced ECM production with TGF-β, and cartilage formation in vivo within 4 weeks.	[163]
PLLA / Gel	Chondrocytes	Electrospinning	Intermediate tensile properties, supported chondrocyte attachment and proliferation, and promoted mineralization while maintaining a fibrous structure.	[164]
Gel / PLGA	Porcine Chondrocytes	Freeze-Drying	High chemical stability in PBS, complete degradation within 8 weeks in vivo, and good cell proliferation. Fibrous tissue formation without adverse effects.	[165]
POC / CH	Mesenchymal Stem Cells	Blending	Improved tensile strength and pH stability, enhanced mechanical properties, and biocompatibility.	[171]
PGS / MBG	Mesenchymal Stem Cells	Combination with MBG	Simultaneous cartilage and subchondral bone repair, remarkable regeneration in rabbit models.	[167]
PGD (Photocurable APGD)	Mesenchymal Stem Cells	3D Printing	Tunable elasticity, good cell viability, and potential for 3D-printed scaffold structures.	[169]
PEGS / MBG	Mesenchymal Stem Cells	Combination with MBG	Enhanced cartilage and subchondral bone repair, improved mechanical properties, and biocompatibility.	[172]

##### Curcumin-based scaffolds and drug delivery systems for cartilage regeneration

Curcumin, known for its significant anti-inflammatory and chondroprotective properties, faces challenges in clinical applications due to its poor solubility, low bioavailability, and rapid metabolism. To address these limitations, various natural and synthetic biomaterials, along with their composites, have been investigated to create advanced DDSs tailored for TE applications [313] .We have summarized curcumin-based scaffolds and DDSs for cartilage regeneration in Table 2.

CH has been widely utilized in curcumin-based DDSs due to its favorable properties. While CH hydrogels enable localized delivery of curcumin, their low encapsulation efficiency and rapid drug release caused by quick degradation hinder their effectiveness in cartilage tissue engineering [314].

CH combined with Montmorillonite (MMT) was used to create hydrogels aimed at enhancing the performance of curcumin molecules. Subsequently, curcumin and silver (Ag) nanoparticles were incorporated into the synthesized hydrogel. Research findings revealed that the drug release behavior of the CH/MMT/curcumin system is highly influenced by the pH of the surrounding environment and the characteristics of the Ag nanoparticles. Additionally, curcumin and Ag nanoparticles underwent treatment with oxygen plasma, a clean, simple, and environmentally friendly approach. Results demonstrated that oxygen plasma effectively enhances both the antibacterial properties and drug release efficiency of curcumin molecules in hydrogels. Notably, prolonged exposure to oxygen plasma further amplified these beneficial effects, making it a promising method for optimizing the functionality of such DDSs [315].

**Fig. 2:**
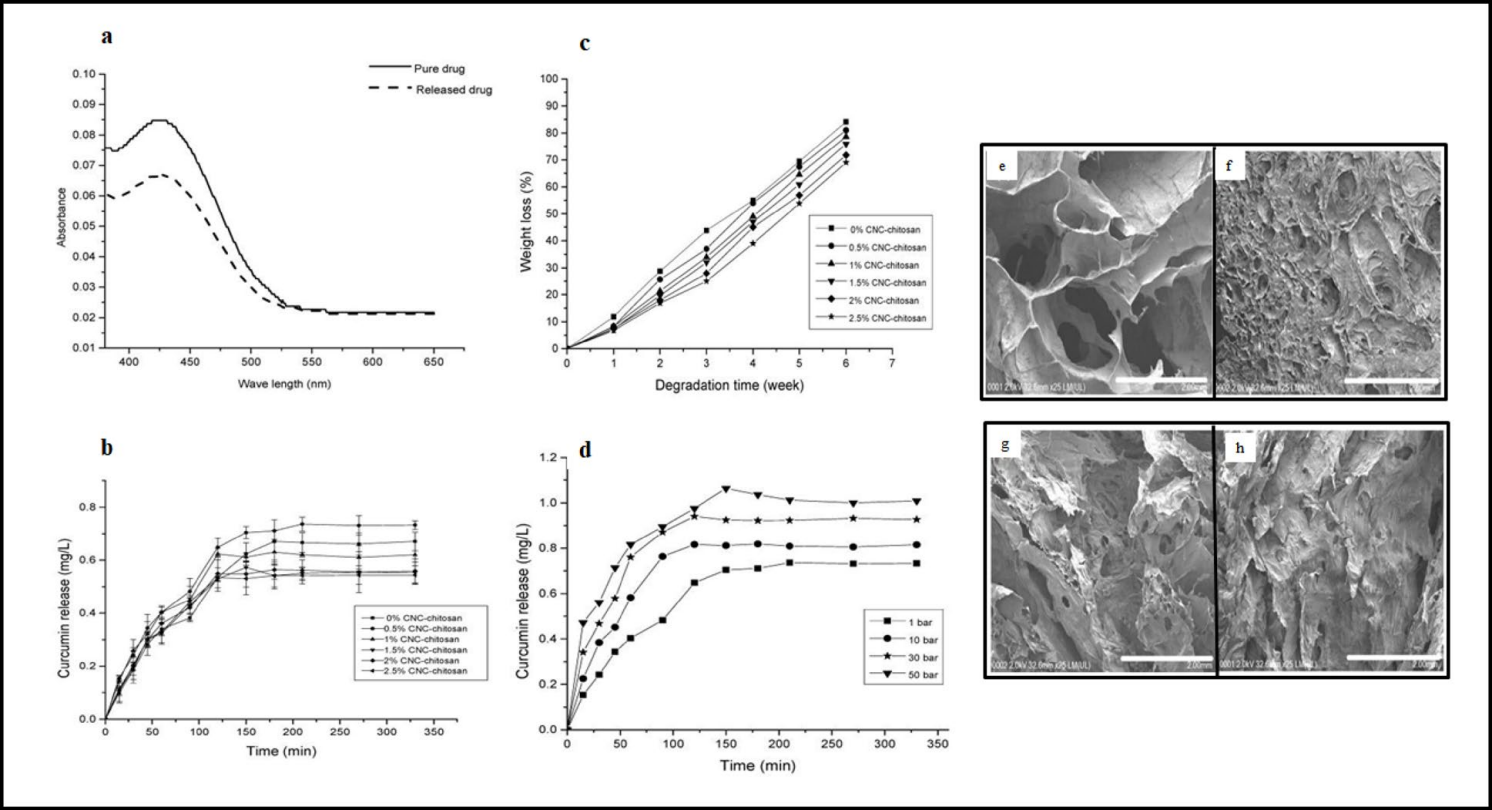
**a** Chemical activity of pure curcumin and curcumin after release,** b** Curcumin release from different types of hydrogels,** c** In vitro degradation study of the hydrogels,** d** Curcumin release from hydrogel formed at different pressure conditions; FESEM images for the cross section of (**e**) gas foamed hydrogel,** f** hydrogel formed at atmospheric condition,** g** gas foamed hydrogel after immersed in SGF, and** h** hydrogel formed at atmospheric condition after immersed in SGF [316]

Researchers have developed a biodegradable, pH-sensitive chitosan hydrogel reinforced with CNC for effective oral delivery of curcumin. Employing CO2 gas foaming to avoid organic solvents, the hydrogel achieved a highly porous and interconnected structure (Fig. 2). The incorporation of CNC significantly enhanced the mechanical strength and compression properties of the hydrogel. It demonstrated excellent swelling ratios and improved drug loading efficiency. In simulated gastric conditions, drug release followed a non-Fickian mechanism, showing increased release over time. FTIR analysis confirmed no chemical interaction between curcumin and the hydrogel matrix, preserving the drug’s activity after release. Release kinetics adhered to the Ritger-Peppas model, ensuring controlled and predictable drug delivery. These findings highlight CNC-reinforced chitosan hydrogels as a promising platform for enhancing curcumin’s bioavailability, enabling efficient absorption in the stomach and upper intestinal tract [316].

Another study investigated a controlled release DDS using chitosan hydrogel loaded with curcumin and selenium nanoparticles (SeNaPs) to treat spinal cord injury (SCI) in rats. The SeNPCur group (Chitosan loaded with both SeNaPs and Cur) demonstrated the lowest nerve tissue damage, minimal inflammation, and the highest astrocyte presence. Histopathological findings highlighted the neuroprotective properties of the chitosan hydrogel loaded with selenium and curcumin, suggesting its potential for SCI treatment [317].

Injectable in situ forming chitosan-based hydrogels were developed using chemical crosslinking of chitosan and genipin, combined with ionic interactions between chitosan and various sodium salts at room temperature. Four formulations were synthesized by incorporating trisodium phosphate (Na3PO4·12H2O), sodium sulfate (Na2SO4), sodium sulfite (Na2SO3), or sodium bicarbonate (NaHCO3). These hydrogels were characterized for their morphology, rheological properties, and biocompatibility, showing low toxicity in cell viability assays. Subcutaneous injections in rats confirmed localized gel formation. Curcumin, a compound with significant pharmaceutical potential but poor bioavailability, was used as a model drug. In vitro release studies revealed sustained curcumin release with an initial burst phase, achieving 3 to 6 times higher cumulative release compared to other gel controls. These findings suggest that the developed hydrogels hold promise as localized DDSs for enhancing curcumin bio-availability [318].

PCL-based scaffolds and nanofibers have gained attention as promising alternatives for prolonged curcumin release and sustained delivery, owing to PCL’s slower degradation in aqueous environments compared to other aliphatic polyesters [313]. A study explored the use of PCL nanocapsules (NCs) to improve curcumin’s bioavailability, antibacterial activity, and immunomodulatory effects. Curcumin-loaded nanocapsules (Cur-NCs) demonstrated remarkable physicochemical stability under simulated gastrointestinal conditions, retaining their integrity post-gastric digestion and enhancing bioaccessibility after intestinal digestion. Antibacterial tests revealed that Curcumin-NCs effectively inhibited Escherichia coli growth while preserving Lactobacillus populations, showcasing selective antibacterial properties. In vitro experiments with ADSCs confirmed that Curcumin-NCs did not compromise cell viability or survival, even in inflammatory environments. Additionally, Cur-NCs exhibited significant immunomodulatory effects by regulating cytokine expression, reducing pro-inflammatory markers like interleukin-1β and tumor necrosis factor-α, while boosting anti-inflammatory cytokines such as IL-10 and transforming growth factor-β. These findings suggest that Cur-NCs hold great promise as a therapeutic strategy to enhance curcumin’s effectiveness, particularly for addressing inflammatory conditions and promoting intestinal health [319].

**Fig. 3:**
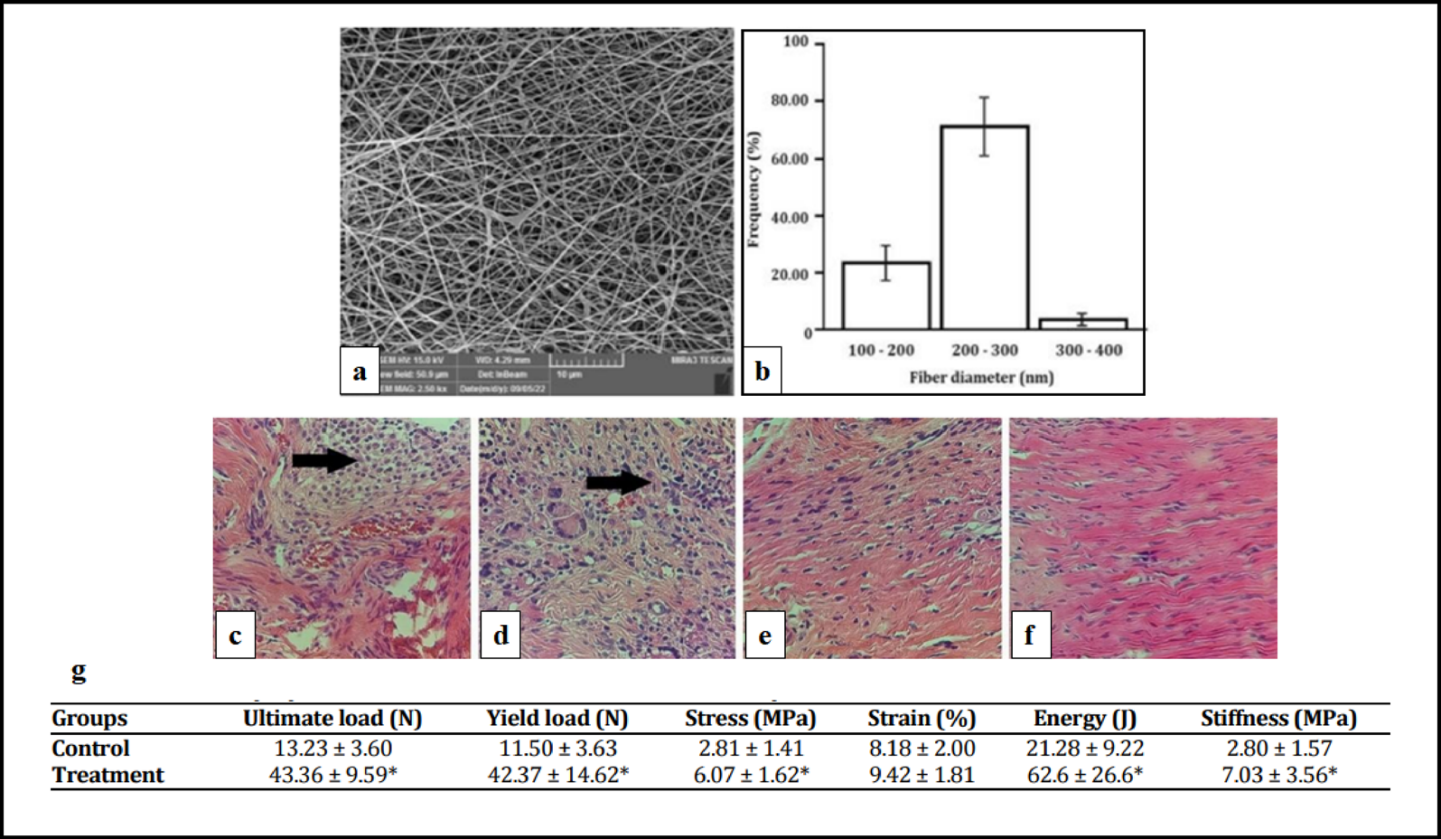
Characterization of curcumin-loaded polycaprolactone scaffold.** a** Scanning electron micrograph of electrospun polycaprolactone nanofibers containing 5.00% curcumin.** b** Diameter distribution of nanofibers in the scaffold. The average diameter of the fibers was 230 ± 50.00 nm (minimum: 127 and maximum: 390 nm); Photomicrographs of the Achilles tendon after 6 weeks. ** c**and** d** Arrows indicate inflammatory cells infiltration in control and treatment groups, respectively. Increased number of inflammatory cells was observed in treatment group versus control group.** e** and** f** Collagen fibers organization in granulation tissue can be seen in control and treatment groups, respectively. In comparison, densely packed and parallel collagen fibers are observed in the treatment group;** g** Mechanical properties of Achilles tendons after 6 weeks [320]

Another study explored the effects of a curcumin-loaded PCL scaffold on Achilles’ tendon healing using a rat tenotomy model. The scaffold, containing 5% curcumin, was fabricated using electrospinning technique and subsequently implanted in rats. Weekly walking track analysis showed significant improvement in the Achilles functional index in scaffold-treated rats from weeks three to six, while the control group exhibited slower progress. Histopathological analysis revealed enhanced neovascularization, improved COL density, and better fibrillar alignment in the treatment group compared to the control group, which showed less organized COL fibers. Mechanical testing demonstrated significantly improved properties (except for strain) in the treated tendons (Fig. 3). These findings suggest that curcumin-loaded PCL scaffolds enhance tendon healing by improving structural and mechanical properties and promoting functional recovery. However, to address biodegradation-induced inflammation observed in the treatment group, incorporating anti-inflammatory measures is recommended [320].

Inflammation remains a significant challenge in tracheal cartilage regeneration, often leading to fibrosis and granulation hyperplasia, which hinder tracheal defect restoration. To address this, a study developed a porous ring-shaped Cur/PCL scaffold by incorporating curcumin, an anti-inflammatory agent, into PCL using the supercritical CO2 foaming method. The Cur/PCL scaffold demonstrated similar porosity to PCL-only scaffolds, while offering sustained release kinetics, stability, and effective anti-inflammatory properties in vitro. Additionally, it exhibited excellent biocompatibility and supported ring-shaped tracheal cartilage regeneration. When implanted subcutaneously into autologous rabbits, the Cur/PCL scaffold significantly enhanced tracheal cartilage regeneration by maintaining the cartilaginous phenotype and suppressing fibrogenesis and granulation hyperplasia. Furthermore, following orthotopic transplantation, the engineered cartilage in the cur/PCL group improved survival rates in experimental rabbits. This study highlights a promising strategy for developing anti-inflammatory scaffolds to promote cartilage regeneration and repair tracheal cartilage defects in vivo [321].

Additionally, PCL microspheres optimized through response surface methodology have demonstrated efficient curcumin encapsulation, further highlighting their potential as a viable option for sustained drug delivery applications [313].

A groundbreaking material that combines the biocompatibility and antimicrobial properties of chitosan with the flexibility, durability, and biodegradability of PCL has been developed, offering a promising solution for cartilage repair. A recent study introduced a novel chitosan-based injectable hydrogel designed to overcome challenges in cartilage repair, including bio-compatibility, mechanical stability, and efficient drug delivery. This thermo-sensitive hydrogel, composed of Chitosan/β-glycerophosphate (CH/β-GP) reinforced with bacterial cellulose (BC) nanofibers and loaded with curcumin-embedded polycaprolactone microspheres (PCL MS), exhibited promising features for cartilage regeneration. The hydrogel demonstrated optimal porosity, pore size, and compressive modulus, alongside antibacterial activity, a controlled curcumin release profile, and degradation rate aligned with cartilage repair timelines. Anti-bacterial tests confirmed its efficacy against *S. aureus* and *E. coli*, while bio-compatibility assays revealed high fibroblast viability over a 7-day period. In vivo studies on rabbit models showed significant improvements in cartilage regeneration, including enhanced mobility, accelerated cartilage formation, and reduced defect boundaries. Histological analysis further highlighted increased chondrocyte density compared to controls. This innovative hydrogel leverages the structural support of BC, the controlled drug release of PCL MS, and the excellent biocompatibility of CS hydrogels, offering a promising alternative to traditional cartilage repair therapies [313].

A novel DDS for OA treatment, called dCOL2-CM-Cur-PNPs, has been developed to address the limitations of traditional therapies, such as rapid clearance and low biocompatibility. This system integrates induced pluripotent stem cell-derived mesenchymal stem cell membranes (iMSC-CMs), curcumin-loaded PLGA nanoparticles (Cur-PNPs), and damaged type II collagen (dCOL2)-targeting phospholipids. The iMSC-CM coating enhances curcumin’s sustained release and cellular uptake by OA-induced chondrocytes, restoring their chondrogenic properties while inhibiting pro-inflammatory M1 macrophages and promoting anti-inflammatory M2 macrophages. The dCOL2-targeting phospholipids ensure precise binding to OA cartilage, validated through in vitro and in vivo studies. This DDS demonstrated significant efficacy in alleviating OA progression in a DMM rat model by simultaneously addressing inflammation and promoting cartilage regeneration. By overcoming conventional limitations, the dCOL2-CM-Cur-PNPs system shows immense potential as a next-generation platform for targeted OA therapy [322].

A recent study introduced an innovative curcumin delivery system utilizing PEG-GelMA (PGMs) hydrogel microgels to enhance anti-inflammatory and regenerative effects for treating cartilage defects.

Created through a microfluidic system utilizing light-induced gelation of GelMA, these curcumin-loaded PGMs were shown to support mesenchymal stem cell proliferation and chondrogenic differentiation at low curcumin dosages in vitro. Additionally, the PGMs effectively reduced inflammatory responses in chondrocytes exposed to IL-1β stimulation. In vivo studies demonstrated that the injectable PGMs significantly improved the repair of large cartilage injuries. This dual-action therapeutic approach not only protects cartilage under inflammatory conditions but also promotes efficient regeneration, offering an advanced strategy for addressing extensive cartilage damage [323].

**Fig. 4:**
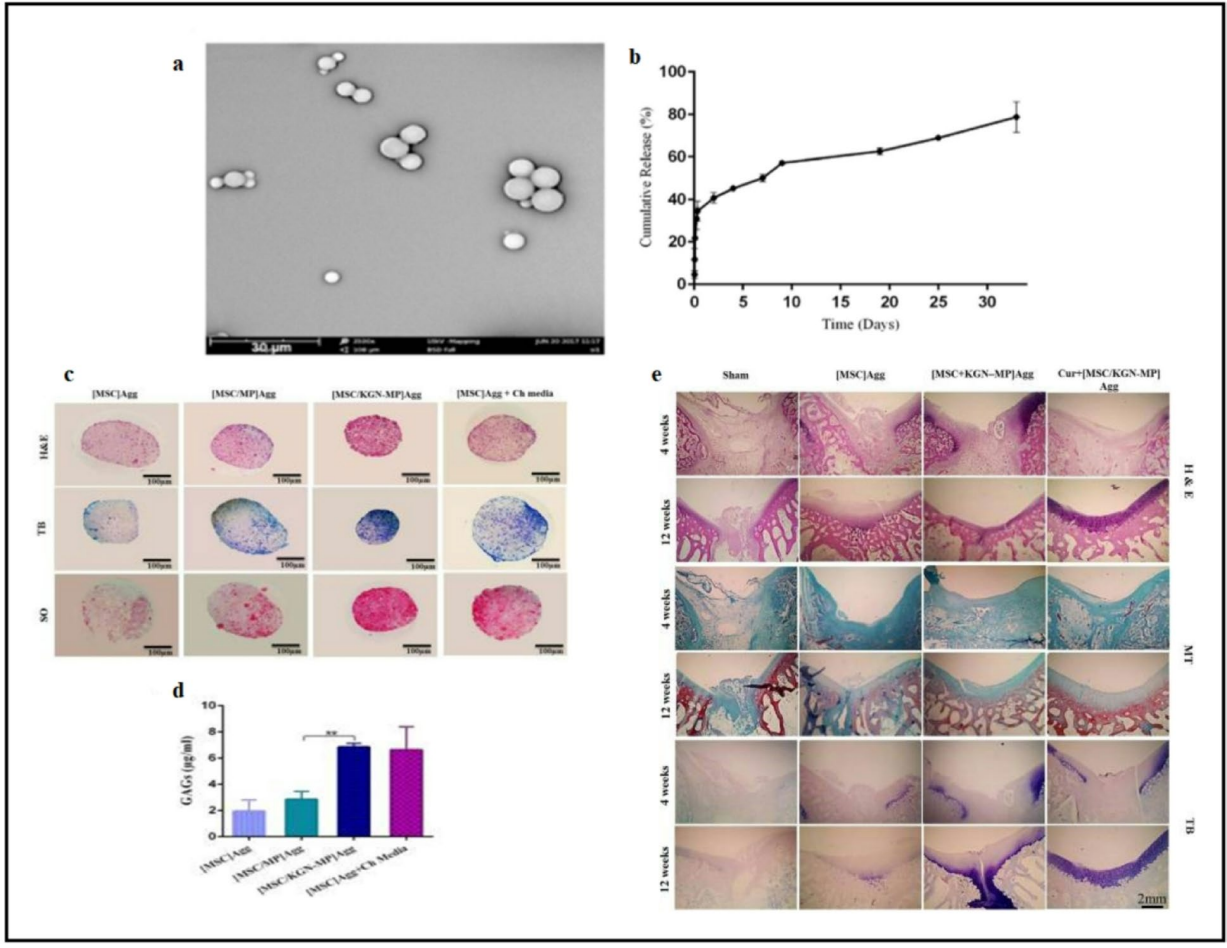
**a** Scanning electron microscopy images of MPs show their smooth surface morphologies. The mean diameter of the particles was 11 ± 5.5 μm.** b** KGN release profile from MPs for 32 days; Cartilage matrix production in the presence of KGN-loaded MPs in MSC aggregates,** c** Histological evidence of chondrogenesis in the MSC Agg, MSC/MP Agg, MSC/KGN-MP Agg and MSC Agg + Ch media groups after 14 days,** d** Sulfated GAG content in the aggregate groups,** e** Histological findings of rabbit osteochondral defect repair at 4 and 12 weeks after treatment. Hematoxylin and eosin (H&E), Masson’s trichrome (MT), and toluidine blue (TB) staining at 4 and 12 weeks after implantation [325]

Researchers have developed an advanced strategy for cartilage repair by engineering chondrogenic microtissues using MSC aggregates integrated with kartogenin-releasing PLGA microparticles (KGN-MP). These microtissues demonstrated enhanced chondrogenic potential, confirmed through RNA-level expression of cartilage markers and uniform GAGs production, visualized via toluidine blue and safranin O staining (Fig. 4). To address inflammation at the defect site, the microtissues were delivered using an injectable GelMA hydrogel containing curcumin, known for its anti-inflammatory properties. Real-time RT-PCR analysis showed that KGN stimulated cartilage differentiation in MSCs, while curcumin effectively reduced hypertrophy marker expression. In vivo experiments revealed successful cartilage regeneration with structural and functional characteristics closely resembling natural hyaline cartilage, as validated by observational and histological analyses. This innovative combination of chondrogenic cell aggregates and curcumin-loaded hydrogel presents a promising and effective approach for advanced cartilage repair therapies [324].

A study developed an in situ forming gel for the prolonged delivery of curcumin and piperine to treat inflammatory and degenerative joint diseases such as osteoarthritis and rheumatoid arthritis. The formulation uses glyceryl monooleate, phosphatidylcholine, and ethanol to create a liquid injectable that transforms into a semisolid upon contact with biological fluids. Curcumin and piperine were incorporated either individually or together into the gels. In vitro studies demonstrated sustained drug release over 96 h, with faster release observed when both drugs were combined. Mechanistic analysis revealed that drug release was governed by diffusion and swelling/erosion of the lipid matrix. Ex vivo testing on human skin validated the gel’s compatibility for subcutaneous injection, with piperine significantly enhancing curcumin release by twofold compared to curcumin-only formulations. This innovative gel system shows promise for localized and long-lasting drug delivery in the treatment of joint diseases [326].

A study explored the protective effects of curcumin-loaded poly lactic-co-glycolic acid nanoparticles (nanocurcumin) in a rat model of mono-iodoacetate-induced osteoarthritis. Fourteen days after OA induction, curcumin and nanocurcumin were administered orally at a dose of 200 mg/kg for two weeks. Results showed that both curcumin and nanocurcumin significantly improved cellularity and matrix staining in the AC compared to the mono-iodoacetate group. Notably, the effects were more pronounced in the nanocurcumin-treated group. These findings indicate that nanocurcumin effectively prevents structural damage to articular cartilage in this OA model, suggesting its potential as a therapeutic agent [327].

Researchers explored the synergistic effects of curcumin supplementation and BMSCs on cartilage repair and OA treatment. In vitro experiments demonstrated that while curcumin alone did not enhance the viability of primary articular chondrocytes, it significantly promoted their proliferation and migration when co-cultured with BMSCs. Additionally, curcumin increased the expression of anabolic genes in chondrocytes at both mRNA and protein levels. In vivo, a rat OA model induced by mono iodoacetic acid showed that intra-articular injections of allogeneic BMSCs combined with curcumin supplementation greatly improved AC repair and slowed OA progression, as confirmed by histological staining and scoring methods. These findings highlight that curcumin enhances BMSC-mediated therapeutic effects, including chondrocyte proliferation, migration, and ECM production. This combined approach holds promise for clinical applications in cartilage repair and OA management [328].

Another study explored the effects of curcumin-loaded PLGA nanoparticles on knee joints in a rabbit model of OA induced by anterior cruciate ligament transection (ACLT). Fifteen rabbits were divided into three groups: curcumin-treated, curcumin-containing PLGA nanoparticle-treated, and vehicle-treated. Weekly intra-articular injections were administered for six weeks. Cartilage damage was evaluated using the modified Mankin score, and gene and protein expressions of MMP-1, MMP-13, and TIMP-1 were analyzed via qPCR and western blotting. Results showed that curcumin-treated joints exhibited reduced cartilage damage compared to vehicle-treated joints, with further improvement observed in the curcumin-containing PLGA nanoparticle group. Additionally, curcumin treatment downregulated MMP-1 and MMP-13 while upregulating TIMP-1, with these effects being significantly amplified by the PLGA nanoparticle delivery system. These findings suggest that PLGA nanoparticles enhance the chondro-protective effects of curcumin, offering a promising approach for OA treatment in the future [329].

**Fig. 5:**
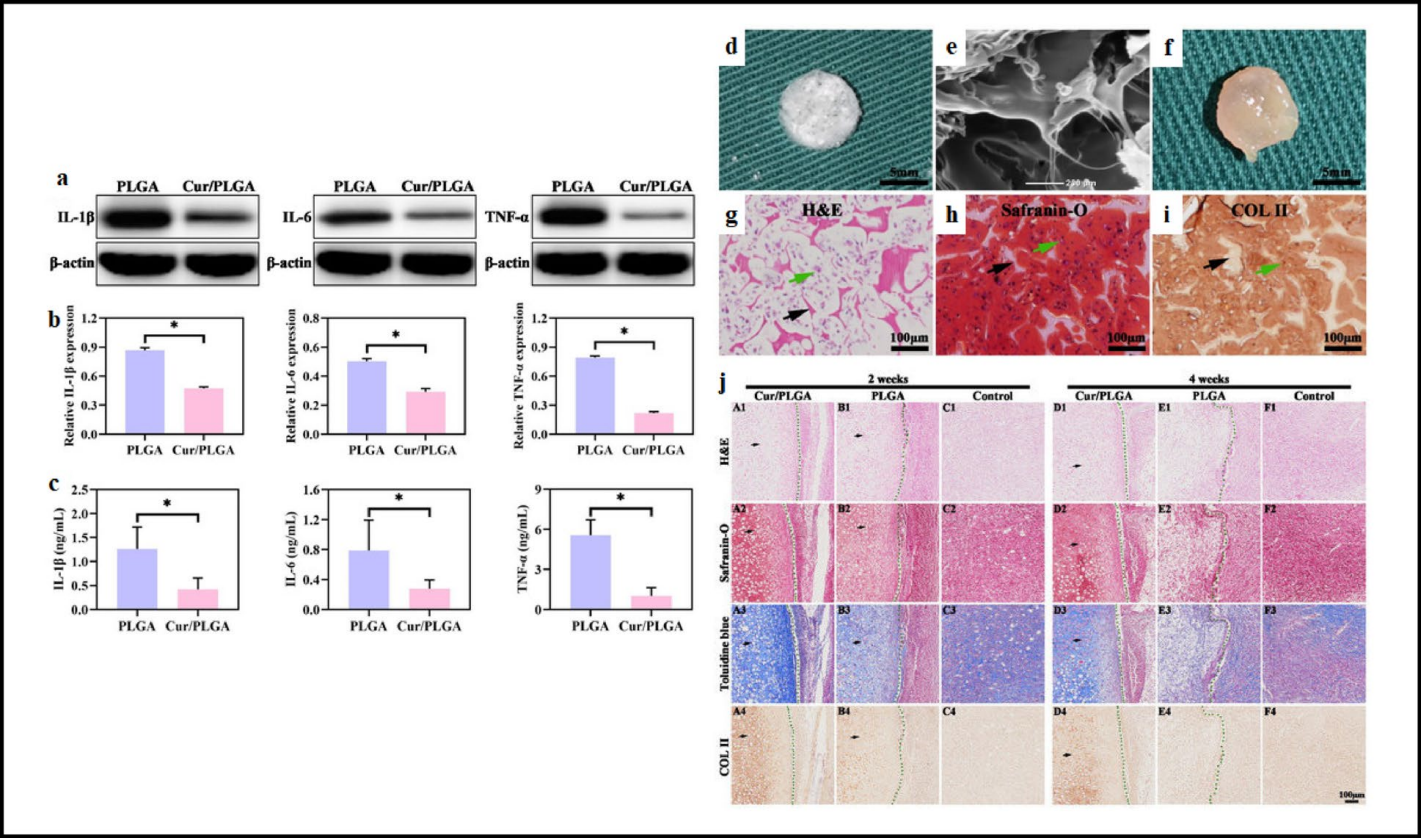
In vitro anti-inflammatory activities of the Cur/PLGA nanofibrous membrane,** a** The expression levels of IL-1β, IL-6, and TNF-α in the PLGA and Cur/PLGA nanofibrous membranes incubated with RAW264.7 macrophages for 24 h were determined using Western blotting,** b** The quantification of the expression levels of IL-1β, IL-6, and TNF-α in the PLGA and Cur/PLGA groups from the blots,** c** The expression levels of IL-1β, IL-6, and TNF-α in the PLGA and Cur/PLGA nanofibrous membranes incubated with RAW264.7 macrophages for 24 h were also examined using an enzyme-linked immunosorbent assay;** d** Images and (**e**) scanning electron micrographs of a porous PLGA scaffold, (**f**) Images, (**g**) hematoxylin-and-eosin staining,(** h**) safranin-O staining, and (**i**) COL II immunohistochemical staining of the PLGA scaffold seeded with chondrocytes for 3 weeks;** j** Histological evaluation of the nanofibrous-membrane-packaged cartilage tissue at weeks 2 and 4 after subcutaneous implantation in rats [330]

A recent study introduced a novel approach by incorporating curcumin, a known anti-inflammatory agent, into PLGA to create a Cur/PLGA nanofibrous membrane with nanoscale pores and anti-inflammatory properties. Analytical techniques confirmed the successful integration of Cur, which demonstrated sustained release and enhanced stability in vitro. The membrane significantly reduced inflammatory cytokines such as IL-1β, IL-6, and TNF-α, as evidenced by Western blotting and ELISA (Fig. 5). Engineered cartilage, developed by seeding chondrocytes into a porous PLGA scaffold, was wrapped with the Cur/PLGA membrane and implanted into rats. Results showed that the Cur/PLGA membrane created an immunosuppressive environment, preserving the cartilage and mitigating inflammation compared to control groups. By effectively protecting engineered cartilage from immune rejection, this strategy holds promise for advancing the clinical application of tissue-engineered cartilage in repairing cartilage defects [330].

A study investigated the therapeutic effects of curcumin nanoparticles combined with dexmedetomidine (DEX) on cartilage injury in a posttraumatic osteoarthritis (PTOA) rat model. Rats treated with curcumin nanoparticles and DEX showed significant improvement in cartilage pathology, with a lower Mankin’s score compared to those treated with DEX alone. The combination therapy also resulted in reduced levels of inflammatory markers IL-1β, TNF-α, and MMP-3 in joint cavity fluid, with the lowest levels observed in the curcumin nanoparticles + DEX group. Mechanistically, the combination inhibited the NF-κB pathway, reducing inflammation and pain while protecting cartilage from degeneration. DEX further contributed by decreasing sensitivity to adrenaline and suppressing the peripheral release of inflammatory factors. Together, these findings suggest that curcumin nanoparticles combined with DEX offer synergistic anti-inflammatory and analgesic effects, helping to delay PTOA progression and preserve joint health [14].

Researchers have developed a pH-responsive MOFs, MIL-101-NH2, for the co-delivery of curcumin, an anti-inflammatory drug, and small interfering RNA (siRNA) targeting hypoxia-inducible factor (HIF-2α). Curcumin and siRNA were incorporated into MIL-101-NH2 via encapsulation and surface coordination. In vitro studies demonstrated that MIL-101-NH2 safeguarded siRNA from nuclease degradation and facilitated lysosomal escape. Under acidic conditions mimicking the OA microenvironment, the MOF gradually disintegrated, releasing curcumin to reduce pro-inflammatory cytokines and siRNA to silence HIF-2α mRNA through gene therapy. This dual-action approach showed synergistic therapeutic effects by simultaneously mitigating inflammation and cartilage degeneration in OA. Both in vitro and in vivo results highlighted the promising potential of this hybrid material for OA treatment, offering an innovative strategy that leverages MOFs as effective delivery platforms for combined drug and gene therapies [14].

Another study explored the development of three-dimensional printed scaffolds combining PCL and solubilized decellularized cartilaginous ECM derived from bovine femur. These scaffolds were crosslinked using either N-(3-dimethylaminopropyl)-N-ethylcarbodiimide hydrochloride/N-hydroxysuccinimide (EDC/NHS) or genipin, and further loaded with curcumin for enhanced functionality. Human chondrocyte cells were cultured on these scaffolds to assess various parameters such as cell viability, attachment, and histological outcomes. The study revealed that genipin-crosslinked scaffolds exhibited superior biocompatibility, with lower porosity, reduced water absorption, slower degradation rates, and enhanced cell proliferation and attachment compared to EDC/NHS-crosslinked scaffolds. Furthermore, genipin-crosslinked curcumin-loaded scaffolds demonstrated higher cell viability but lower antibacterial activity relative to their EDC/NHS counterparts, suggesting potential cytotoxic effects induced by EDC/NHS crosslinking. These findings highlight the promise of PCL-dECM genipin-crosslinked curcumin-loaded scaffolds as biocompatible and effective tools for CTE applications [331].

Researchers have introduced a groundbreaking scaffold that integrates curcumin and silk fibroin, aiming to revolutionize cartilage repair techniques. Using the salt leaching method, they created scaffolds with varying curcumin concentrations (0.5 mg/ml, 1 mg/ml, 2 mg/ml) to achieve optimal pore sizes and mechanical properties. Among these, the scaffold with 1 mg/ml curcumin demonstrated the best results, supporting chondrocyte viability and ECM formation more effectively than the other groups. Furthermore, the curcumin/silk scaffold showed excellent biocompatibility and facilitated cartilage repair in vivo, as confirmed by histological analysis. These findings underscore the potential of this composite scaffold as a promising solution in CTE and repair applications [332].

Another recent study highlighted the development of curcumin-loaded biodegradable PU scaffolds, modified with gelatin, for potential use in cartilage regeneration. Utilizing 3D printing technology, the scaffolds were created using a solution of PCL triol and hexamethylene diisocyanate (HMDI) in 2,2,2-trifluoroethanol, printed in a dimethyl sulfoxide phase with or without gelatin. By varying the weight ratio of HMDI to PCL triol (3, 5, and 7), researchers observed that higher HMDI ratios improved mechanical strength while reducing the biodegradation rate. Incorporating gelatin further enhanced mechanical properties, biodegradation rate, and curcumin release due to its surface cross-linking, nano-porous structure, and hydrophilic properties. In vitro testing demonstrated that the released curcumin promoted chondrocyte proliferation and differentiation. These findings suggest that the 3D-printed biodegradable PU scaffold, enhanced with gelatin, is a promising candidate for cartilage regeneration applications [333].

A recent study investigated the anti-inflammatory effects of curcumin on OA-induced knee damage in rats. Results showed that curcumin-treated OA group had increased chondrocyte numbers in surface and middle layers, reduced middle and deep layer thickness, and elevated Col2 expression across all layers compared to OA group. SOX-5 expression also increased in surface and deep layers of treated group. Importantly, curcumin-treated OA group showed no increase in cartilage thickness or MMP-8 and MMP-13 expression, highlighting curcumin’s protective effects. These findings suggest that curcumin may mitigate cartilage degradation and promote cartilage health by enhancing IHH, Col2, and SOX-5 expression while preserving chondrocytes [334].

Researchers have explored the potential of combination therapy involving curcumin and other compounds like probucol, chondroitin sulfate, and resveratrol to enhance therapeutic outcomes in cartilage injury and OA. In an experimental knee osteoarthritis (KOA) model induced by monosodium iodoacetate (MIA) in rats, the combination of Cur and CS significantly downregulated serum levels of IL-1β, SOD, and MMP-3 compared to the model group. Additionally, improved cartilage repair with higher collagen II content was observed in the combined therapy group compared to groups treated with either curcumin or CS alone [335]. Similarly, curcumin and resveratrol demonstrated synergistic effects in protecting human articular chondrocytes by inhibiting IL-1β-induced-κB-mediated inflammation and apoptosis [336]. Furthermore, the combination of curcumin and probucol, known for their cholesterol-lowering properties and ability to protect chondrocytes from inflammatory stress, showed protective effects by reducing apoptosis and mitigating TNF-α-induced cartilage degradation in both in vitro and in vivo studies, thereby alleviating OA severity [337].

Researchers have developed an innovative nanocomposite hydrogel scaffold aimed at treating intervertebral disc degeneration (IDD), integrating curcumin-loaded solid lipid nanoparticles (Cur -SLNs) into a GelMA hydrogel. To create the Cur-SLNs, curcumin was dissolved in a 1:1 mixture of acetone and chloroform and combined with stearic acid, cholesterol, and lecithin to form an oil phase, which was then emulsified with an aqueous solution containing Poloxamer and Tween 80. This emulsion underwent low-temperature solidification to produce the Cur-SLNs, which were subsequently encapsulated within the GelMA hydrogel. The resulting composite scaffold demonstrated significant therapeutic benefits in vivo, including enhanced type II collagen repair, increased aggrecan expression, and reduced levels of inflammatory markers such as TNF-α and IL-6. Compared to GelMA alone or GelMA with curcumin, the Cur-SLNs/GelMA scaffold exhibited superior regenerative effects, highlighting its potential for disc regeneration. Despite these promising results, further research is needed to comprehensively validate its efficacy and explore its clinical applications [338].

A study explored the development of scaffolds composed of multi-walled carbon nanotubes (MWCNTs), COL, and curcumin using a freeze-drying technique. The objective was to investigate how varying concentrations of MWCNTs and curcumin influenced the physical, chemical, and biological characteristics of collagen-based scaffolds through both in vitro and in vivo analyses. COL was dissolved in 0.02 normal acetic acid, while curcumin was integrated into the scaffold without the use of an additional solvent. The results highlighted the therapeutic and anti-inflammatory properties of curcumin, demonstrating its biocompatibility when 10% Cur was incorporated into a 1% COL-MWCNT scaffold. This specific formulation not only reduced inflammation in surrounding tissues but also enhanced the mechanical and physical properties of the scaffold [60].

Another study highlights the potent antibacterial properties of tetrahydrocurcumin (THC)-based scaffolds, which leverage the mechanisms of curcumin and its metabolites. These compounds effectively disrupt bacterial cell membranes by interacting with the lipid bilayer, increasing permeability, and inducing cell death. Additionally, they inhibit bacterial enzymes and proteins, thereby interfering with essential metabolic pathways, while simultaneously generating ROS that lead to oxidative stress and cellular damage. The scaffolds, designed for controlled THC release, demonstrated significant antibacterial efficacy against *Staphylococcus aureus*. Fabricated using fish gelatin and lactose dissolved in distilled water, with glycerol and THC incorporated, these scaffolds also exhibited enhanced mechanical properties, remarkable water uptake capacity exceeding 800%, and a controlled release of 80% THC within three days. These attributes make them highly promising for applications in managing postoperative inflammation and providing antimicrobial protection [339].

Bone marrow stem cell (BMSC)-regenerated cartilage (BRC) holds great potential for repairing cartilage defects clinically, but its tendency to ossify due to vascular invasion and inflammation in subcutaneous environments poses a challenge. To overcome this, researchers developed a core–shell structured nanomembrane (KGN/Gel@Cur/PLCL) using coaxial electrospinning technology. The shell layer, containing curcumin and poly(L-lactide-co-caprolactone) (PLCL), provides immunomodulatory and anti-angiogenic effects, while the core layer, comprising KGN and Gel, promotes chondrogenesis. When encapsulating BRC derived from goat BMSCs and implanted subcutaneously into autologous goats, the nanomembrane acted as a physical barrier, preventing vascular invasion and inflammation through Cur release. Simultaneously, KGN release supported cartilage maturation and sustained the chondrogenic phenotype of the BRC. These combined effects significantly reduced ossification and preserved chondrogenic stability in the subcutaneous niche. This innovative approach demonstrates the potential of sequential immune and vascular isolation followed by chondrogenic induction to enhance the clinical feasibility of stem cell-based cartilage repair in large animals [340].

A study investigated the potential of Gel /Cur nanofibrous scaffolds for cartilage tissue engineering, revealing promising results. These scaffolds were fabricated through a multi-step process involving the use of isopropyl alcohol to dissolve Gel, hexafluoroisopropanol (HFIP) for dissolving PLA, 95% ethanol for EDC/NHS crosslinking, and ethanol with 5% glacial acetic acid for curcumin dissolution. After fabrication, the nanofiber membranes were rinsed with deionized water and subsequently freeze-dried. The experimental group demonstrated a significant reduction in angiogenesis compared to the control group, underscoring the potential of these scaffolds as an effective solution for cartilage repair applications [341]. In conclusion, based on the various studies conducted on curcumin-loaded scaffolds, it can be inferred that curcumin, as an anti-angiogenic agent, effectively inhibits angiogenesis and prevents the formation of bone tissue in the regenerated cartilage. Utilizing a low dose of curcumin (around 20 to 30 micromolar) may serve as a promising alternative for cartilage repair and CTE applications [342].

**Table 2:** Curcumin-based scaffolds and drug delivery systems for cartilage regeneration

Scaffold/DDS type	Key advantages/functionalities	Main outcomes	Study model	Refs.
Gel/Cur Nanofibers	Low-dose Curcumin (20–30 µM) delivery	Significant Angiogenesis reduction vs. controls	In vitro: Anti-angiogenic assays	[115]
CH Hydrogel + MMT/Ag NPs	pH-sensitive release; oxygen plasma enhances antibacterial/drug release	Controlled curcumin release; strong antibacterial effects	In vitro: pH-release assays	[205]
CH + CNC Hydrogel	Porous structure; High Mechanical Strength	Sustained release in simulated gastric conditions	In vitro: Simulated GI conditions	[206]
CH + SeNPs Hydrogel	Neuroprotective/Anti-inflammatory	Reduced nerve tissue damage in spinal cord injury models	In vivo: Rat SCI model	[207]
Injectable CH + Genipin	In situ gelation; biocompatibility	Prolonged curcumin release (3–6× higher than controls)	In vitro/In vivo: Rat subcutaneous	[208]
PCL Nanocapsules	Enhanced bioavailability; immunomodulatory	Selective antibacterial action (against E. coli); reduced pro-inflammatory cytokines	In vitro: ADSCs	[209]
THC	Membrane disruption, enzyme inhibition, ROS generation	80% THC release in 3 days; 800% water uptake; effective against S. aureus	In vitro: Antibacterial assays	[225]
Electrospun PCL Scaffold	Slow degradation; mechanical support	Improved tendon healing (collagen alignment, neovascularization)	In vivo: Rat tendon injury	[210]
PCL Supercritical Foaming Scaffold	Anti-inflammatory; sustained release	Suppressed tracheal fibrosis; enhanced cartilage phenotype in rabbits	In vitro/In vivo: Rabbit trachea	[211]
CS/β-GP + PCL MS	Thermosensitive; antibacterial	Accelerated cartilage formation in rabbits; high chondrocyte density	In vitro/In vivo: Rabbit cartilage	[203]
PLGA NPs (dCOL2-CM-Cur-PNPs)	Targeted delivery (OA cartilage)	Reduced cartilage damage; downregulated MMPs in OA models	In vitro/In vivo: OA rat model	[212]
PEG-GelMA	Microfluidic fabrication; anti-inflammatory	Enhanced chondrogenesis; reduced IL-1β-induced inflammation	In vitro: IL-1β-chondrocytes; In vivo: Cartilage defect	[213]
KGN-MP + Cur/GelMA	Chondrogenic + anti-inflammatory	Hyaline-like cartilage regeneration; reduced hypertrophy	In vitro: MSC chondrogenesis	[214]
In Situ Forming Lipid Gel	Solvent-free transformation; dual drug loading	96 h sustained release; piperine enhanced curcumin release 2×	In vitro/Ex vivo: Human skin permeation	[215]
PLGA Nanoparticles	Improved bioavailability	Prevented articular cartilage damage; more effective than free curcumin	In vivo: MIA-induced OA rat model	[216]
Cur + BMSCs	Enhanced MSC therapeutic effects	Improved chondrocyte proliferation/migration; slowed OA progression	In vitro/In vivo: MIA-induced OA rat model	[217]
Cur-PLGA NPs (ACLT Model)	Enhanced chondroprotection	Downregulated MMP-1/13; upregulated TIMP-1; reduced cartilage damage	In vitro/In vivo: ACLT-induced OA rabbit model	[218]
Cur/PLGA Nanofibrous Membrane	Anti-inflammatory barrier	Reduced IL-1β/IL-6/TNF-α; protected engineered cartilage from immune rejection	In vitro/In vivo: Rat cartilage defect model	[219]
Curcumin NPs + DEX	NF-κB pathway inhibition	Synergistic anti-inflammatory/analgesic effects; reduced IL-1β/TNF-α/MMP-3	In vivo: PTOA rat model	[15]
SF	Salt-leached porosity	Improved chondrocyte viability and cartilage repair in vivo	In vitro/In vivo: Rabbit defect	[221]
3D-Printed PU + Gel	Tunable mechanical strength (via HMDI/PCL ratio)	Promoted chondrocyte proliferation/differentiation	In vitro: Chondrocyte culture	[49]
MWCNT-CoL-Cur	Anti-inflammatory	Reduced inflammation; improved mechanical properties	In vitro: Biocompatibility	[42]
Core–Shell Nanomembrane	Anti-angiogenic/immunomodulatory	Prevented ossification in BMSC-regenerated cartilage	In vivo: Goat subcutaneous	[51]
Cur-SLNs/GelMA	Enhanced type II collagen/aggrecan	Reduced TNF-α/IL-6; improved disc regeneration	In vivo: Rat disc degeneration	[224]
Cur/ CS	Synergistic anti-inflammatory	Higher collagen II content in OA cartilage	In vivo: MIA-OA rat	[223]
Cur/ Resveratrol	Inhibits NF-κB-mediated inflammation	Protected chondrocytes from IL-1β-induced damage	In vitro: IL-1β-chondrocytes	[198]
Cur/ Probucol	Cholesterol-lowering + anti-apoptotic	Reduced TNF-α-induced cartilage degradation	In vitro/In vivo: OA model	[200]

#### Scaffold fabrication methods for cartilage tissue engineering

Tissue engineering relies on various scaffold fabrication techniques, each tailored to specific biomaterials, textures, and applications. Common methods include freeze-drying, which creates porous structures ideal for cell growth, and electrospinning, known for producing nanofiber scaffolds that mimic the ECM. Solvent casting and particulate leaching offer control over porosity but may involve toxic solvents. Gas foaming avoids solvents and generates interconnected pores but can lack structural uniformity. Sol-gel processes are effective for ceramic-based scaffolds, while self-assembling techniques leverage molecular interactions for precise architectures. Additive manufacturing, or 3D printing, provides unmatched customization and complexity, making it a versatile choice for personalized constructs. Each method has unique benefits and limitations depending on the intended application [232].

##### Freeze-drying

Freeze-drying is a prominent technique in tissue engineering for fabricating porous scaffolds, achieved by removing the solvent from a frozen solution under vacuum to create an anhydrous 3D structure [239]. This straightforward dehydration process allows for the production of scaffolds in various engineered shapes. Although freeze-dried scaffolds typically exhibit low mechanical strength, cross-linking can be applied to improve their durability and resistance to enzymatic degradation [89]. These scaffolds are distinguished by their high porosity and interconnected uneven pores, which facilitate superior cell and tissue infiltration. Furthermore, their high surface-to-mass ratio enhances protein adsorption compared to solid materials. Owing to these beneficial properties, freeze-dried scaffolds are widely employed in diverse tissue engineering applications, including wound healing, spinal cord repair, nerve regeneration, cartilage repair, and tendon repair [232, 343–345].

##### Phase-separation

Phase-separation is a fabrication method used to create 3D nanofibrous structures that closely resemble the dimensions of COL fibrils in the ECM (50–500 nm) [346]. This technique relies on the physical incompatibility of polymers, causing them to separate into two phases for nanofiber production. It offers the advantage of creating scaffolds with both nano- and macro-scale architectures tailored to specific anatomical shapes. While phase-separation is relatively simple and requires minimal instrumentation, it is limited to certain polymer-solvent combinations. Additionally, controlling fiber dimensions is challenging, and the resulting fibers have mechanical properties unsuitable for load-bearing applications due to their highly porous nature. Key parameters influencing the process include the type and concentration of the polymer, the choice of solvent, and thermal treatment [347].

##### Electrospinning

Electrospinning is a prominent technique in tissue engineering for fabricating nanofibrous scaffolds with tunable properties such as fiber diameter, porosity, and surface morphology. This cost-effective method produces nonwoven mats with high interconnected porosity, though precise control over pore size remains challenging [348, 349]. Composite electrospun scaffolds combine the biological benefits of natural polymers with the mechanical strength of synthetic materials. Electrospun scaffolds, made from both natural and synthetic polymers like COL [350], silk fibroin [351], PCL, PLA, and PLGA [307], are widely used in applications such as cartilage, tendon-to-bone, and bone tissue engineering [352]. Additionally, ceramic materials like hydroxyapatite [353] and TiO2 [354] have also been electrospun for bone repair, demonstrating enhanced osteoblast differentiation. Parameters like polymer solution properties, electric potential, and collector design influence fiber characteristics such as alignment and crimping, which can mimic the ECM and guide cell behavior. Advancements like coaxial electrospinning enable controlled drug release, while modifications such as low-temperature or needleless electrospinning aim to overcome limitations like poor cell penetration in 2D matrices. Ceramic materials like hydroxyapatite [353] and TiO2 [354] have also been electrospun for bone repair, demonstrating enhanced osteoblast differentiation. Electrospinning is a versatile technique influenced by factors like polymer molecular weight, solution properties, electric potential, and the distance between the capillary and collector. Despite its advantages, it faces challenges such as jet instability, toxic solvents, and the production of 2D matrices with small pores that limit cell penetration and vascular ingrowth [355]. Advancements in processing conditions have been explored to overcome these limitations. Coaxial electrospinning allows for the creation of core-shell nanofibers, enabling controlled drug or biomolecule release. Efforts to develop 3D macroporous scaffolds include low-temperature electrospinning, needleless systems using disc spinnerets, and alternative collector designs like parallel plates or screws. Techniques such as introducing micrometer-sized fibers or inert particle spacers (e.g., salts, PEO, or gas) have also been employed. Additionally, polyelectrolyte solutions can extend fibers outward due to charge-induced repulsion. Post-treatments like photo-masking or stacking layered mats further enhance scaffold properties. These innovations aim to maximize the potential of electrospinning for biomedical applications [356].

##### 3D Bioprinting

3D bioprinting has emerged as a revolutionary technique in tissue engineering, offering unparalleled precision and flexibility in creating complex biological structures. By integrating the principles of biomimetic tissue reconstruction with advanced 3D printing technologies, this method has transformed the design and fabrication of tissue engineering scaffolds. Utilizing biomaterials, bioactivators, and even living cells, 3D bioprinting replicates structures that closely resemble natural tissues through a layer-by-layer additive manufacturing process [14, 357]. The primary bioprinting techniques include inkjet, laser-assisted, and extrusion-based methods, each with distinct mechanisms and advantages. Inkjet bioprinting involves dispensing bio-ink droplets composed of hydrogel prepolymers and embedded cells via printer heads actuated by piezoelectric or thermal systems. Laser-assisted bioprinting uses laser pulses to transfer bio-ink from a donor slide to a receiver slide by vaporizing a metal layer beneath the hydrogel. Extrusion-based bioprinting, one of the most widely utilized approaches, employs bio-inks loaded into syringes that are pneumatically or mechanically extruded to form 3D constructs. The selection of bioprinting methods and materials plays a critical role in determining the properties of the resulting scaffolds, underscoring the importance of tailoring these parameters to meet specific tissue engineering requirements [358, 359].

## Curcumin: a novel herbal agent for advancing cartilage tissue engineering

### Molecular mechanisms and therapeutic implications of Curcumin

Curcumin exhibits significant potential in managing cartilage-related diseases like various arthritic and rheumatic conditions through its anti-apoptotic, anti-oxidant, anti-inflammatory, and anti-catabolic properties.

#### Cell survival and anti-apoptotic role

Curcumin plays a significant role in promoting cell survival in OA. Research has demonstrated that it is not only safe for chondrocytes but also effective in mitigating the cytotoxic effects induced by IL-1β. Specifically, curcumin has been shown to counteract mitochondrial changes, such as swelling and apoptosis, triggered by IL-1β stimulation. As shown in Fig. 2, it reduces apoptotic features while simultaneously enhancing the expression of anti-apoptotic factors like Bcl-2, Bcl-xL, and TRAF1. Furthermore, curcumin inhibits the activity of pro-apoptotic factors such as caspase-3, offering a protective effect against cell death [359].

Curcumin has demonstrated both anti-proliferative and apoptotic effects on synoviocytes at concentrations up to 10 µM, with higher doses (10–50 µM) reducing cell viability in a dose-dependent manner. This is significant in OA since synovial fibroblasts contribute to inflammation and joint destruction, making their apoptosis a potential therapeutic target. However, achieving the required curcumin concentration for apoptosis exceeds the plasma Cmax after oral administration, suggesting intra-articular injection as a possible alternative, though its safety needs further evaluation. Recent studies also show that curcumin protects human chondrocytes from IL-1β-induced damage. Pre-treatment with IL-1β followed by co-treatment with 50 µM curcumin reduced degenerative changes and inhibited caspase-3 activation, a key apoptosis marker, in a time-dependent manner [7].

**Fig. 6:**
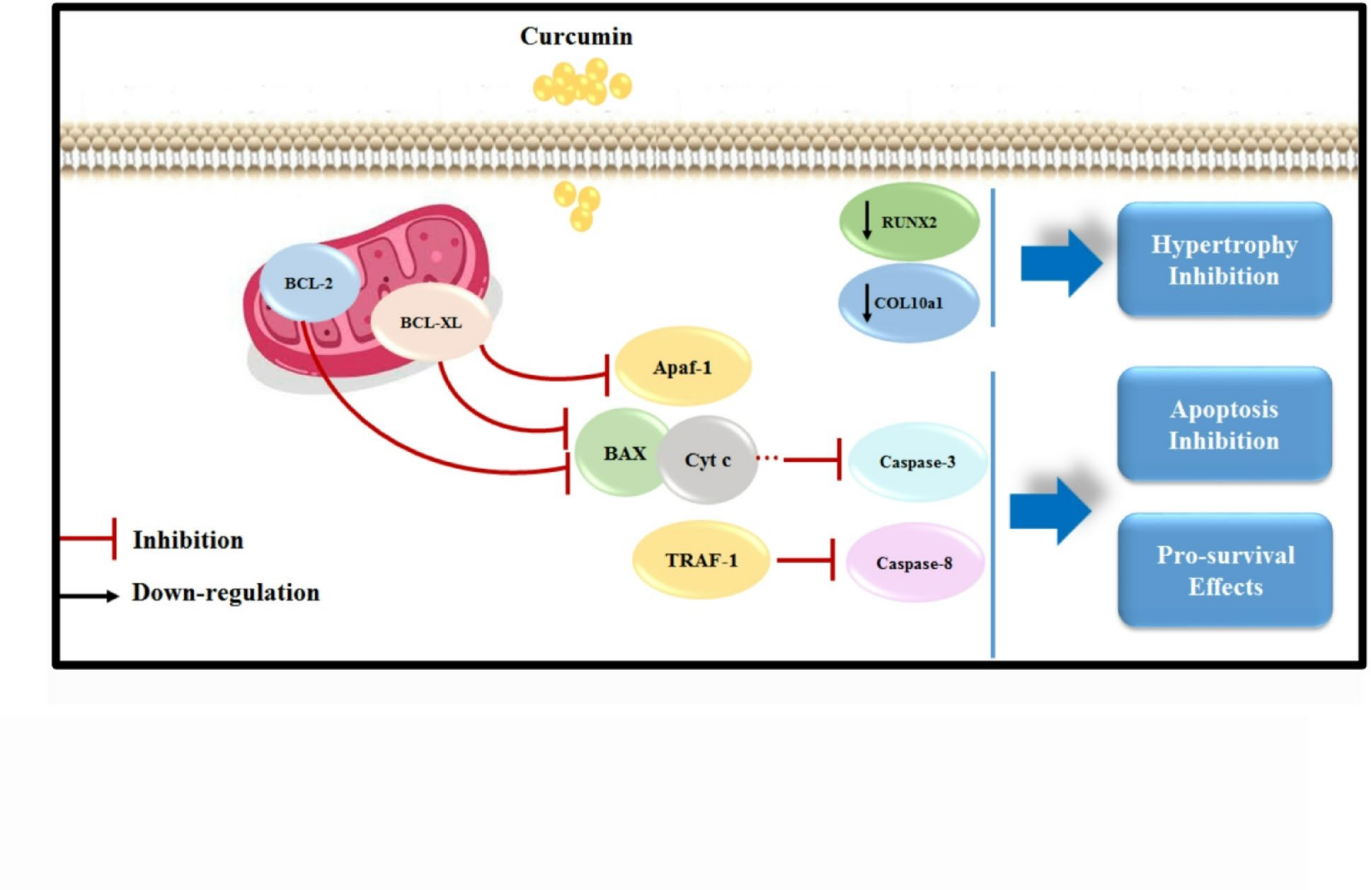
The role of curcumin in modulating cell survival and apoptosis pathways through regulation of anti-apoptotic genes (BCL-2, BCL-XL), pro-apoptotic factors (BAX, Cytochrome C Oxidase), and Caspase-3 inhibition, along with down-regulation of hypertrophic markers (RUNX2, COL10a1)

A study revealed that curcumin had no significant impact on the expression of chondrogenic markers such as Sox9 and Col2a1. However, it was observed to down-regulate hypertrophic markers, including Runx2 and COL10a1 (Fig. 6). Further investigations demonstrated that curcumin exerts its effects by inhibiting chondrocyte hypertrophy through the modulation of Indian hedgehog homolog (IHH) and Notch signaling pathways [360]. Curcumin has also demonstrated the ability to enhance the expression of the chondroprotective gene CITED 2 (Cbp/P300 interacting transactivator with Glu/Asp rich carboxy terminal domain 2), which appears to play a crucial role in the inhibition of NF-κB activity [361].These findings suggest that curcumin holds promising potential in maintaining cartilage homeostasis and preserving the chondrocyte phenotype, also as a therapeutic agent in managing OA by supporting chondrocyte survival and reducing inflammation-induced cellular damage [361].

#### Anti-inflammatory role

Inflammation is a key driver of damage in articular tissues, with subcellular signaling pathways playing a significant role in the progression of rheumatic diseases like OA and RA [362]. Among these pathways, the NF-κB transcription factor is critically involved in regulating genes associated with inflammatory responses. This protein complex is activated by various stimuli, including cytokines, free radicals, and microbial antigens [363], and contributes to processes such as inflammation, cartilage breakdown, cell proliferation, angiogenesis, and pannus formation [364]. Given its central role in these pathological mechanisms, NF-κB has become a promising target for therapeutic intervention [364]. Curcumin exhibits significant anti-inflammatory properties by targeting key signaling pathways and pro-inflammatory mediators. It inhibits IL-1β-stimulated MAP kinases, transcription factors like AP-1 and NF-κB, and reduces MMP-3 and MMP-13 expression in both human and bovine articular chondrocytes [365]. Additionally, curcumin blocks IL-17 and IL-18 signaling, thereby reducing cartilage breakdown through suppression of MMPs, aggrecanases, and VEGF [366]. Its anti-inflammatory effects are also mediated by inhibiting enzymes such as phospholipase A2, COX-2, 5-LOX, and suppressing NF-κB-dependent gene transcription, which regulates pro-inflammatory mediators like COX-2 and iNOS [7]. Unlike NSAIDs, curcumin uniquely inhibits COX-2 without affecting COX-1 and reduces NO production while being less potent on PGE2 [7]. Studies suggest that curcumin alleviates OA by decreasing inflammation and inflammatory markers like IL-1, TGF-beta, PGE2, and MMP-3 [336] .

Researchers identified seven hub genes linked to metabolic processes and the AMPK signaling pathway through Gene Ontology (GO) and KEGG pathway analyses in a study using curcumin in a sodium mono iodoacetate-induced rat knee OA model and an IL-1β-induced OA chondrocyte model. Curcumin treatment significantly alleviated OA symptoms by activating mitophagy, which maintained mitochondrial homeostasis through regulation of ROS levels, calcium signaling, ATP production, and mitochondrial membrane potential. Enhanced expression of mitophagy-related proteins was observed in both AC and chondrocytes. Validation experiments, supported by network pharmacology, highlighted the importance of mitophagy in curcumin’s protective effects on cartilage. These effects are mediated via the AMPK/PINK1/Parkin pathway, positioning curcumin as a promising therapeutic candidate for OA management [143]. A recent study utilized metabolomics and transcriptomics to investigate curcumin’s effects on human articular chondrocytes. Curcumin significantly reduced inflammatory markers, including IL-β, IL-6, and TNF-α. Metabolomic analysis identified 106 differential metabolites, while transcriptomics revealed 1050 differential mRNAs. Enrichment analysis highlighted seven key pathways, such as glycine, serine, and threonine metabolism; glycerolipid metabolism; and inositol phosphate metabolism. A total of 23 critical targets were identified within these pathways. The findings suggest that curcumin may alleviate OA by modulating glycine, serine, and threonine metabolism, inhibiting pyruvate production, and regulating glycolysis. These results provide valuable insights into curcumin’s therapeutic potential for OA [367].

Another study highlights the potential pharmaceutical value of combining curcumin and probucol for OA prevention and therapy. OA, a chronic joint disease associated with cholesterol accumulation in chondrocytes, can benefit from this combined treatment due to its ability to reduce cholesterol levels and protect chondrocytes from stress injury caused by inflammatory cytokines. The protective effects are linked to the inhibition of apoptosis and the suppression of the autophagy-related PI3K/Akt/mTOR signaling pathway. In vitro studies using immunofluorescence staining and in vivo experiments with immunohistochemistry staining confirmed these findings. The combination therapy was shown to block the PI3K/Akt/mTOR pathway, enhance COL-II expression, and suppress P62, MMP-3, and MMP-13 expression, thereby mitigating TNF-α-induced cartilage degradation. Additionally, it reduced ECM GAGs release and alleviated OA severity [187].

Curcumin demonstrates significant therapeutic potential in an OA mouse model by reducing the production of key inflammatory and catabolic mediators, including MMP-1, MMP-3, MMP-13, IL-1β, TNF-α, and ADAMTS5. Its mechanism of action involves the inhibition of I kappa B kinase (IKK) activation in chondrocytes, osteoblasts, and synovial cells, thereby preventing the phosphorylation of this kinase and subsequent activation of NF-kB. This inhibition suppresses the expression of pro-apoptotic genes such as caspase-3 in chondrocytes and reduces the formation of inflammatory mediators like lipoxygenases, COX-2, phospholipase A2, prostaglandin E2 (PGE2), IL-1β, IL-6, and IL-8 By blocking NF-kB signaling, curcumin decreases COL degradation induced by IL-1-stimulated chondrocytes, Furthermore, it inhibits TNF-α, a cytokine linked to increased cartilage resorption, as well as IL-6 and IL-1, which collectively suppress proteoglycan synthesis. As summarized in Fig. 3, these multifaceted effects highlight curcumin’s potential to mitigate inflammation and cartilage degradation in OA [361].

#### Anti-catabolic/anabolic role

Curcumin exhibits significant anti-catabolic and anti-inflammatory properties, making it a promising agent for maintaining cartilage integrity and counteracting joint tissue degradation, particularly in OA. By inhibiting NF-κB and activator protein 1 (AP-1), curcumin suppresses the expression of matrix MMPs such as MMP-1, MMP-3, MMP-9, and MMP-13, which are induced by inflammatory cytokines like IL-1β and oncostatin M (OSM). Studies have shown that curcumin effectively reverses IL-1β-induced downregulation of type II collagen and β1 integrin in chondrocytes, essential components for cartilage health. At concentrations of 10 µM, curcumin inhibited collagenase and stromelysin expression in rabbit chondrocytes, while at 50 µM, it suppressed NF-κB-mediated matrix degradation products and MMP-9 activity [7].

**Fig. 7:**
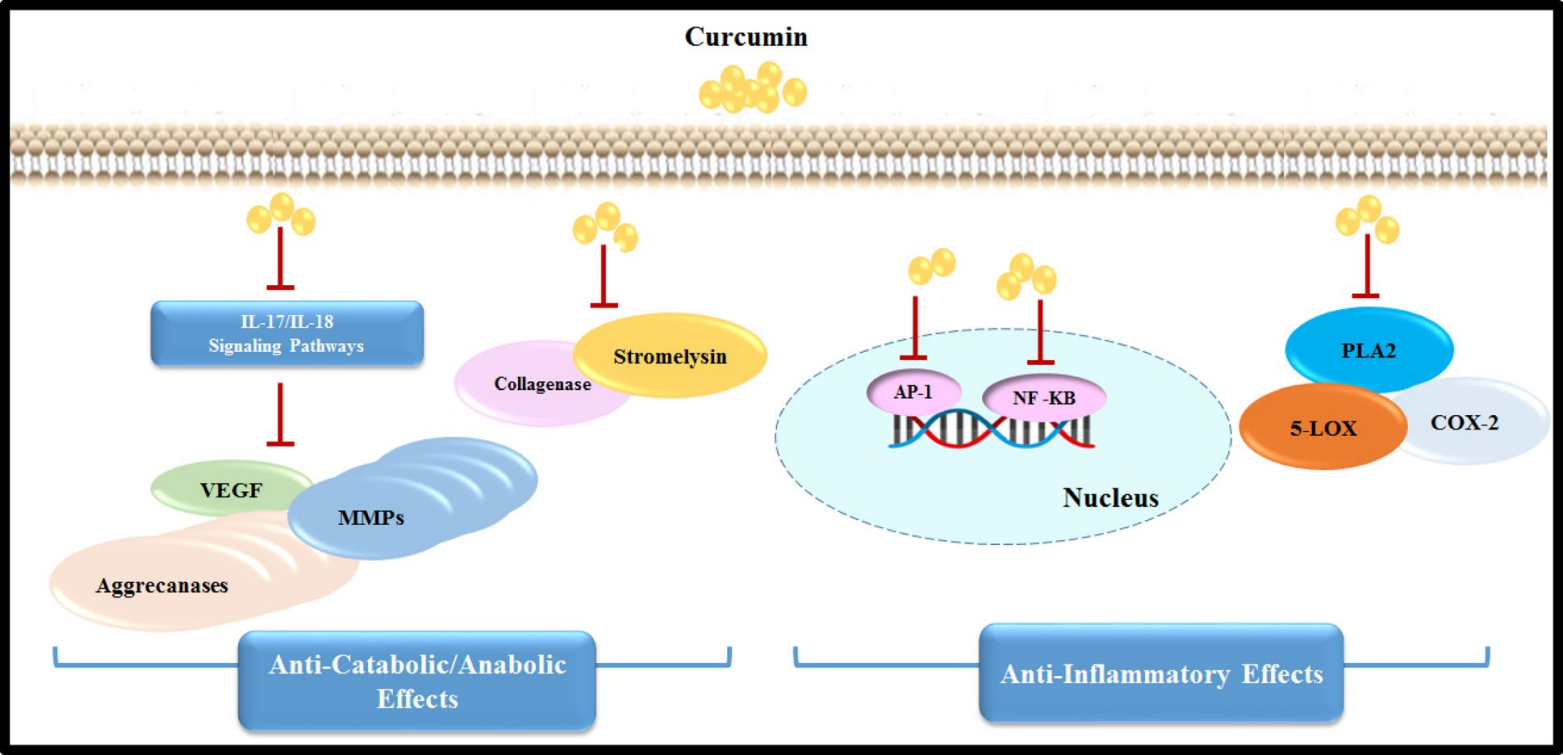
The role of curcumin in modulating inflammatory signaling pathways (IL-17/IL-18, NF-ΚB) and inhibiting pro-inflammatory enzymes (COX-2, s-LOX), along with regulating anabolic factors (VEGF) and suppressing catabolic enzymes (MMPs, Aggrecanases, Stromelysin, Collagenase) to maintain tissue degradation and repair balance

Notably, the synergistic effects of curcumin combined with resveratrol have demonstrated enhanced efficacy in reversing type II collagen inhibition. Furthermore, curcumin reduced GAG release in equine cartilage explantsand inhibited MMP synthesis (MMP-1, MMP-9 and MMP-13) in tenocytes stimulated by IL-1β [368]. Beyond its anti-catabolic effects, curcumin promotes chondrogenesis in MSCs under inflammatory conditions, highlighting its potential to restore the balance between anabolic and catabolic processes in OA tissues [369]. Collectively, these findings demonstrate curcumin’s capacity to prevent cartilage degradation, support ECM synthesis, and offer structural and functional benefits for joint health through its balanced modulation of degradation/repair pathways (Fig. 7).

### Advancements in clinical trials with Curcumin-Based therapies

Curcumin has been extensively studied for its potential therapeutic effects on cartilage-related diseases such as RA and OA. Clinical research indicates that curcumin possesses anti-inflammatory and antioxidant properties, which may help alleviate symptoms and slow disease progression in individuals suffering from these conditions. By targeting inflammatory pathways and reducing oxidative stress, curcumin could play a significant role in improving joint health and enhancing the quality of life for patients with RA and OA. A systematic review examined clinical trials focusing on turmeric and curcumin by conducting data searches on PubMed and Scopus using the terms “turmeric and clinical trials” and “curcumin and clinical trials.” The review identified 148 relevant studies for turmeric and 440 relevant studies for curcumin, demonstrating the extensive research conducted on these compounds. A summary of some of these clinical studies was provided (Table 3), shedding light on the growing interest in their potential therapeutic applications [370].

A one-month randomized, double-blind, placebo-controlled study [371] found a curcumin-rich herbomineral formulation improved pain and mobility in OA patients. More robust evidence comes from a study that explored the clinical efficacy and safety of Meriva^®^, a patented curcumin-phosphatidylcholine complex, in patients with primary knee OA over an 8-month period. The trial included 100 OA patients with grade 1 or 2 knee OA, divided into two groups: one receiving the “best available treatment” as prescribed by healthcare providers, and the other receiving the same treatment plus Meriva^®^. The Meriva^®^ group took two 500 mg tablets daily (equivalent to 200 mg curcuminoids/day), composed of a natural curcuminoid mixture (75% curcumin, 15% demethoxycurcumin, and 10% bisdemethoxycurcumin), phosphatidylcholine (40%), and microcrystalline cellulose (40%). Results showed significant improvements in pain, stiffness, joint function, and all WOMAC scores, including physical, social, and emotional functioning. Additionally, Meriva^®^ markedly reduced inflammation markers such as IL-1β, IL-6, sCD40L, sVCAM-1, and ESR. These findings highlight Meriva^®^’s potential as an effective adjunct therapy for managing OA symptoms and inflammation [18].

A study involving thirty patients diagnosed with OA, based on the ACR criteria and WOMAC, was conducted to evaluate the effects of curcumin. Participants were equally divided into two groups: an intervention group receiving 80 mg of Sinacurcumin^®^ daily and a placebo group, with both groups monitored over a 3-month period. Findings from the intervention group revealed significant improvements, including a marked reduction in the Visual Analog Score (VAS), C-reactive protein (CRP) levels, as well as frequencies of CD4 + and CD8 + T cells, Th17 cells, and B cells. Furthermore, there was a notable increase in Treg cells and a meaningful shift in the Treg/Th17 cell ratio favoring Treg lymphocytes. These results suggest that curcumin administration not only alleviated clinical symptoms but also exhibited immunomodulatory effects in OA patients, highlighting its potential therapeutic benefits [372].

A randomized double-blind placebo-controlled clinical trial investigated the impact of curcuminoid supplementation on inflammatory biomarkers in patients with mild-to-moderate knee OA over six weeks. The study involved 40 participants, with 19 receiving 1,500 mg/day of curcuminoids (divided into three doses) combined with 15 mg/day of piperine to enhance bioavailability, and 21 receiving a matched placebo. Serum levels of interleukins IL-4 and IL-6, tumor necrosis factor-α (TNF-α), TGF-β, high-sensitivity C-reactive protein (hs-CRP), and erythrocyte sedimentation rate (ESR) were measured at baseline and at the trial’s end. In the curcuminoid group, significant reductions were observed in IL-4, IL-6, and hs-CRP, while TNF-α, TGF-β, and ESR levels showed no significant changes. The placebo group also exhibited significant reductions in IL-4, IL-6 ,TNF-α, and TGF-β ,but hs-CRP and ESR remained unchanged. The magnitude of biomarker changes did not significantly differ between the groups. Although, clinical symptoms of OA improved in the curcuminoid-treated group, these improvements could not be attributed to systemic anti-inflammatory effects of the treatment [373].

Curene, a bioavailable turmeric Curcuma longa extract formulated with proprietary Aquasome technology, has demonstrated potential in managing knee OA due to its anti-inflammatory properties, which inhibit COX-2 and 5-lipoxygenase (5-LOX) enzymes. A randomized, double-blind, placebo-controlled study involving 50 subjects aged 40 to 75 years with unilateral or bilateral knee OA for over three months was conducted to evaluate its safety and efficacy. Participants were divided into two groups, with one receiving 500 mg of Curene daily and the other receiving a placebo. Efficacy was assessed using changes in Visual Analogue Scale (VAS) and WOMAC scores, while safety was evaluated through biochemical, hematological, and urine analyses. Of the 46 subjects who completed the trial, those treated with Curene showed statistically significant reductions in total WOMAC scores, subscale scores, and VAS scores compared to the placebo group. Additionally, Curene was found to be safe and well-tolerated, with no treatment-related adverse events reported. The study concluded that Curene provides significant and clinically meaningful improvements in pain relief, stiffness reduction, and physical functioning for individuals with knee OA, alongside an excellent safety profile. The trial is registered with the Clinical Trial Registry of India (CTRI/2017/07/009044) [374].

A randomized, double-blind, placebo-controlled study spanning 8 weeks evaluated the effects of a standardized curcumin extract (Curcugen^®^) on 101 adults with knee OA. Participants received either 500 mg of curcumin twice daily or a placebo, with outcomes assessed using measures such as the Knee Injury and Osteoarthritis Outcome Score (KOOS), knee pain ratings, Japanese Orthopedic Association Score for Osteoarthritic Knees (JOA), PROMIS–29, and performance-based tests like the 40-m fast-paced walk test, 6-min walk test, timed up-and-go test, and 30-s chair stand test. Results demonstrated that curcumin significantly reduced the KOOS knee pain score and numeric knee pain ratings compared to placebo. Additionally, curcumin showed greater improvements in the timed up-and-go test, 6-min walk test, and JOA total score, though no significant differences were observed for the 30-s chair stand test or 40-m fast-paced walk test. Notably, 37% of participants on curcumin reduced their use of pain-relieving medication compared to 13% on placebo [375].

A recent study investigated the efficacy of nanomicelle curcumin in alleviating symptoms of knee OA, aiming to enhance the oral bioavailability of curcumin. Conducted as a randomized, double-blind controlled trial, the study involved administering 40 mg of nanocurcumin capsules every 12 h for six weeks to the intervention group, while the control group received a placebo. The final analysis included 36 participants in the nanocurcumin group and 35 in the placebo group. Researchers utilized WOMAC to assess symptoms at the start of the study and after six weeks. Baseline characteristics such as gender, age, Kellgren score, and disease duration were comparable between the groups. Results revealed a significant reduction in overall WOMAC scores, as well as in pain, stiffness, and physical activity subscales, for the nanocurcumin group compared to the placebo group, suggesting its potential effectiveness in managing KOA symptoms [376].

Researchers created Theracurmin, a water-dispersible form of curcumin with 27 times higher bioavailability than curcumin powder. A randomized, double-blind, placebo-controlled study assessed its effects on knee OA. Fifty patients aged over 40 with Kellgren-Lawrence grade II or III osteoarthritis received either a placebo or Theracurmin (180 mg/day of curcumin) for 8 weeks. Knee symptoms were evaluated at multiple intervals using various measures, including the Japanese KOA Measure and knee pain visual analog scale (VAS). At 8 weeks, the Theracurmin group showed significantly lower VAS scores compared to the placebo group, except for patients with initial VAS scores of 0.15 or less. Theracurmin also reduced reliance on celecoxib more effectively than the placebo. Blood biochemistry analyses revealed no major side effects, indicating Theracurmin’s safety and potential as a therapeutic option for knee osteoarthritis [377].

A recent randomized, double-blind, controlled trial investigated the effects of curcumin nanomicelle on clinical symptoms in RA patients. Sixty-five participants were divided into two groups: one receiving curcumin nanomicelle (40 mg) and the other a placebo, both administered three times daily for 12 weeks. Researchers assessed the Disease Activity Score of 28 joints, tender joint count (TJC), swollen joint count (SJC), and ESR at baseline and post-intervention. While both groups showed significant within-group reductions in DAS-28, TJC, and SJC compared to baseline, the differences between the curcumin and placebo groups were not statistically significant. However, the improvements were slightly greater in the curcumin group. No notable changes in ESR were observed between the groups. In conclusion, while curcumin nanomicelle supplementation led to some positive trends in disease activity and joint symptoms, the effects were not significantly different from the placebo [378].

A clinical study investigated the efficacy of a novel, highly bioavailable curcumin formulation embedded in a natural turmeric matrix for managing RA. This randomized, double-blind, placebo-controlled trial included three parallel groups, each consisting of 12 participants who received either a placebo, 250 mg, or 500 mg of the curcumin product twice daily for 90 days. The study assessed patients using multiple metrics, including the ACR response, VAS, CRP, DAS28, ESR, and rheumatoid factor (RF). Results revealed that both low and high doses of curcumin significantly alleviated RA symptoms compared to the placebo group, with marked improvements observed in ESR, CRP, RF, and other clinical measures. These findings suggest that curcumin in a turmeric matrix function as a potent analgesic and anti-inflammatory agent for RA management at doses as low as 250 mg twice daily. Furthermore, the study confirmed that the curcumin product was well tolerated by all participants and did not produce any adverse side effects [379].

A placebo-controlled, randomized study investigated the effectiveness of CuroWhite™, a novel hydrogenated curcuminoid formulation, in managing RA. The study included 24 RA patients who were randomly assigned in a 1:1:1 ratio to receive either 250 mg or 500 mg of CuroWhite, or a placebo, administered as one capsule daily for three months. Key outcomes measured included the ACR response, disease activity assessed through the DAS28 score, and physical function changes evaluated via ESR, CRP, and RF levels before and after the trial. The findings demonstrated that both low and high doses of CuroWhite led to statistically significant improvements in clinical symptoms compared to the placebo group. Specifically, notable reductions were observed in DAS28 (50–64%), VAS (63–72%), ESR (88–89%), CRP (31–45%), and RF (80–84%) values, alongside improved ACR responses. These results indicate that CuroWhite effectively reduces inflammation and serves as an analgesic and anti-inflammatory agent for RA management. Furthermore, the study confirmed that CuroWhite is highly effective, well-tolerated, and safe, with no serious side effects reported [380].

A clinical study investigated the safety and efficacy of curcumin, both as a standalone treatment and in combination with diclofenac sodium, in patients with active RA. A total of 45 RA patients were randomized into three groups to receive either curcumin (500 mg), diclofenac sodium (50 mg), or a combination of the two. The primary outcome measure was the reduction in DAS28, while secondary outcomes included improvements in ACR criteria for joint tenderness and swelling. All three groups demonstrated statistically significant reductions in DAS scores; however, the curcumin group exhibited the greatest improvement in both DAS and ACR scores (ACR 20, 50, and 70), outperforming the diclofenac sodium group. Notably, curcumin treatment was found to be safe, with no reported adverse events. These findings provide preliminary evidence for the safety and superior efficacy of curcumin in managing active RA, underscoring the need for larger-scale studies to confirm its therapeutic potential in RA and other arthritic conditions [20].

Curcumin has been studied for its potential benefits in patients with RA, as highlighted by Chandran and Goel (2012). In their study, curcumin (500 mg) demonstrated safety and was not associated with any adverse events. Moreover, it was found to be the most effective treatment for improving the disease activity score and the ACR score, whether administered alone or in combination with diclofenac sodium (50 mg) [7].

A clinical trial investigated the impact of curcumin supplementation on metabolic, inflammatory, and obesity-related parameters in women with RA. This randomized, double-blind, placebo-controlled study involved 48 participants who were administered either 500 mg of curcumin daily or a placebo for 8 weeks. Measurements, including fasting blood samples, anthropometric data, dietary intake, and physical activity levels, were collected at the beginning and end of the study. Results revealed that curcumin supplementation significantly reduced homeostatic model assessment for insulin resistance (HOMA-IR), erythrocyte sedimentation rate, high-sensitivity C-reactive protein levels, triglycerides, body weight, body mass index, and waist circumference compared to the placebo group. Conversely, HOMA-IR and triglyceride levels increased within the placebo group. However, no significant changes were observed in fasting blood sugar, insulin levels, other lipid profiles, or vastatin levels in either group. These findings suggest that curcumin could be a beneficial component of an integrated approach to managing metabolic factors, inflammation, and obesity in women with RA [381].

Recently, a Level I, double-blinded, placebo-controlled, prospective study evaluated the efficacy and safety of Theracurmin, delivering 180 mg of curcumin daily, in patients undergoing mosaicplasty for knee chondral or osteochondral diseases over a 12-month period. Of the 50 enrolled patients aged over 20, 43 completed the study, comprising 14 men and 29 women with a mean operative age of 59.5 years. Clinical outcomes were assessed using Japanese Orthopaedic Association knee osteoarthritis score (JOA), VAS, and Japanese Knee Osteoarthritis Measure (JKOM) scores, alongside T2 mapping via MRI for chondroprotective effects and intraoperative acoustic evaluation. Blood curcumin levels were measured at 0, 3, 6, and 12 months. While JOA, VAS, and JKOM scores significantly improved post-surgery compared to preoperative levels, no significant differences were observed between the Theracurmin and placebo groups. However, second-look arthroscopy revealed significantly better cartilage stiffness and surface roughness in the Theracurmin group. Curcumin blood levels were consistently higher in the Theracurmin group without adverse events. In conclusion, Theracurmin demonstrated significant chondroprotective benefits and comparable clinical outcomes to placebo, supporting its safety and potential as a therapeutic option. This double-blinded, placebo-controlled study provides Level I evidence for its use [19].

**Table 3:** Summary of clinical trials on Curcumin-Based therapies for OA and RA

Condition	Intervention	Duration	Key findings	Refs.
OA	Curcumin-rich herbomineral formulation	1 month	Improved pain and mobility in OA patients.	[227]
Knee OA (Grade I-II)	Meriva^®^ (200 mg curcuminoids/day) vs. best available treatment	8 months	Significant improvements in WOMAC scores, reduced IL-1β, IL-6, ESR.	[19]
OA	Sinacurcumin^®^ (80 mg/day) vs. placebo	3 months	Reduced VAS, CRP, CD4+/CD8 + T cells; increased Treg/Th17 ratio.	[229]
Mild-moderate knee OA	Curcuminoids (1,500 mg/day + piperine) vs. placebo	6 weeks	Reduced IL-4, IL-6, hs-CRP; no change in TNF-α, TGF-β, ESR.	[230]
Knee OA	Curene (500 mg/day) vs. placebo	3 months	Improved WOMAC, VAS scores; safe and well-tolerated.	[231]
Knee OA	Curcugen^®^ (500 mg twice daily) vs. placebo	8 weeks	Reduced KOOS pain, improved JOA score, reduced pain medication use.	[232]
Knee OA	Nanomicelle curcumin (40 mg twice daily) vs. placebo	6 weeks	Significant reduction in WOMAC scores (pain, stiffness, function).	[229]
Knee OA (Grade II-III)	Theracurmin (180 mg/day) vs. placebo	8 weeks	Lower VAS scores, reduced celecoxib use, no major side effects.	[233]
RA	Nanomicelle curcumin (40 mg thrice daily) vs. placebo	12 weeks	Non-significant trends in DAS-28, TJC, SJC improvement.	[235]
RA	Turmeric-matrix curcumin (250 mg/500 mg twice daily) vs. placebo	90 days	Improved ACR response, DAS28, ESR, CRP, RF; well-tolerated.	[236]
RA	CuroWhite™ (250 mg/500 mg daily) vs. placebo	3 months	Reduced DAS28 (50–64%), VAS (63–72%), ESR (88–89%), CRP (31–45%).	[237]
Active RA	Curcumin (500 mg) vs. diclofenac (50 mg) vs. combination	8 weeks	Curcumin outperformed diclofenac in DAS28 and ACR scores; no adverse events.	[21]
RA	Curcumin (500 mg) ± diclofenac (50 mg)	8 weeks	Curcumin most effective for DAS and ACR improvement.	[7]
RA (women)	Curcumin (500 mg/day) vs. placebo	8 weeks	Reduced HOMA-IR, ESR, hs-CRP, triglycerides, BMI, waist circumference.	[238]
Post-mosaicplasty knee	Theracurmin (180 mg/day) vs. placebo	12 months	Improved cartilage stiffness (arthroscopy); no clinical score differences.	[20]

## Future prospects

Curcumin has garnered significant attention for its diverse therapeutic effects, including antimicrobial, anti-inflammatory, anti-aging, anticancer, and digestive health benefits. Research has particularly highlighted its role in modulating chondrocyte proliferation and differentiation, a critical process in cartilage repair. Although curcumin does not directly enhance chondrocyte activity, it significantly reduces inflammation and oxidative stress by inhibiting key inflammatory pathways. These properties position curcumin as a promising candidate for cartilage tissue engineering, with the potential to improve patient outcomes by reducing reliance on steroidal anti-inflammatory treatments.

However, it is important to note that while various scaffolds incorporating curcumin have been developed and studied, there is limited direct comparison between their effectiveness or suitability for specific applications. For instance, scaffolds such as Cur-SLNs/GelMA, MWCNTs/ COL/Cur and curcumin/silk each demonstrate unique advantages in terms of mechanical properties, biocompatibility, and therapeutic outcomes. Future studies should focus on systematically comparing these scaffolds in similar experimental settings to determine their relative efficacy for specific clinical applications, such as cartilage repair, intervertebral disc regeneration, or tendon healing. Such comparisons would provide valuable insights into optimizing scaffold design and selecting the most appropriate platform for targeted tissue engineering applications.

Despite these advancements, several challenges remain in the field of cartilage tissue engineering. Current methods often produce ECM with suboptimal biomechanical properties and limited integration with host tissues, which can lead to tissue degeneration over time. To fully harness curcumin’s therapeutic potential, future research should focus on addressing its limitations, such as poor bioavailability and stability. Advanced delivery systems, including nanoparticles, liposomes, and hydrogels, could enhance curcumin’s efficacy and enable targeted release. Additionally, exploring its synergistic effects with other bioactive compounds, such as growth factors and stem cells, may improve cartilage repair outcomes.

Further in vivo and clinical studies are essential to evaluate curcumin’s safety and efficacy in humans. Investigating its molecular mechanisms, particularly its anti-inflammatory and antioxidant effects, could provide deeper insights into its chondroprotective properties. Optimizing dosage and formulations will also be crucial for maximizing its regenerative potential in clinical applications. Moreover, the development of enhanced scaffold designs that incorporate both mechanical and biological cues is paramount for improving the efficacy of CTE strategies. By bridging these gaps, curcumin-based therapies, combined with innovative scaffold technologies and controlled release mechanisms, could revolutionize regenerative medicine and lead to effective therapies for cartilage repair. Ongoing research into composite materials, scaffold architecture, and delivery systems holds great promise for advancing CTE and improving the quality of life for patients worldwide.

The integration of curcumin into advanced biomaterial-based scaffolds and DDSs has emerged as a groundbreaking strategy to address its pharmacokinetic limitations, including poor solubility, rapid metabolism, and low bioavailability. By leveraging natural and synthetic polymers such as CH, PCL, and PLGA, researchers have engineered innovative platforms that enhance curcumin’s therapeutic potential for cartilage regeneration. For instance, chitosan-based hydrogels modified with CNC or MMT demonstrated pH-sensitive drug release and improved mechanical stability, enabling localized delivery in simulated gastrointestinal and inflammatory environments. Similarly, injectable chitosan hydrogels crosslinked with genipin or trisodium phosphate achieved sustained curcumin release with reduced initial burst effects, offering a promising solution for targeted cartilage repair. PCL scaffolds further expanded curcumin’s utility by providing prolonged release kinetics, critical for treating chronic inflammatory conditions. Electrospun PCL nanocapsules preserved curcumin’s bioactivity under gastrointestinal conditions, while PCL-based tracheal scaffolds loaded with curcumin suppressed fibrosis and granulation hyperplasia in vivo, promoting functional cartilage regeneration. Hybrid systems, such as chitosan/β-glycerophosphate hydrogels reinforced with bacterial cellulose and curcumin-loaded PCL microspheres, synergized structural support with anti-inflammatory and antibacterial properties, achieving enhanced chondrocyte density in rabbit models. Combinatorial approaches, including PLGA nanoparticles co-delivering curcumin and siRNA targeting HIF-2α, or DEX-Cur formulations, showcased dual anti-inflammatory and chondroprotective effects, effectively mitigating OA progression in preclinical studies. Despite these advancements, challenges such as biodegradation-induced inflammation, scalability of fabrication techniques, and long-term biocompatibility require further optimization. Future research should prioritize clinical translation by refining targeted delivery mechanisms, such as dCOL2-CM-Cur-PNPs’ cartilage-homing phospholipids, and validating safety profiles in human trials. By bridging nanotechnology, biomaterial engineering, and regenerative medicine, curcumin-based DDSs represent a paradigm shift in cartilage tissue engineering, offering a multifaceted solution to inflammation management, chondrocyte preservation, and structural restoration in degenerative joint diseases.

The extensive body of clinical trials on curcumin-based therapies underscores its potential as a promising therapeutic agent for managing OA and RA. Studies consistently demonstrate that curcumin, particularly in bioavailability-enhanced formulations like Meriva^®^, Curene^®^, Theracurmin^®^, and nanomicelle/nanocurcumin preparations, effectively reduces pain, stiffness, and inflammation while improving joint function. For instance, Meriva^®^ significantly lowered WOMAC scores and inflammatory markers (IL-1β, IL-6, ESR) in OA patients over 8 months, while Curene^®^ and Theracurmin^®^ achieved clinically meaningful symptom relief and cartilage protection. Similarly, curcumin formulations like CuroWhite™ and Sinacurcumin^®^ showed immunomodulatory effects in RA, reducing CRP, ESR, and DAS28 at low doses (250–500 mg/day). Despite these benefits, variability exists in trial outcomes. Some studies reported no significant systemic anti-inflammatory effects (e.g., curcuminoids with piperine in OA or limited superiority over placebo in specific metrics (e.g., nanomicelle curcumin in RA). These discrepancies highlight the need for standardized dosing, longer trial durations, and larger cohorts to validate efficacy. Importantly, curcumin’s safety profile remains exceptional across studies, with no serious adverse events reported even at high doses. Collectively, the evidence positions curcumin as a viable adjunct or alternative to conventional therapies, particularly for patients seeking safer, natural options. Future research should prioritize optimizing bioavailability, identifying responder subgroups, and exploring synergistic effects with existing treatments. As formulations evolve, curcumin’s dual role as an anti-inflammatory and chondroprotective agent could revolutionize the management of chronic joint diseases.

## Conclusion

In summary, curcumin has emerged as a highly promising therapeutic agent for cartilage repair due to its potent anti-inflammatory and antioxidant properties, primarily mediated through the inhibition of key pathways like NF-κB, MMP-9, and COX. While it does not directly enhance chondrocyte proliferation, its ability to modulate the destructive joint environment positions it as an excellent candidate for regenerative strategies. Significant advancements have been made in overcoming its poor bioavailability through innovative DDSs, such as nanoparticles, hydrogels (e.g., CH, GelMA), and synthetic scaffolds (e.g., PCL, PLGA). These platforms enable targeted, sustained release, enhancing curcumin’s therapeutic efficacy. A substantial body of clinical evidence further supports its utility, demonstrating that bioavailable formulations like Meriva^®^ and Theracurmin^®^ effectively reduce pain, improve joint function, and exhibit an exceptional safety profile in managing OA and RA.

**Ethics declarations**.

## Data Availability

No datasets were generated or analysed during the current study.
